# Tissue-layer-resolved proteome landscape of Crohn’s disease strictures highlights potential drivers of fibrosis progression

**DOI:** 10.1172/jci.insight.202461

**Published:** 2026-02-10

**Authors:** Johannes Alfredsson, Carina Sihlbom Wallem, Maja Östling, Hanna de la Croix, Elinor Bexe-Lindskog, Mary Jo Wick

**Affiliations:** 1 Department of Microbiology and Immunology, Institute of Biomedicine, and; 2 Proteomics Core Facility, Sahlgrenska Academy, University of Gothenburg, Gothenburg, Sweden.; 3 Department of Clinical Pathology, Sahlgrenska University Hospital, Gothenburg, Sweden.; 4 Forensic Medicine Department, Swedish National Board of Forensic Medicine, Gothenburg, Sweden.; 5 Department of Surgery, IBD-SURG-Inflammatory Bowel Disease Surgical Research Group, Institute of Clinical Sciences, Sahlgrenska Academy, University of Gothenburg, Sweden.; 6 Department of Surgery, Sahlgrenska University Hospital, Region Västra Götaland, Gothenburg, Sweden.; 7 Department of Surgery, Institute of Clinical Sciences, Sahlgrenska Academy, University of Gothenburg, Gothenburg, Sweden.

**Keywords:** Cell biology, Gastroenterology, Fibrosis, Inflammatory bowel disease, Proteomics

## Abstract

The chronic inflammation of Crohn’s disease frequently leads to fibrosis and muscular hypertrophy of the intestinal wall. This often culminates in strictures, a serious condition lacking directed therapy. Severe pathological changes occur in the submucosa and muscularis propria intestinal wall layers of strictures, yet stricture-associated proteome changes in these layers is unexplored. We perform unbiased proteomics on submucosa and muscularis propria microdissected from transmural sections of strictured and nonstrictured ileum. Proteome changes in strictured submucosa reflected a transition from homeostasis to tissue remodeling, inflammation, and smooth muscle changes. Top submucosal features included reduced vascular components and lipid metabolism proteins accompanied by increased proteins with immune-, ECM-, or stress-related functions, including CTHRC1, TNC, IL-16, MZB1, and TXNDC5. In parallel, predominant changes in strictured muscularis propria included increased ECM (POSTN) and immune (mast cell CPA3) proteins alongside decreased proteins with lipid metabolic, mitochondrial, or key muscle functions. Finally, trends of differentially expressed proteins along nonstrictured submucosa suggest progressive profibrotic tissue remodeling and muscle expansion as proximity to strictures increases. The comprehensive proteome map presented here offers tissue-layer-resolved insight into the stricture microenvironment and potential drivers of fibrotic disease, providing a valuable resource to fuel biomarker and therapeutic target research.

## Abstract 2

Proteome landscape of Crohn’s disease intestinal strictures reveals tissue layer-specific changes, offering insight into potential drivers of fibrosis to fuel research on biomarkers and therapeutic targets.

## Graphical abstract

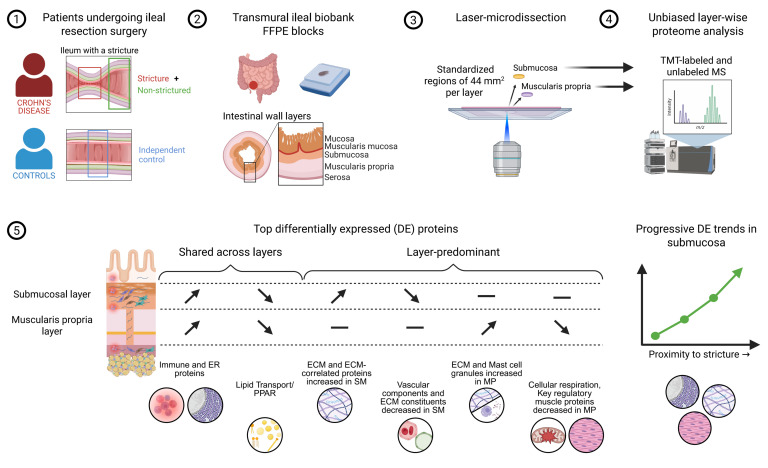

## Introduction

Crohn’s disease (CD) is a chronic inflammatory disease affecting the gastrointestinal tract characterized by chronic inflammation and progressive destruction of intestinal tissue (1). In many patients, the inflammation is further complicated by fibrosis and muscular hypertrophy of the intestinal wall, leading to progressive bowel wall thickening and lumen narrowing (2–6). As the fibrotic remodeling advances, severe bowel obstruction ultimately develops — a condition called stricturing that often requires urgent endoscopic or surgical intervention (6–8).

Despite advances in antiinflammation therapies (9), which may provide short-term benefits for a subset of patients with symptomatic strictures (9), current therapies fail to prevent stricture formation. While recently introduced JAK inhibitors hold promise (10), long-term data regarding impeding strictures are lacking. Attempts to develop targeted noninvasive therapies for strictures have thus far been unsuccessful (5, 8, 11, 12). Strictures, which often require repeated surgeries throughout life, thus remain a clinical challenge. Clarifying the pathogenetic mechanisms driving fibrotic stricture formation is of high clinical relevance.

Studies using primary intestinal tissue from strictured regions, or cells isolated from strictures of patients with CD, to advance understanding of intestinal strictures are relatively few. Moreover, many such studies are limited to endoscopic mucosal biopsies, which sample only the superficial intestinal layer. As such, these lack the full depth of the intestinal wall and poorly recapitulate the full extent of the stricturing disease, which traverses the full wall thickness (2, 3, 13). Only recently, the transmural nature of CD stricturing disease has begun to be explored, providing insight into the pathogenesis using full-thickness surgical tissue (14–21). Indeed, the layered architecture of the intestinal wall consists of distinct anatomical compartments differing in cellular composition and function. Moreover, the central histopathological features of strictures, fibrosis and muscle layer expansion, primarily affect the deeper wall layers, particularly the submucosa (SM) and muscularis propria (MP) (2, 3, 22–25). Despite their critical role in stricture formation, the proteomic landscape of these tissue layers in human strictured (STR) tissue is largely unexplored.

The SM and MP layers consist of different microenvironments and spatial niches that can only be properly addressed through tissue-layer-resolved analysis. However, analysis of proteins in a tissue-layer-wise fashion is challenged by the difficulty of precisely isolating the layers. Laser microdissection (LMD) offers a valuable solution by enabling microscopic separation of intact anatomical layers with precision. Unlike cell dissociation protocols, which can result in the loss of certain cell types, LMD retains intact tissue architecture, including cells and extracellular matrix (ECM) components, and provides a representative snapshot of the in situ tissue. LMD thus ensures analysis of the complete cell and ECM composition of the tissue being analyzed.

Here we perform, for the first time to our knowledge, an unbiased proteomic analysis of microdissected SM and MP layers from ileal tissue from patients with CD. Using 2 mass spectrometry (MS) methods in parallel, we generate robust, tissue-layer-wise insight into proteome changes in SM and MP on architecturally preserved STR tissue. Tissue-layer-specific changes in immune and ECM proteins, as well as muscular, vascular, and endothelial changes, were evident in strictures, with several proteins being highly differentially expressed. Moreover, concordant and discordant changes in protein expression shared across intestinal wall layers were apparent, suggesting interlayer dynamics in stricture formation. The study provides what we believe is unique, tissue-layer-resolved insight into the stricture microenvironment, highlights potential drivers of fibrotic disease, and provides a valuable resource to fuel research toward identification of biomarkers and therapeutic targets.

## Results

### MS of LMD intestinal wall reveals layer-specific proteome deviations in STR tissue.

**Figure 1:**
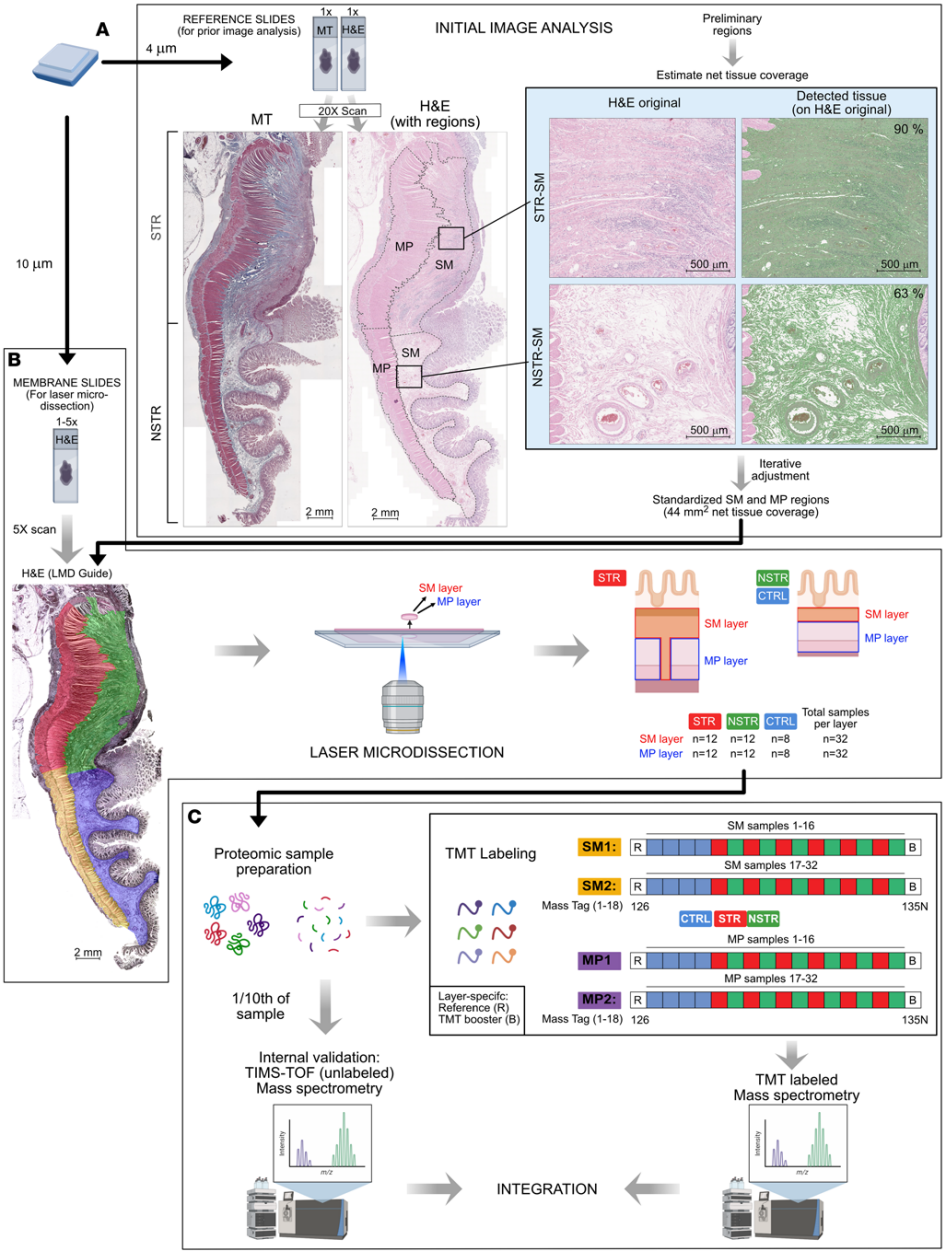
Overview of the workflow. The figure illustrates the workflow from FFPE block preparation through (**A**) initial imaging analysis, (**B**) laser microdissection (LMD) of submucosa (SM) and muscularis propria (MP) tissue layers, and (**C**) layer-wise mass spectrometry (MS) analysis. (**A**) Reference slides from each FFPE block were stained and scanned for initial image analysis. Given the “dense” versus “loose” tissue density in STR and NSTR samples (right), we developed an image-based method to identify one SM and one MP region from each sample to standardize tissue content of 44 mm^2^ across the cohort. Preliminary region outlines were drawn, and the net foreground tissue coverage was estimated using image analysis. Region sizes were then iteratively adjusted until reanalysis showed tissue coverage of 44 mm^2^ was obtained (see Supplemental Methods). The percentages inside the detected tissue overlays to the right indicate the foreground-to-background area ratio (“density”) detected in the tissue scan that was subsequently adjusted for by our standardization. (**B**) After standardized regions were identified, LMD slides were prepared from additional serial sections, scanned for use in creating an LMD guide, and stored until LMD. The standardized regions were redrawn onto these prescanned LMD slide images in the analysis software and used as visual guides during subsequent LMD. At the LMD microscope, the outlines of the standardized 44 mm^2^ tissue regions were laser microdissected to extract SM and MP layers for proteomic analysis. As illustrated in the schematic, the final sample cohort comprised SM and MP dissected from both STR and NSTR regions of 12 patients with CD and 8 CTRL individuals, yielding a total of 32 samples per layer. (**C**) The 64 LMD samples were prepared and analyzed using TMT-labeled MS. The number of samples exceeded the unique barcodes (TMT labels) available and were thus analyzed as 2 separate sets of 16 samples per layer (SM1 and SM2; MP1 and MP2; see Supplemental Methods 6 and 7). For internal validation, one-tenth of the volume of each sample was set aside before TMT labeling and used for label-free proteomics using TIMS-TOF-MS. The results from the 2 MS methodologies were integrated for robust downstream analysis at the combined level. MT, Masson’s trichrome.

The SM and MP undergo marked pathological changes in CD-related intestinal strictures, yet proteomic profiling of these layers in STR tissue has not been reported. To address this, we used LMD to isolate 44 mm^2^ of net tissue coverage of the SM and MP layers from STR and control tissues (Figure 1, A and B, Supplemental Figure 1, A–C, and Supplemental Methods; supplemental material available online with this article; https://doi.org/10.1172/jci.insight.202461DS1) followed by tandem mass tag labeling and MS (TMT-MS) (Figure 1C). In total, 3,350 proteins were quantified in the SM layer and 2,612 in the MP layer (Supplemental Figure 2A). Across both tissue layers, a total of 3,724 unique proteins were identified and quantified, with the majority (60%) detected in both layers (Supplemental Figure 2B).

Principle component analysis (PCA) of the TMT-MS data revealed a clear separation of STR from control samples along PC1 (Supplemental Figure 2, C and D), suggesting distinct, stricture-associated proteomic profiles in both layers. Furthermore, differential expression (DE) analysis within each tissue layer showed both the highest number and the largest magnitude of DE proteins in the STR versus control (STRvCTRL) comparison in the SM layer (Supplemental Figure 2E), consistent with the PCA (Supplemental Figure 2C). The MP layer had fewer DE proteins compared with the SM (Supplemental Figure 2, E and F). To strengthen and validate the TMT-MS data, an aliquot (one-tenth of the volume) of each sample was removed prior to TMT labeling for parallel analysis using label-free trapped ion mobility spectrometry with time-of-flight mass spectrometry (TIMS-TOF-MS) technology (Figure 1C). TIMS-TOF-MS demonstrated consistency with the global trends observed in the TMT-MS data and identified proteins not found with TMT-MS (Supplemental Results 1 and Supplemental Figure 2, G–L). Overall, the data obtained using 2 parallel MS technologies revealed proteome differences between STR and CTRL tissues within both layers, with layer-specific variations in the magnitude of these differences.

### Proteome changes in STR SM reflect a transition from homeostasis to tissue remodeling, inflammation, and smooth muscle changes.

**Figure 2:**
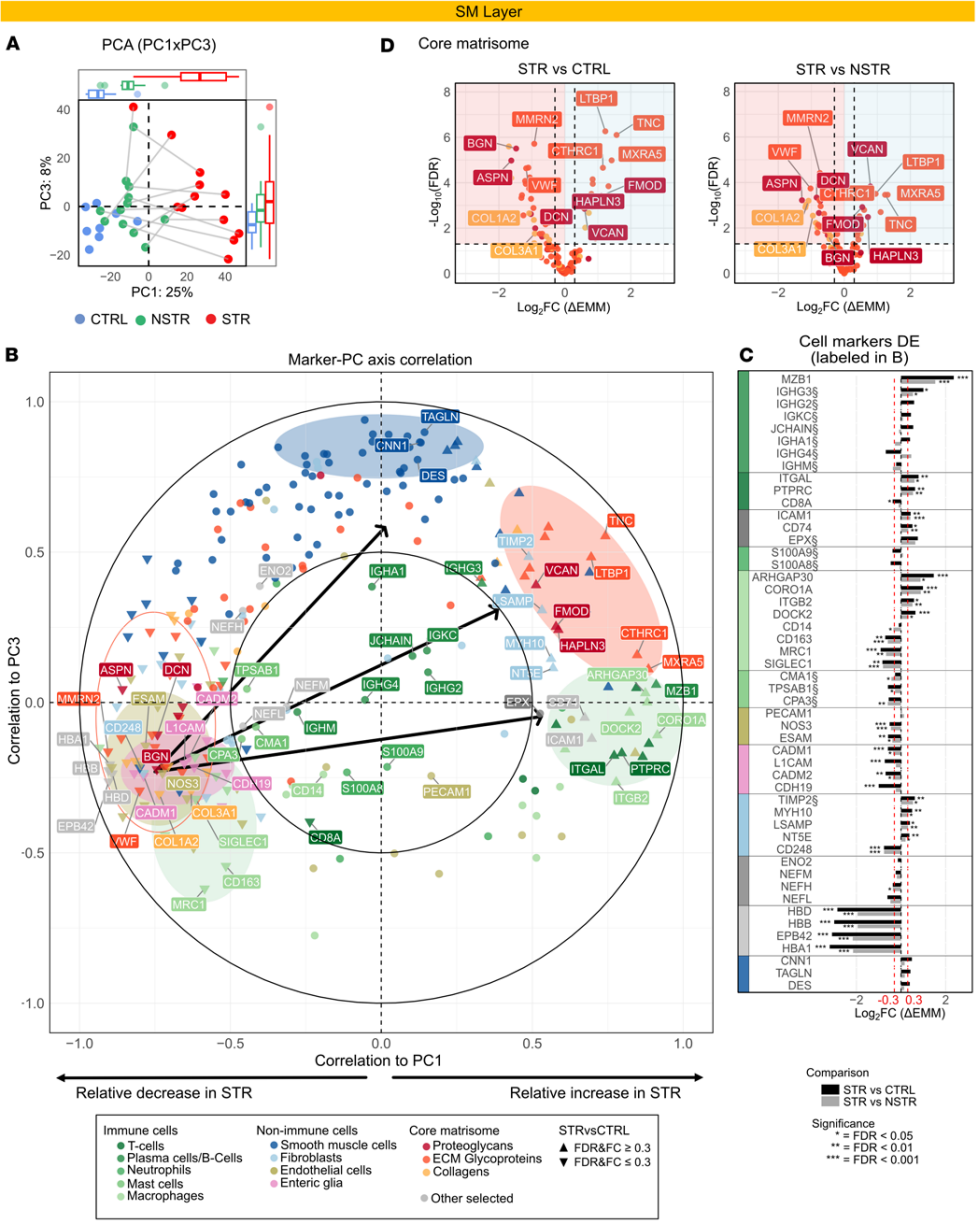
Changes in markers for immune function, steady-state structures, and tissue remodeling characterize the SM of STR tissue. (**A**) PCA plot (PC1 vs. PC3) with axis PC score box-and-whisker plots (top and right) for batch-corrected SM TMT data with complete observations. The percentage in the axis titles indicates the proportion of variance explained by each PC. Samples are color coded by tissue type, and STR and NSTR samples from the same individual are connected by lines. Axis box-and-whisker plots show the median (center line), IQR (box), whiskers extending to the smallest and largest values within 1.5 times the IQR, and outliers plotted as individual points. (**B**) Plot visualizing Pearson’s correlation coefficients between markers and PC1/PC3. Markers are color coded by assigned categories indicated below the plot with differentially expressed (DE) proteins indicated by triangles. Marker clusters and representative proteins discussed in the text are highlighted with ellipses and labels, respectively. (**C** and **D**) DE analysis results for markers from STRvCTRL and STRvNSTR comparisons within the SM layer. DE was tested using linear mixed-effects models with model-based contrasts of estimated marginal means (EMM), with degrees of freedom estimated using the Kenward-Roger method; *P* values were adjusted for multiple testing using the Benjamini-Hochberg method to control the false discovery rate (FDR). The coloring and labeling of individual proteins in **C** and **D** is consistent with **B**. (**C**) Bar plots showing DE results for the cell markers labeled in **B**, arranged by cell type annotation and colors according to the bottom of **B**. (**D**) Volcano plots of core matrisome proteins.

To gain insight into processes in the SM driving the separation of STR from CTRL tissue, we analyzed the correlation between PC axes and cell markers (Figure 2, A–C, and Supplemental Methods). This revealed a dichotomous distribution along PC1, forming distinct clusters at the extremes (Figure 2B). Immune markers related to tissue residency and scavenging functions were negatively correlated to PC1, indicating a relative reduction in STR SM (Figure 2B, left, green ellipse). Neuronal tissue and blood vessel markers (Figure 2B, pink), endothelial cells (khaki), and red blood cell proteins (Supplemental Table 2) behaved similarly. In contrast, we noted strong positive correlations between PC1 and immune markers associated with proinflammatory processes such as phagocytosis, antigen presentation, and cell recruitment (Figure 2B, right, green ellipse). Smooth muscle cell (SMC) markers were positioned almost exclusively on the PC3 positive side, with several canonical markers among the top protein correlates (Figure 2B, top, and Supplemental Figure 3A).

Several core matrisome proteins, many of which play structural or hemostatic roles (Supplemental Tables 2 and 3), negatively correlated to PC1 (Figure 2B, left, circled in orange). This included fibrillar collagens, proteoglycans, and ECM glycoproteins (Figure 2D). Conversely, a subset exhibited positive correlations with PC1 and/or PC3 (Figure 2B, right, orange ellipse), colocalized with several other fibroblast markers and demonstrated high DE in STR tissue. Pathway-level correlation further identified ECM interactions, endothelial-mesenchymal transition, and contraction as pathways highly correlated to PC3 (Supplemental Figure 3B). Overall, markers representative of the 3 hallmarks of STR tissue — inflammation, fibrosis/ECM remodeling, and muscle expansion — opposed a homeostatic protein signature and trended in the STR direction, suggesting that the 2D separation between CTRL and STR tissue in the SM layer reflects a transition from homeostatic to profibrotic states. This was further strengthened by enrichment analysis aligning with a shift from homeostasis to anabolic and inflammatory activities in STR SM (Supplemental Results 2 and Supplemental Figure 3, C–E).

### STR MP is characterized by reduced SMC markers and cellular respiration and increased immune and ECM proteins.

**Figure 3:**
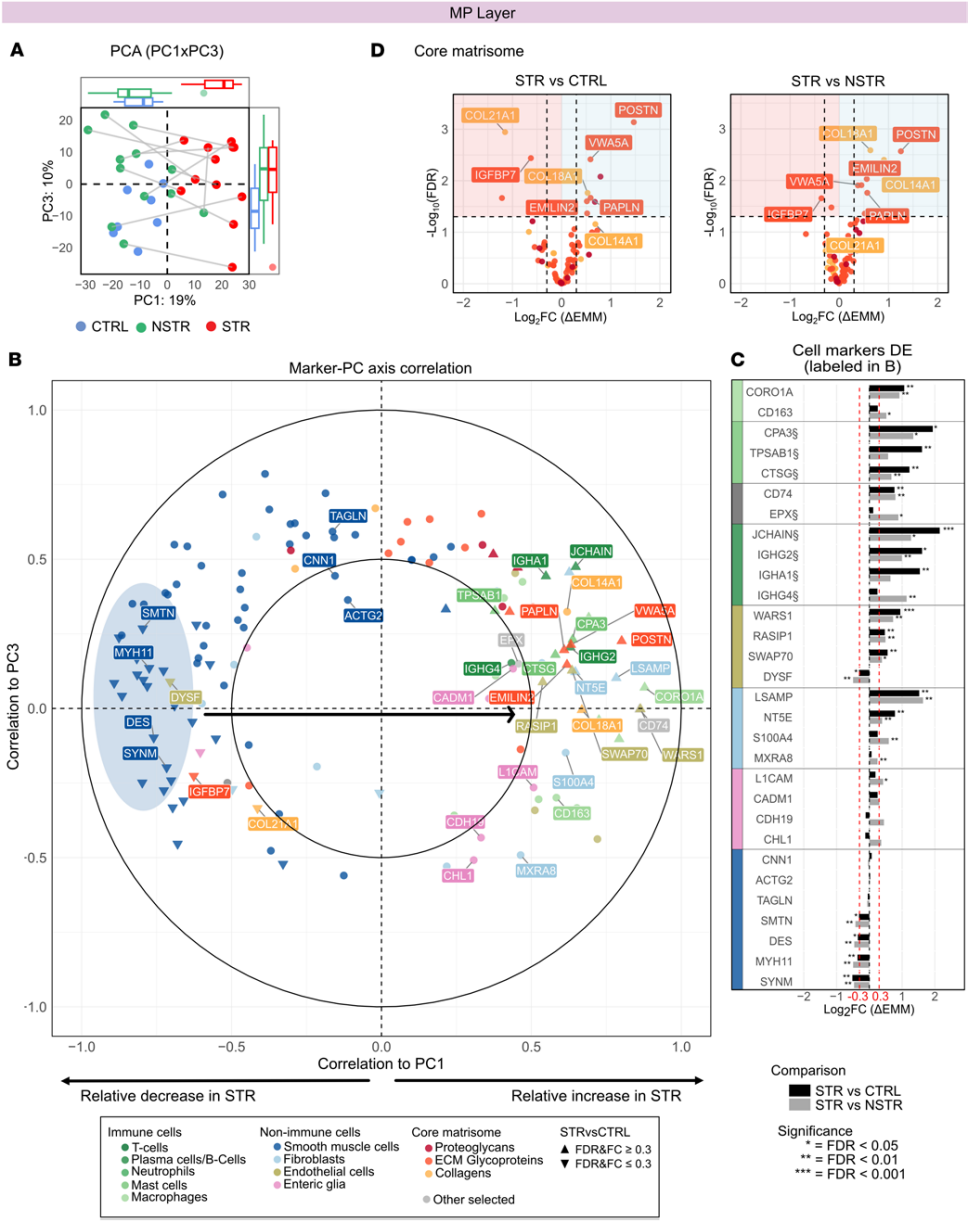
Changes in markers for SMCs, immune cells, and ECM components characterize the MP of STR tissue. (**A**) PCA plot (PC1 vs. PC3) with axis PC score box-and-whisker plots (top and right) for batch-corrected MP TMT data with complete observations. The percentage in the axis titles indicates the proportion of variance explained by each PC. Samples are color coded by tissue type, and STR and NSTR samples from the same individual are connected by lines. Axis box-and-whisker plots show the median (center line), IQR (box), whiskers extending to the smallest and largest values within 1.5 times the IQR, and outliers plotted as individual points. (**B**) Plot visualizing Pearson’s correlation coefficients between markers and PC1/PC3. Markers are color coded by assigned categories indicated below the plot with differentially expressed (DE) proteins indicated by triangles. Marker clusters and representative proteins discussed in the text are highlighted with ellipses and labels, respectively. (**C** and **D**) DE analysis results for markers from STRvCTRL and STRvNSTR comparisons within the MP layer. DE was tested using linear mixed-effects models with model-based contrasts of estimated marginal means (EMM), with degrees of freedom estimated using the Kenward-Roger method; *P* values were adjusted for multiple testing using the Benjamini-Hochberg method to control the false discovery rate (FDR). The coloring and labeling of individual proteins in **C** and **D** is consistent with **B**. (**C**) Bar plots showing DE results for the cell markers labeled in **B**, arranged by cell type annotation and colors according to the bottom of **B**. (**D**) Volcano plots of core matrisome proteins.

We next analyzed correlations between the PC axes and cell markers in the MP layer (Figure 3, A and B). First, SMC markers were distinctly positioned on the negative side of PC1, indicating a relative decrease in STR MP, including several but not all canonical SMC proteins (Figure 3B, left, dark blue markers, and Supplemental Table 4). In stark contrast, most non-SMC markers trended in the positive direction, suggesting a relative increase in STR MP (Figure 3B). This was especially evident for immune cell markers, including the top PC1-associated MHC-II protein CD74 (Supplemental Figure 4A), as well as proteins related to granules and antibodies, endothelial markers involved in angiogenesis, and fibroblast markers (Figure 3, B and C, and Supplemental Table 4). Similarly, among matrisome proteins, several ECM glycoproteins and nonfibrillar collagens positively correlated to PC1 (Figure 3, B and D). These findings suggest that STR MP is characterized by a relative reduction in SMC proteins with expansion of the non-SMC compartment, particularly immune and ECM markers; reduced cellular respiration and metabolism also accompany the increased immune activity in STR MP (Supplemental Results 3 and Supplemental Figure 4).

### Immune-, ER-, and ECM-associated proteins are predominantly increased in STR SM.

**Figure 4:**
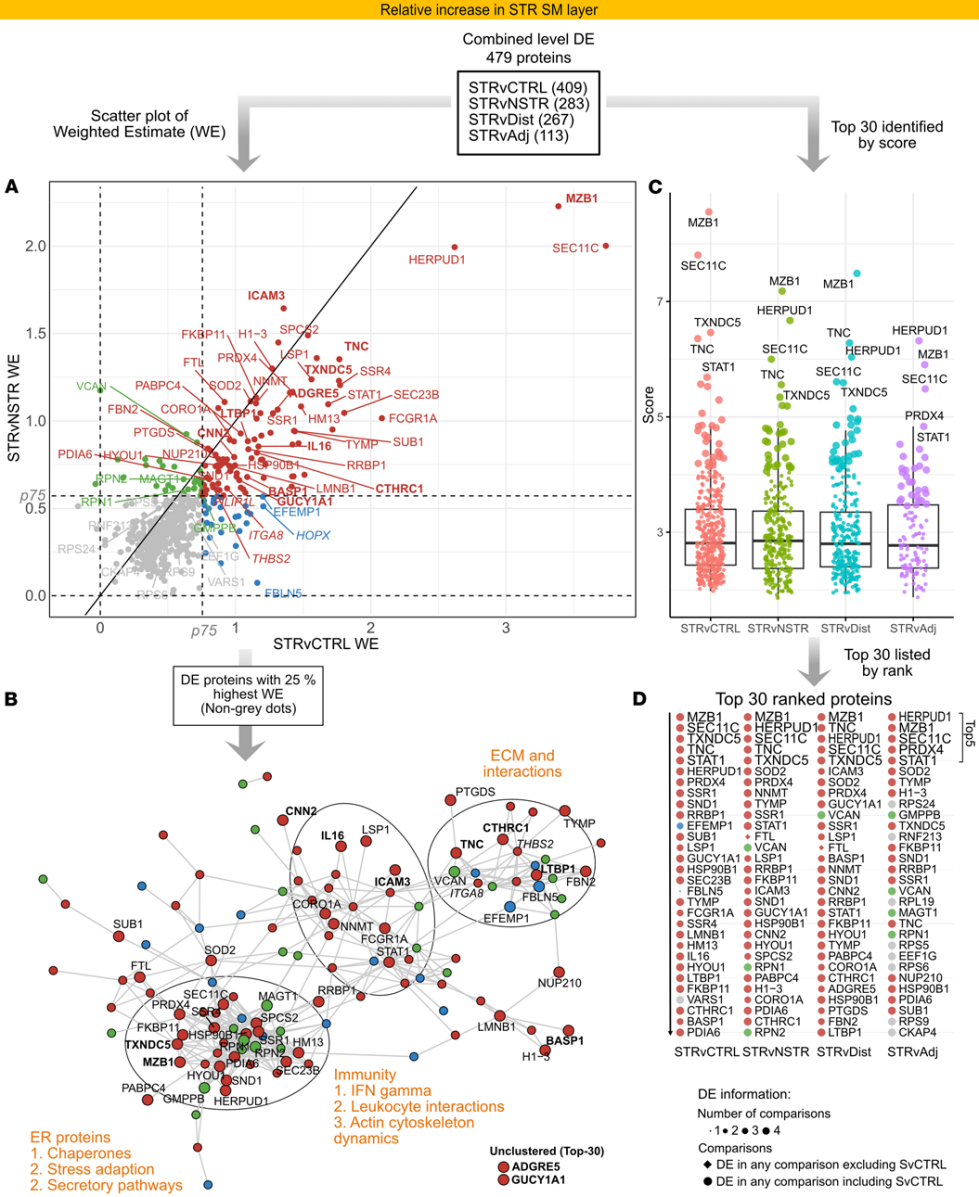
Differential protein expression in the SM reveals prominent increases in immunity-, ER-, and ECM-associated proteins in STR tissue. Combined-level DE proteins in the SM layer included 479 proteins with relative increases in STR tissue. The analysis followed a funnel-like approach starting with all DE proteins (**A**) (Supplemental Figure 8A), and then identifying themes among DE proteins with the highest fold changes (**B**) and finally identifying top-ranked proteins (**C** and **D**). (**A**) Scatter plots visualizing weighted estimate (WE, log_2_ scale) in the STRvCTRL versus STRvNSTR comparisons. The 2 inner horizontal and vertical dotted lines mark the percentiles (“p”) for each respective comparison’s WE, used for thresholding and color coding. The diagonal line (*y* = *x*) represents equal WEs in both comparisons. (**B**) STRING protein-protein interaction network of the top 25% of DE proteins with the highest deviation (WE) from STRvCTRL or STRvNSTR. Edges represent interaction scores of ≥0.4. Functional themes/keywords have been annotated to summarize the primary characteristics of these highly DE proteins. Nodes are colored according to **A**. In **A** and **B**, the top 30 proteins are labeled; those discussed more specifically are in bold. Proteins outside the top 30 but discussed in the text are in italics. (**C** and **D**) In parallel, a ranking score was calculated and used to identify top-ranked proteins. (**C**) Ranking score dot plots for DE proteins in each comparison, with the top 30 ranked proteins in larger dot size and the top 5 labeled. In **C**, note that the scoring incorporates directionality; proteins with stronger positive changes receive more positive scores. (**D**) The top 30 DE proteins in ranked order, with the top 5 shown in larger font, providing details about DE comparisons (symbol size, shape) and relation to thresholds in **A** (color).

To enhance analysis robustness, we leveraged our data from parallel TMT-MS and TIMS-TOF-MS and used integrated DE results at the combined level (Supplemental Methods) for comparisons from here forward. In addition, as the initial PCA (Figure 2A) and a refined PCA with NSTR tissue subdivided into tissue adjacent to (Adj) or more distal from (Dist) the stricture (Supplemental Figure 5, Supplemental Figure 1D, and Supplemental Methods) showed progressive trends along PC1, analysis using integrated data was complemented by STRvAdj and STRvDist comparisons (Supplemental Results 4 and Supplemental Figures 6 and 7). Assessment of the 479 DE proteins relatively increased in STR SM at the combined level (Supplemental Figure 6 and Supplemental Figure 8A) showed the STRvCTRL magnitude of change (expressed as weighted estimate [WE], log_2_ scale; Supplemental Methods) generally exhibited higher WEs than STRvNSTR (Figure 4A), although values were well correlated across most proteins. Functional grouping of proteins above the upper quartile of WEs revealed 3 prominent functional domains as key themes among the highest DE proteins: ER, immunity, and ECM (Figure 4B), with a considerable amount of these proteins being secreted or adhesion proteins (Supplemental Table 5). Ranking the DE SM proteins (Supplemental Methods) identified the top 30 (Figure 4, C and D) which, including the very top 5, were distributed across the 3 major functional clusters identified (Figure 4B). ER-associated proteins were prominent among the top 30 (Figure 4, B–D) and displayed distinctly higher WEs than, for instance, ribosomal proteins. High WEs were particularly notable for a subset of ER proteins in the top 5: MZB1, HERPUD1, TXNDC5, and PRDX4 (Figure 4, B–D). These have chaperone-like activity or are involved in stress adaptation (Supplemental Table 6).

Among immune-related DE proteins in STR SM (Figure 4, B–D), top proteins included the transcription factor STAT1, phagocytosis-related proteins, and key proteins of leukocyte migration and recruitment (Figure 4, B–D, and Supplemental Table 6). Strikingly, IL-16, a matrisome-associated cytokine, was among the top DE proteins in STR SM, along with the leukocyte adhesion proteins ICAM3 and ADGRE5.

The next major group of DE proteins in STR SM were ECM proteins and included the glycoproteins TNC, CTHRC1, and LTBP1. These are directly or indirectly involved in tissue regeneration and fibrosis progression (Figure 4B and Supplemental Table 6) and formed a top subset of highly DE, nonstructural ECM proteins characterized by receptor-binding activity. Indeed, a notable portion of the top-ranked DE proteins were identified as secreted or adhesion proteins mediating ECM-cell or cell-cell interactions (Supplemental Table 5). Interestingly, ITGA8, which binds TNC and other ECM proteins, was the only nonleukocyte integrin receptor significantly increased at the combined level. ITGA8 was outside the top 30 but within the top 25% of WEs and was the most DE integrin in STR SM. Correlation analysis (Supplemental Results 5 and Supplemental Figure 9, A–C) revealed some top-ranked DE proteins were characterized by strong correlation with ECM proteins (Supplemental Figure 9, C and G, and Supplemental Table 7), including GUCY1A1, a marker of specific fibroblast subsets in various organs (26) (Supplemental Table 6), CNN2, and BASP1. This analysis also highlighted clustering of top DE immune and ER proteins, including IL-16, MZB1, and TXNDC5 and the ECM glycoprotein CTHRC1. Overall, proteins associated with 3 functional categories — immune response, ER, and ECM — showed the most pronounced increases among the 479 proteins increased in STR SM.

### DE proteins decreased in STR SM are associated with vascular structures and lipid metabolism.

**Figure 5:**
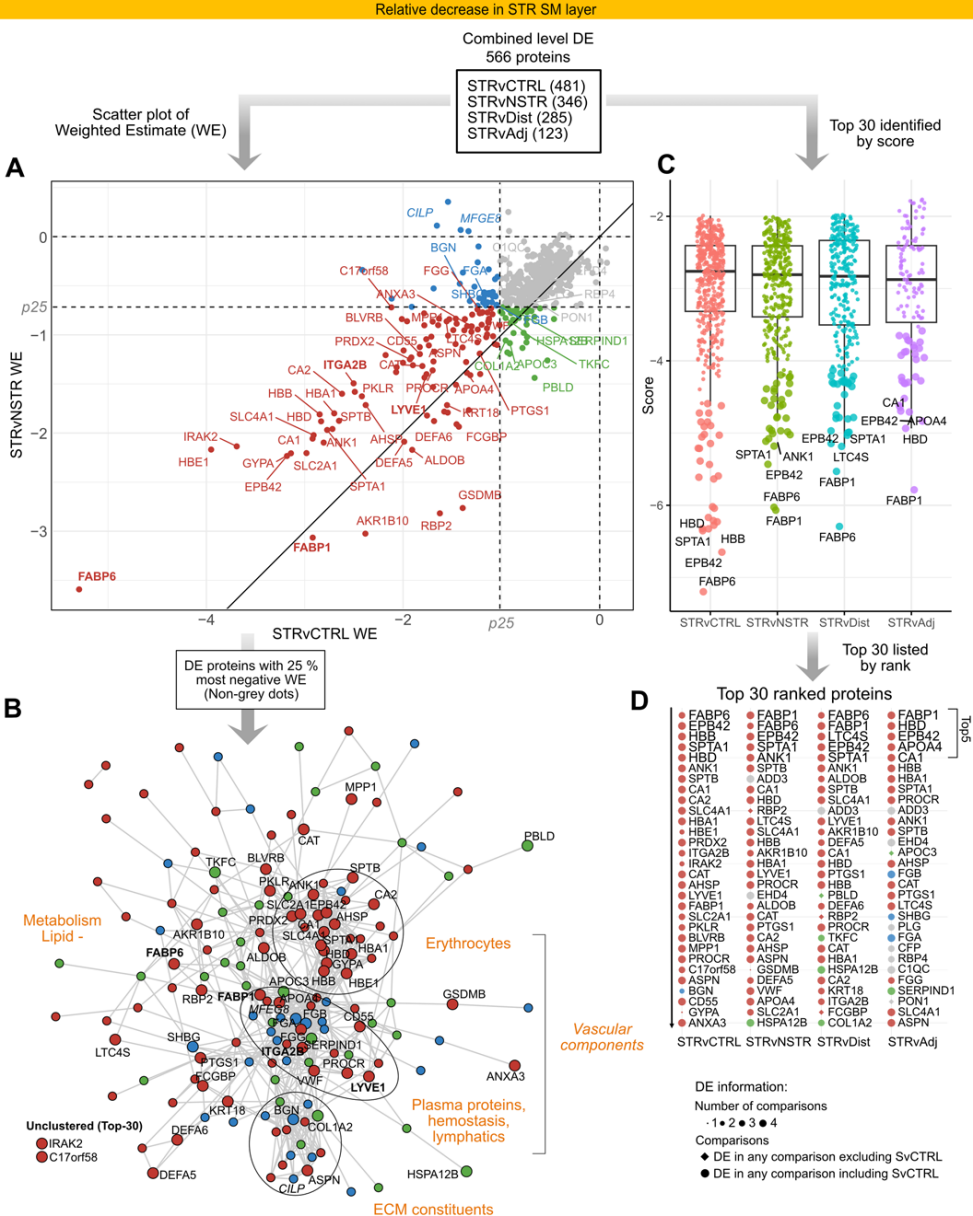
DE proteins associated with vascular structures and lipid metabolism show a marked decrease in the STR SM. The analysis followed a funnel-like approach starting with all DE proteins (**A**) (Supplemental Figure 8B), and then identifying themes among DE proteins with the highest fold changes (**B**) and finally identifying top-ranked proteins (**C** and **D**). (**A**) Scatter plots visualizing weighted estimate (WE, log_2_ scale) in the STRvCTRL versus STRvNSTR comparisons. The 2 inner horizontal and vertical dotted lines mark the percentiles (“p”) for each respective comparison’s WE, used for thresholding and color coding. The diagonal line (*y* = *x*) represents equal WEs in both comparisons. (**B**) STRING protein-protein interaction network of the top 25% of DE proteins with the highest deviation (WE) from STRvCTRL or STRvNSTR. Edges represent interaction scores of ≥0.4. Functional themes/keywords have been annotated to summarize the primary characteristics of these highly DE proteins. Nodes are colored according to **A**. In **A** and **B**, the top 30 proteins are labeled; those discussed more specifically are in bold. Proteins outside the top 30 but discussed in the text are in italics. (**C** and **D**) In parallel, a ranking score was calculated and used to identify top-ranked proteins. (**C**) Ranking score dot plots for DE proteins in each comparison, with the top 30 ranked proteins in larger dot size and the top 5 labeled. Note that the scoring incorporates directionality; proteins with stronger negative changes receive more negative scores. (**D**) The top 30 negative DE proteins in ranked order, with the top 5 shown in larger font, providing details about DE comparisons (symbol size, shape) and relation to thresholds in **A** (color).

Prominent themes among the 566 DE proteins with a relative decrease in STR SM (Supplemental Figure 6 and Supplemental Figure 8B), particularly among those with the most negative estimates (Figure 5A, non-gray dots), include blood and plasma proteins, ECM constituents, and metabolism (Figure 5B). Erythrocyte proteins formed a large, dense cluster of top 30 DE proteins decreased in SM (Figure 5, B–D, and Supplemental Table 8). Other top blood- or vasculature-related reduced DE proteins included the key platelet integrin ITGA2B and the lymphatic endothelium receptor LYVE1 (Figure 5, B–D, and Supplemental Table 8). Proteins associated with endothelial hemostasis, detoxification processes, and lipid or leukotriene metabolism were also among the top 30 decreased proteins (Supplemental Table 8). The relative reduction in proteins associated with vasculature or homeostatic structural elements in STR SM is consistent with PCA (Figure 2) and enrichment analysis (Supplemental Results 2, and Supplemental Figure 3, C and D). Together, the data indicate structural remodeling of STR SM, with vascular remodeling as a dominant feature.

### Changes in immune processes and protein handling characterize proteins relatively increased in STR MP.

**Figure 6:**
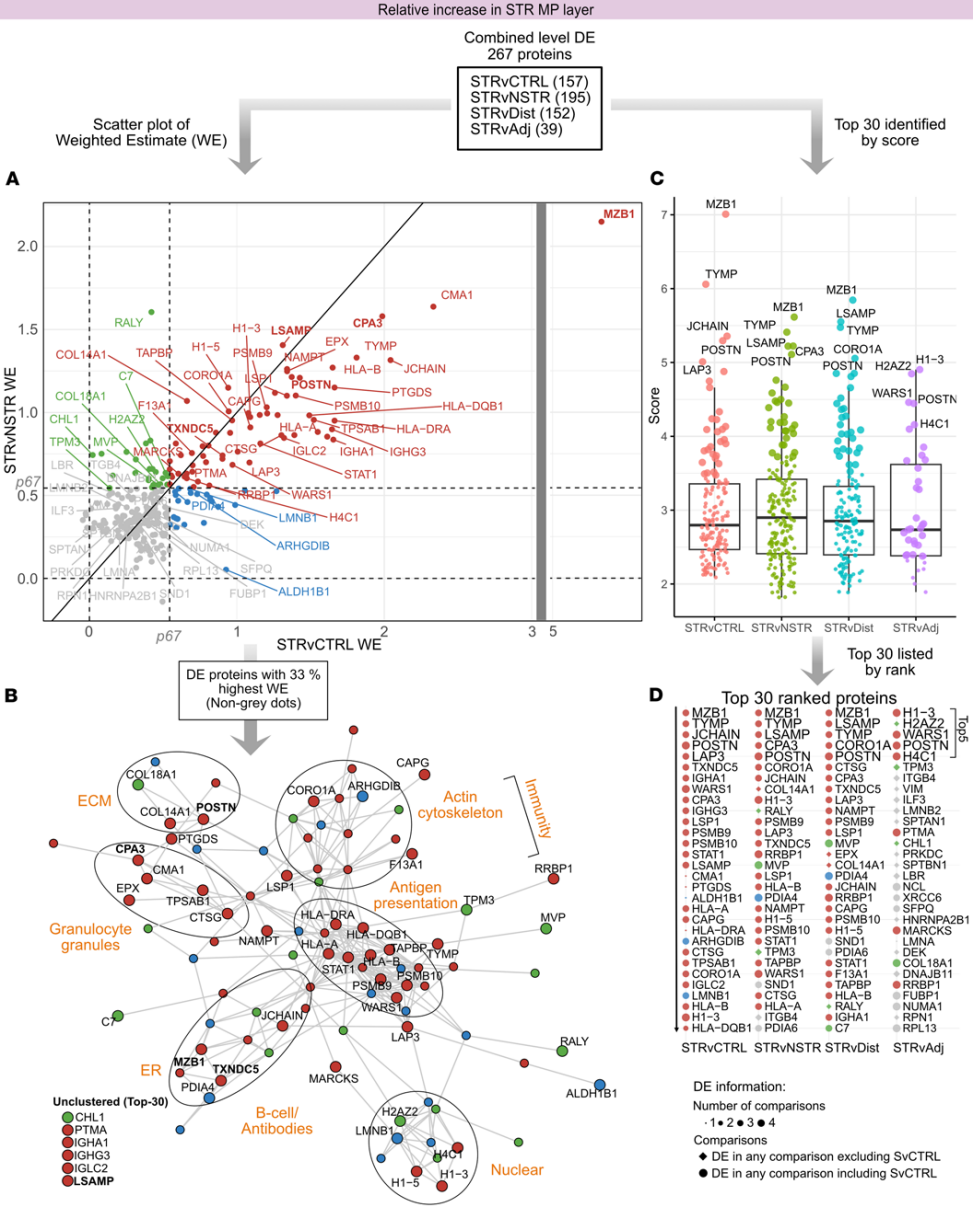
Proteins associated with immune processes, ECM, and protein handling display relative increases in STR MP. The analysis followed a funnel-like approach starting with all DE proteins (**A**) (Supplemental Figure 8C), and then identifying themes among DE proteins with the highest fold changes (**B**) and finally identifying top-ranked proteins (**C** and **D**). (**A**) Scatter plots visualizing weighted estimate (WE, log_2_ scale) in the STRvCTRL versus STRvNSTR comparisons. The 2 inner horizontal and vertical dotted lines mark the percentiles (“p”) for each respective comparison’s WE, used for thresholding and color coding. The diagonal line (*y* = *x*) represents equal WEs in both comparisons. Note that there is a break in the *x* axis marked with a gray vertical bar so that proteins with a very large WE can be visualized. (**B**) STRING protein-protein interaction network of the top 33% of DE proteins with the highest deviation (WE) from STRvCTRL or STRvNSTR. Edges represent interaction scores of ≥0.4. Functional themes/keywords have been annotated to summarize the primary characteristics of these highly DE proteins. Nodes are colored according to **A**. In **A** and **B**, the top 30 proteins are labeled; those discussed more specifically are in bold. (**C** and **D**) In parallel, a ranking score was calculated and used to identify top-ranked proteins. (**C**) Ranking score dot plots for DE proteins in each comparison, with the top 30 ranked proteins in larger dot size and the top 5 labeled. In **C**, note that the scoring incorporates directionality; proteins with stronger positive changes receive more positive scores. (**D**) The top 30 DE proteins in ranked order, with the top 5 shown in larger font, providing details about DE comparisons (symbol size, shape) and relation to thresholds in **A** (color).

Features related to immunity, actin cytoskeleton, ER, and ECM characterized the subset of the 267 DE proteins (Supplemental Figure 7 and Supplemental Figure 8C) with the largest deviations from control tissues (Figure 6, A and B, and Supplemental Table 10) in STR MP. In particular, DE proteins associated with immune function were strongly represented among the top increased proteins in STR MP (Figure 6, C and D). These include proteins related to B cells and antibodies, granulocyte granules, antigen presentation, phagocytosis, and leukocyte migration (Figure 6B and Supplemental Table 10). Among granule proteins (Figure 6, A and B, and Supplemental Table 10), the secreted mast cell protein CPA3 was among the very top DE in several comparisons (Figure 6, C and D). Notably, the fold change in MZB1, which is associated with B cells and has ER chaperone function, was exceptionally high in both STR MP and SM (Figure 4A and Figure 6A). Similar to MZB1, another ER protein with chaperone function, TXNDC5, was also among the very top DE proteins in STR SM (Figure 4, C and D).

Among ECM proteins, the matricellular glycoprotein POSTN, involved in ECM remodeling and signaling dynamics, stood out. Notably, POSTN was the only protein among the top 5 in all group comparisons (Figure 6, B–D, and Supplemental Table 10). The triad of POSTN, MZB1, and granule proteins constituted the very top DE proteins increased in STR MP (Supplemental Table 5). Extended analysis also highlighted correlation between POSTN, mast cell granules, and key intracellular proteins (Supplemental Results 6 and Supplemental Figure 9, D–G). Adhesion proteins were also highly DE and were mainly related to leukocyte or neuronal adhesion, with LSAMP being the most notable (Figure 6, C and D, and Supplemental Tables 10 and 11). To summarize, immune-related proteins were prominent among those showing the greatest relative increase in STR MP. Notably, many of these are secreted and include granule proteins derived from mast cells and eosinophils, as well as the matricellular glycoprotein POSTN.

### Proteins associated with redox balance, lipid metabolism, and muscle function are decreased in STR MP.

**Figure 7:**
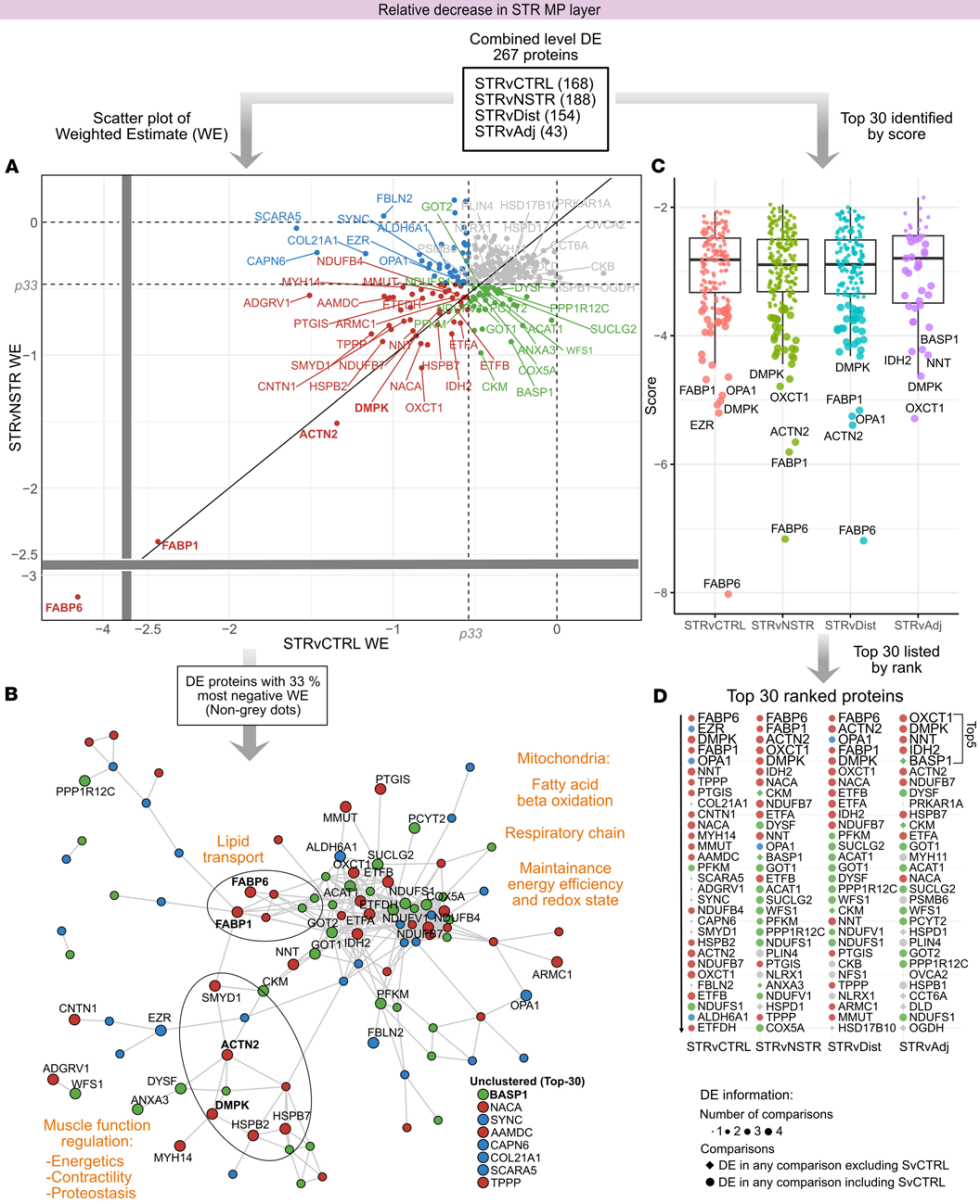
DE analysis reveals proteins associated with redox balance, lipid metabolism, and muscle function are reduced in STR MP. The analysis followed a funnel-like approach starting with all DE proteins (**A**) (Supplemental Figure 8D), and then identifying themes among DE proteins with the highest fold changes (**B**) and finally identifying top-ranked proteins (**C** and **D**). (**A**) Scatter plots visualizing weighted estimate (WE, log_2_ scale) in the STRvCTRL versus STRvNSTR comparisons. The 2 inner horizontal and vertical dotted lines mark the percentiles (“p”) for each respective comparison’s WE, used for thresholding and color coding. The diagonal line (*y* = *x*) represents equal WEs in both comparisons. Note that there are breaks in the *x* and *y* axes marked with a gray horizontal and vertical bar, respectively, so proteins with a very large WE can be visualized. (**B**) STRING protein-protein interaction network of the top 33% of DE proteins with the highest deviation (WE) from STRvCTRL or STRvNSTR. Edges represent interaction scores of ≥0.4. Functional themes/keywords have been annotated to summarize the primary characteristics of these highly DE proteins. Nodes are colored according to **A**. In **A** and **B**, the top 30 proteins are labeled; those discussed more specifically are in bold. (**C** and **D**) In parallel, a ranking score was calculated and used to identify top-ranked proteins. (**C**) Ranking score dot plots for DE proteins in each comparison, with the top 30 ranked proteins in larger dot size and the top 5 labeled. In **C**, note that the scoring incorporates directionality; proteins with stronger negative changes receive more negative scores. (**D**) The top 30 negative DE proteins in ranked order, with the top 5 shown in larger font, providing details about DE comparisons (symbol size, shape) and relation to thresholds in **A** (color).

Assessing the 267 DE proteins relatively reduced in STR MP (Supplemental Figure 7 and Supplemental Figure 8D) revealed that mitochondrial, metabolic, and SMC proteins were among those with the most negative WE (Figure 7A, non-gray, and Figure 7B) and among the top 30 DE proteins (Figure 7, C and D), consistent with the complete network (Supplemental Figure 8D). We identified subsets of these protein categories with markedly greater fold decreases than the other DE proteins (Figure 7A and Supplemental Table 12). For example, the top mitochondrial proteins decreased in STR MP included key proteins involved in ketone metabolism and maintenance of redox balance and respiratory chain function in mitochondria (Supplemental Table 12). Proteins related to lipid transport and fatty acid β-oxidation (Figure 7B and Supplemental Table 12) were another group of highly decreased proteins. Notably, the lipid transporters FABP6 and FABP1 displayed by far the largest decreases among all relatively decreased proteins in STR MP (Figure 7A, lower left). This is similar to the SM layer (Figure 5A). Lastly, a group of muscle-associated proteins (Figure 7B and Supplemental Table 12) showed substantially greater fold decreases compared with other DE proteins, particularly compared with structural SMC proteins. Among these, DMPK and ACTN2, which play key regulatory roles in muscle cells, were among the most highly decreased proteins in STR MP (Figure 7, C and D, and Supplemental Table 12). Taken together, the results show that cellular respiration and metabolic pathways constituted the majority of DE proteins reduced in STR MP, with key proteins in oxidative stress protection, lipid handling, and muscle cell function among those with the most prominent reductions.

### Interlayer analysis highlights shared and distinct proteome changes in SM and MP.

**Figure 8:**
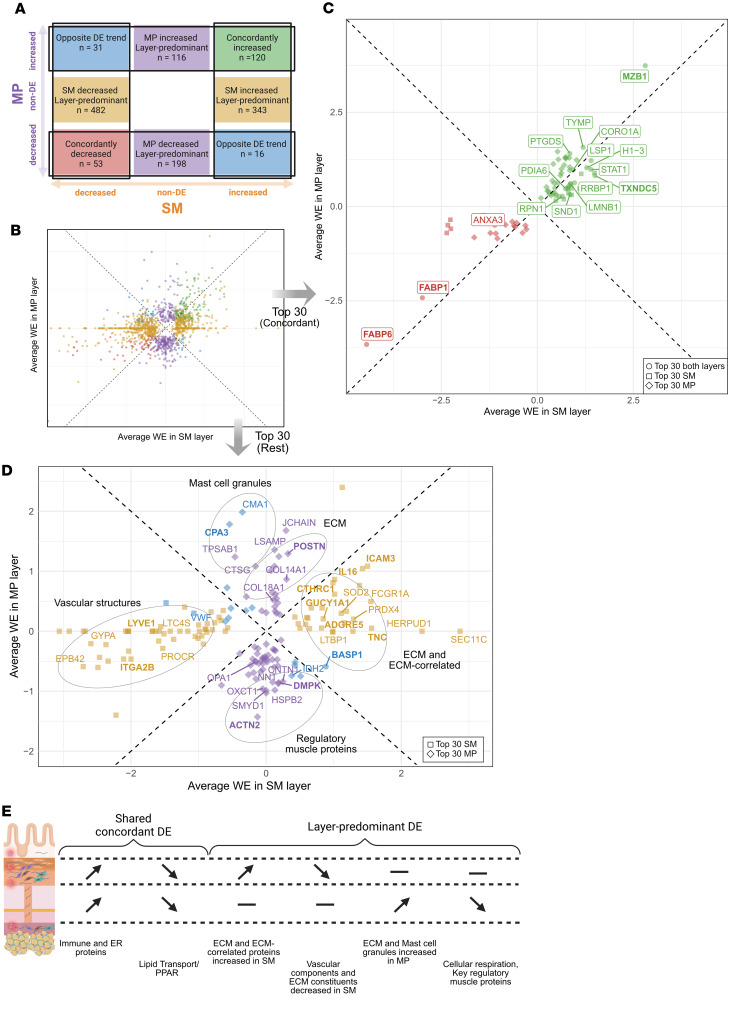
Interlayer analysis of top-ranked DE proteins in the SM and MP layers reveals shared and layer-dominant features. (**A** and **B**) DE proteins in the SM and MP layers were categorized as concordant, discordant, or layer-predominant DE (**A**). The scatter plot (**B**) illustrates the magnitude of change in SM versus MP for all 1,359 DE proteins, using average weighted estimates (WE, log_2_ scale) from the STRvCTRL and STRvNSTR comparisons as proxies for the relative change in each layer. For proteins not quantified in 1 layer, the average WE was set to zero. Dots are color coded according to **A**. (**C** and **D**) Further assessment focused on the 228 proteins in the union of the top 30 DE proteins of each layer and direction (“Top 30”). (**C**) Three proteins (MZB1, FABP1, FABP6) fall outside the displayed axis range in **B**. Note that Top 30 within each layer and DE direction denotes proteins ranked among the Top 30 in any of the 4 comparisons; as rankings differ in the 4 comparisons, the Top 30 contains more than 30 proteins (see SM, Figure 4D and Figure 5D; MP, Figure 6D and Figure 7D). The scatter plots show the magnitude of change in SM versus MP as in **B** but limited to the Top 30 proteins mapped to the concordant (**C**) or the layer-predominant/discordant category (**D**). Dots are color coded according to **A**. Symbols indicate in which layer they were Top 30 (squares, SM; diamonds, MP; circles, both layers). In **C**, concordantly expressed proteins within the Top 30 of both layers are labeled. In **D**, selected top proteins discussed in Figures 4–7 are labeled. In **C** and **D**, proteins discussed in the text are in bold. In **D**, ellipses refer to protein categories discussed in the text. (**E**) A summary of the protein categories showing shared concordant or layer-predominant DE.

Having identified interrelated DE proteins (Supplemental Results 5 and 6), indicating related processes occurring within each layer, we extended the separate analyses of the SM and MP to explore shared proteomic signatures across layers in STR tissue. DE proteins from each layer were thus cross-tabulated by their direction of change, enabling identification of proteins with concordant positive or negative trends, opposing trends, or layer-predominant changes (Figure 8, A and B). Based on the functional categories of the proteins with shared DE across layers (Supplemental Results 7 and Supplemental Figure 10), particularly concordantly increased proteins involved in immunity and mRNA, nucleus, and protein handling processes (Supplemental Figure 10A), we focused on the union of top 30 DE proteins in each layer and direction and assessed their expression across layers (Figure 8, C and D).

Among the top 30 DE proteins within the concordant categories, 16 DE proteins ranked among the highest in both layers (Figure 8C and Supplemental Table 13), suggesting prominent expression shared across the layers. Concordantly increased proteins included ER chaperones (MZB1 and TXNDC5), which had the largest WEs in both layers (Supplemental Figure 10, A and B), immune proteins (STAT1, CORO1A, and LSP1), muscle contraction/relaxation (PTGDS), and angiogenesis-related proteins (TYMP) (Figure 8C, green). In contrast, the lipid transporters FABP1 and FABP6 were strongly concordantly decreased (Figure 8C, red).

The remaining top 30 proteins displayed layer-predominant DE (Figure 8D, MP in purple and SM in yellow) or displayed direction of the DE that was exclusive to one layer (Figure 8D, blue). Proteins with such layer-predominant DE included increased ECM-associated proteins and reduced vascular structural proteins in STR SM. In contrast, they included increased ECM and mast cell granules accompanied by decreased regulatory muscle proteins in STR MP (Figure 8, D–F). The findings highlight both shared and distinct proteome changes in SM and MP, emphasizing the importance of layer-resolved analysis.

### Progressive trends of DE proteins in STR SM suggest a continuum of profibrotic tissue remodeling as proximity to the stricture increases.

We hypothesized that strictures likely develop along a continuum in the ileum, rather than being demarcated by a sharp border between NSTR and STR tissue. As such, NSTR tissue would progressively shift, in a gradient fashion, toward an STR phenotype as proximity to the stricture increased. As mentioned above, refined PCA of STR SM with paired NSTR subdivided into Adj and Dist, which differ in proximity to the stricture (Supplemental Figure 1, D and E), revealed progressive increase in PC1 scores from CTRL→Dist→Adj→STR (Supplemental Figure 5, box plot *x* axis), consistent with a gradual transition toward STR. In addition, increases in PC3 scores in SM were observed in Adj relative to CTRL and Dist (Supplemental Figure 5, box plot *y* axis).

**Figure 9:**
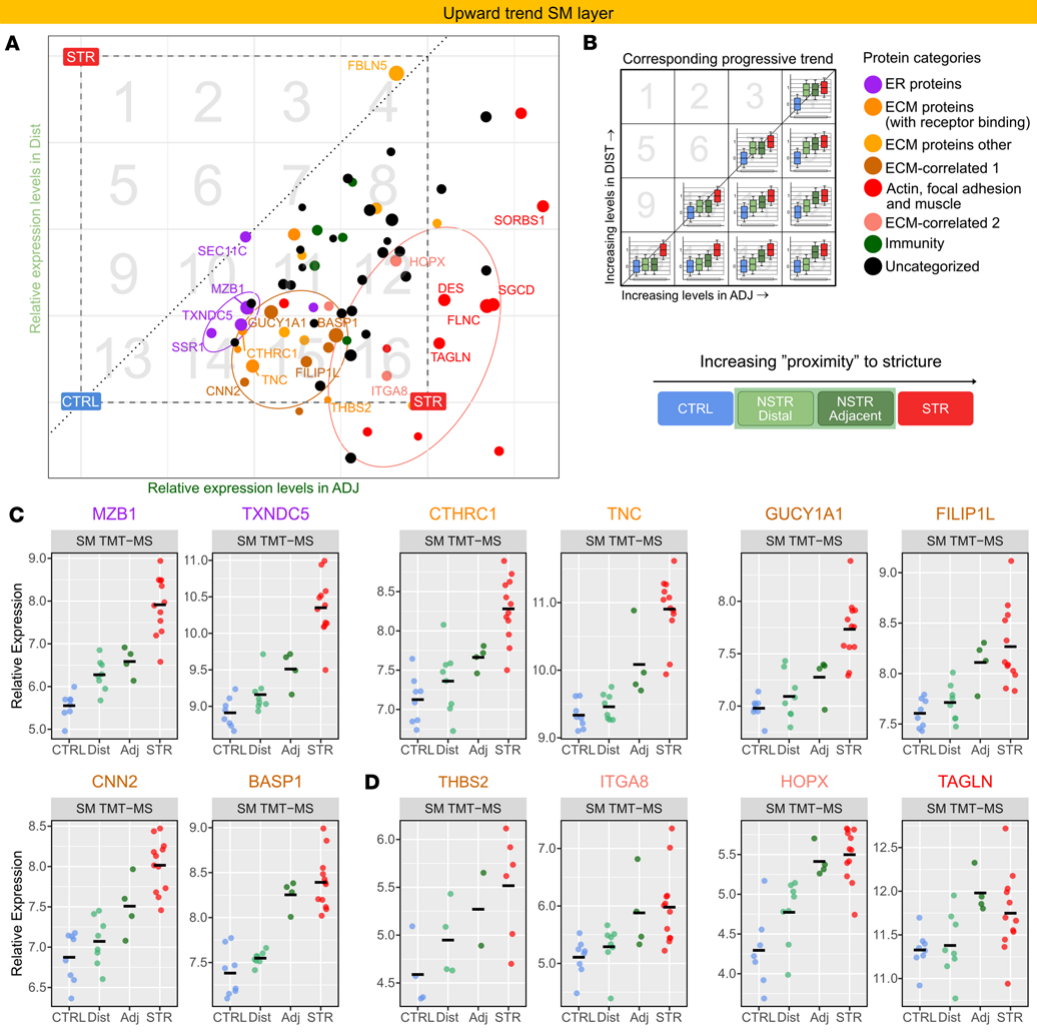
ER-, ECM-, and muscle-associated proteins are characterized by progressively increased expression with proximity to STR tissue. The figure shows subsets of combined-level DE proteins in the SM layer that progressively increase (70 proteins; **A**–**D**) with proximity to the stricture (Supplemental Methods 10, Trend analysis). (**A**) Scatter plots showing the expression level in Dist (*y* axis) and Adj (*x* axis) relative to the CTRL–STR range (CTRL = 0; STR = 1; dashed square). Due to this scaling, any dot (protein) in the coordinate system will correspond to a unique trend: CTRL = 0, Dist = *y*, Adj = *x*, STR = 1. The dashed square is divided into 16 numbered boxes to facilitate reference of each protein to the corresponding box in **B** to approximate a protein’s expression pattern based on its coordinates. The proteins (dots) are colored by category as in **B**. Dot size reflects the –log_10_(merged *P* value) from the AdjvCTRL comparison and serves as an indicator of the separation of Adj from CTRL. Ellipses highlight proteins with notable progressive DE patterns, including top-ranked ER proteins (violet), ECM-associated proteins (brown), and a separate group of ECM- and muscle-associated proteins (pink ellipse). (**C** and **D**) Dot plots of normalized abundance (log_2_ scale), adjusted for TMT set, from SM TMT-MS data across refined tissue types for selected labeled proteins in **A**. Horizontal bars mark the mean. Protein names are colored according to protein category in **A** and **B**. Dot color corresponds to the proximity key below the plots. Corresponding plots from the TIMS-TOF-MS data are shown in Supplemental Figure 11 as validation. See Supplemental Figure 11 for details.

**Figure 10:**
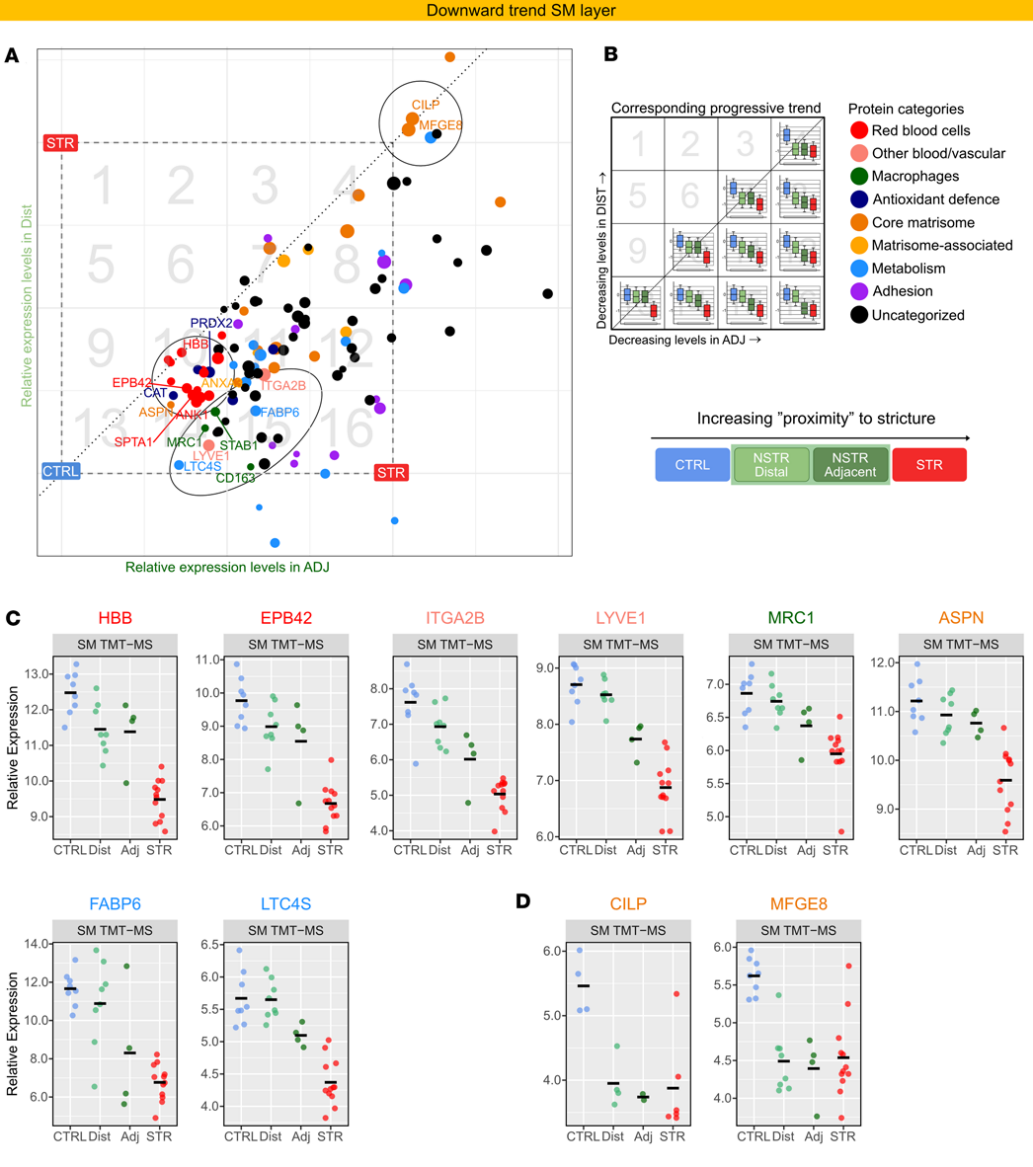
Proteins linked to functional and structural homeostasis display progressively decreased expression with proximity to STR tissue. (**A**) Scatter plot showing the expression level in Dist (*y* axis) and Adj (*x* axis) relative to the CTRL–STR range (CTRL = 0; STR = –1; dashed square). Due to this scaling, any dot (protein) in the coordinate system will correspond to a unique trend: CTRL = 0, Dist = *y*, Adj = *x*, STR = –1. The proteins (dots) are colored by category as in **B**. (**B**) The dashed square is divided into 16 numbered boxes to facilitate reference of each protein to the corresponding box to approximate a protein’s expression pattern based on its coordinates. Dot size reflects the –log_10_(merged *P* value) from the AdjvCTRL comparison and serves as an indicator of the separation of Adj from CTRL. Ellipses highlight proteins with notable progressive DE patterns discussed in the text. The circle in the upper right corner highlights 2 proteins with reported antifibrotic activity, which are also displayed in **D**. (**C** and **D**) Dot plots of normalized abundance (log_2_ scale), adjusted for TMT set, from SM TMT-MS data across refined tissue types for selected labeled proteins in **A**. Horizontal bars mark the mean. Protein names are colored according to protein category in **A** and **B**. Dot color corresponds to the proximity key below the plots. Corresponding plots from the TIMS-TOF-MS data are shown in Supplemental Figure 11 as validation. See Supplemental Figure 11 for details.

Building on these observations, we identified DE proteins with expression profiles consistent with a CTRL-to-STR progression (Supplemental Methods). Scaling the relative expression in Dist and Adj within the CTRL–STR range enabled visualization of DE proteins sharing similar trends (Figure 9, A and B, Figure 10, A and B, and Supplemental Figure 11, A, B, E, and F). DE proteins elevated in STR tissue linked to ECM (Supplemental Figure 9, C and G, and Supplemental Tables 6 and 7) were prominently enriched in patterns showing progressive increases from CTRL to STR in SM (Figure 9, A–D, orange; boxes 11 and 15 in B). Several ER proteins had a similar trend (Figure 9, A–D, violet; boxes 10 and 14 in B). As expected, a protein’s relative position within the CTRL–STR range correlated with the number of sequential comparisons in which it was DE (Supplemental Figure 11, A and B). Eight proteins were significantly increased in both CTRL-to-Adj and Adj-to-STR comparisons (Supplemental Figure 11, A and B, violet), including top-ranked ER (TXNDC5 and MZB1), ECM (TNC and CTHRC1), and myofibroblast-associated (GUCY1A1) proteins discussed earlier. Consistent with PC3 results (Supplemental Figure 3, A and B, and Supplemental Figure 5), proteins involved in actin dynamics, focal adhesion, and SMC function showed similar expression in Adj and STR (Figure 9A, red within pink ellipse). Interestingly, a subset of ECM-correlated proteins (HOPX, ITGA8, and THBS2; Supplemental Table 9) mirrored this pattern, suggesting an association with muscle (pink dots within pink ellipse).

We also addressed DE proteins decreased in STR SM. In contrast with the above findings, many ECM components, macrophage proteins, and top DE proteins associated with vasculature (erythrocytes, platelets, lymph endothelial), lipids, and antioxidant defense showed progressive decreasing trends from CTRL to STR (Figure 10, A–D). A few proteins (Figure 10A, upper right circle), notably 2 with reported antifibrotic function (CILP and MFGE8), display similarly low relative levels as in STR (Figure 10D). Together, the observed trends indicate a progressive profibrotic remodeling as proximity to STR tissue increases, with relative enrichment of smooth muscle and other contractile cells, such as myofibroblasts, in tissue adjacent to the stricture.

## Discussion

Using unbiased proteomic analysis on LMD tissue layers from intestinal strictures, we provide the first report to our knowledge of proteome changes in the SM and MP layers that are most profoundly altered in CD-associated strictures. Prominent increases in ECM proteins were apparent in both layers, but with unique profiles, suggesting that ECM remodeling is spatially compartmentalized and emphasizing the value of a layer-wise approach. This insight into ECM changes in biologically distinct tissue layers complements recently reported matrisome (27) profiling of decellularized full-thickness STR tissue (16) and is particularly relevant given the increasing attention to the ECM as an active component of disease progression (28). Indeed, several top ECM proteins DE in STR SM observed here, such as CTHRC1, TNC, and LTBP1, are reported myofibroblast (29) or pathogenic fibroblast markers (21, 29, 30) with putative or established roles in fibrosis (28, 29, 31–33). CTHRC1 is implicated in multiple profibrotic pathways (34) and CTHRC1^+^ pathogenic fibroblast subsets are key ECM producers in several organs, including the intestine (21, 29, 30). Moreover, the coordinated increase in CTHRC1 with highly DE immune-related proteins, including the matrisome-associated cytokine IL-16 (35–37), raises possible immune-ECM communication pathways in STR SM. Elevated IL-16 has been reported in CD colon (38, 39), and its association with CTHRC1 and possible role in ileal strictures warrants further investigation. Moreover, CTHRC1 clustering with TXNDC5, a proposed antifibrotic target due to its involvement in TGF-β responses and folding of fibrogenic proteins (40), identifies an interaction network whose disruption may be exploited for therapeutic approaches.

Our finding of TNC as the most highly increased ECM protein in STR SM extends the recent report of TNC as a matrix protein produced by submucosal myofibroblasts derived from patients with inflammatory bowel disease (IBD) cultured in vitro (41). While TNC binds integrins (42), including ITGA8, the most increased DE integrin in STR SM identified here, it can also function as a DAMP (43), activating fibroblasts through TLR4 signaling (44, 45). We also refine the recent observation of the ECM protein LTBP1, a reservoir for latent TGF-β, as a top protein increased in full-thickness fibrostenotic intestine (16) to being increased in STR SM. Furthermore, TNC and LTBP1 were highly intercorrelated with other DE proteins, including ECM proteins, the fibroblast marker GUCY1A1 (26), the antiangiogenetic protein FILIP1L, and adhesion proteins ICAM3 and ADGRE5. Overall, the observed increase in the triad of ECM proteins CTHRC1, TNC, and LTBP1, and their distinct correlation with immune, ER/chaperone, and ECM proteins, suggests their possible role in distinct yet parallel processes in stricture progression in the SM.

A marked decrease in proteins associated with structural and functional homeostasis, particularly vascular components, was also a layer-predominant feature of STR SM. This suggests vascular remodeling, potentially impairing local oxygenation and lymphatic flow. Reduced vessel density and hypoxia are hallmarks of fibrotic remodeling and may further drive profibrotic processes (29). STR SM also showed decreases in ECM proteins, some of which have protective roles, including CILP and MFGE8 with reported antifibrotic properties. The similar reduction of these proteins in Dist, Adj, and STR groups relative to CTRL raises the possibility that their reduction could be an early event in stricture pathogenesis. However, longitudinal studies are needed to address this possibility. Although our observed reduction of MFGE8 in STR SM differs from its reported increase in full-thickness fibrostenotic intestine (16), which was localized to the epithelium (16), the data collectively suggest that a compartment-specific change in MFGE8 characterizes CD-associated STR tissue and merits further study.

Layer-predominant proteins increased in STR MP included the ECM protein POSTN (29, 46, 47) and mast cell granules, the latter being consistent with reports of mast cell accumulation in STR MP (48). While POSTN is implicated in cardiac hypertrophy (46) and vascular SMC migration (49), mast cell degranulation products affect tissue remodeling and muscle expansion in the airways (50–52). Whether CPA3 — the most highly DE mast cell granule protein in our data — influences MP expansion in CD strictures remains to be investigated. Additional top DE proteins in STR MP were related to general immune processes or specific immune cells such as B cell–associated proteins and eosinophil peroxidase. These data suggest heightened immune activity in STR MP and are consistent with reports of increased immune cell populations, including B cells, IgG^+^ plasma cells (53–55), and activated eosinophils (20) in deeper layers of fibrostenotic intestine. Although any causal or secondary relationship of these cells or their products to stricturing remains to be experimentally addressed, released mediators, proteases, and ECM proteins identified in this study could possibly interact with SMCs and contribute to MP hypertrophy.

In stark contrast to the relative enrichment of immune and ECM proteins in STR MP, SMC markers were strikingly reduced, indicating their relative decrease in STR MP. Given that muscle hypertrophy is a histopathological feature of CD strictures (22), this reduction may appear counterintuitive. However, pathologic remodeling may alter homeostatic cell composition through infiltration and ECM expansion, resulting in a relative reduction in SMC content per MP area. An additional possibility, particularly in the light of negative enrichment of cellular respiration and mitochondrial pathways in STR MP, is an altered functionality of MP SMCs. Indeed, top decreased proteins, including OPA1, DMPK, and ACTN2, have important functions in energy efficiency, contractility, and muscle function; functional loss of these proteins is associated with heart failure, myopathies, and hypomobility of gastrointestinal smooth muscle (56–59). Other top decreased proteins in STR MP are involved in maintaining redox balance that, if disrupted, leads to oxidative stress and mitochondrial dysfunction. Indeed, mitochondrial dysfunction is implicated in IBD inflammation (60), fibrosis (61–63), and cardiomyocyte hypertrophy (62), and mitochondria-targeted therapies are currently under exploration for multiple disorders (64, 65). Overall, the observed reduction in these key SMC proteins in STR MP could reflect impaired muscular function and/or disrupted energy provision in SMCs. A cause-effect relationship of possible SMC dysfunction and hypertrophy in CD-associated strictures, and its potential reversibility, is not known and highlights an area warranting further investigation.

Beyond layer-predominant features, there were also changes shared across the layers, with the largest intersection comprising concordantly increased proteins. Increased immune-related and ER proteins were prominently represented in both layers and were strongly correlated within each layer, suggestive of shared biological process such as immune-stromal infiltrates across layers. While increases in ER proteins may be expected in a highly anabolic and secretory environment, these are proteins with diverse roles in protein folding, processing, and trafficking. Several concordantly increased proteins participate in ER stress/unfolded protein response (UPR) pathways, processes implicated in fibrotic conditions (66–68). These proteins displayed higher fold changes and top ranking in the SM layer, raising the possibility of more pronounced ER stress/UPR signaling in SM of fibrotic strictures. Indeed, proteins in UPR pathways and an ER stress–inducible protein were among the most highly DE proteins in this layer.

Moreover, the concordantly increased ER proteins in STR SM included chaperones and folding enzymes, several of which also exhibit substrate specificity such as that toward integrins, cytokines, and ECM proteins. This includes the B cell–associated chaperone MZB1 (69, 70), which was markedly increased in both layers and was the highest concordantly increased DE protein. Fibroblast-associated TXNDC5, which in addition to its ER chaperone function has antifibrotic potential due to involvement of TGF-β responses and folding of fibrogenic proteins (40), was also highly concordantly increased in STR tissue. Whether the marked increase in these proteins is a cause or effect of the profibrotic environment in STR SM is currently unknown. However, that specific ER chaperones, such as collagen chaperones (HSP47) and UPR inhibition (IRE1 pathway), are currently in clinical trials for fibrotic disease highlights the potential clinical relevance of understanding their role in strictures (71).

Another protein category shared across layers in strictures was decreased lipid transporters. It remains unclear whether this reflects altered metabolic programming or is secondary to changes in adipose tissue associated with strictures. For example, STR SM is associated with reduced adipocytes (72), while creeping fat is commonly observed in CD and in strictures (73). Free fatty acids released from creeping fat have been reported to stimulate MP SMC hyperplasia (18). This raises the possibility of a compensatory downregulation of lipid transporters in the MP due to its proximity to creeping fat. Notably, the lipid transporters FABP1 and — in line with reports of CD strictures (74) — FABP6 were markedly decreased across both layers. These belong to the fatty acid–binding protein family linked to PPAR signaling (75) that promotes antifibrotic programs. Indeed, PPAR agonists are currently being evaluated in clinical trials for liver fibrosis (71), highlighting the potential relevance of this pathway for CD stricture therapies.

Our use of independent control tissue, as well as paired NSTR tissue located adjacent or distal to the patient’s stricture, uncovered a progressive pattern of differential protein expression relative to stricture proximity. The proteins exhibiting progressively increased expression closer to the stricture included a subset of top increased ER-, ECM-, and muscle-associated proteins. This was mirrored by progressively decreased expression of top decreased DE proteins linked to functional and structural homeostasis. These observations may indicate progressive profibrotic remodeling in NSTR tissue as proximity to STR tissue increases. However, future studies incorporating longitudinal biopsy sampling will be required to establish any temporal relationship between fibrosis initiation and evolution. Interestingly, gradual changes in immune-related proteins were less prominent than, for example, ECM and ER/chaperone proteins, and many top increased immune proteins such as IL-16 did not pass our refinement process. Furthermore, patterns suggestive of relative enrichment of SMCs and other contractile cells, such as myofibroblasts, in tissue adjacent to the stricture were also apparent. This aligns with reports of hyperplasia in the muscularis mucosae of regions adjacent to strictures (22). Furthermore, several proteins with progressively increased expression in STR SM, including ITGA8, THBS2, and HOPX, have reported links to muscle cells (42, 76, 77). These may offer additional clues to potential factors involved in SMC hyperplasia in CD-associated strictures and are potential candidates for functional studies.

Our findings are intriguing in light of the immense knowledge gap regarding stricture initiation and progression and may offer some tissue-layer-wise insight into events in the pathological process and provide a framework for future longitudinal and functional analyses. It currently remains unknown whether the progressive patterns in NSTR tissue observed here reflect changes that will culminate in a stricture or are spill-over effects from the stricture’s environment to adjacent tissue in a gradient fashion. Some patterns may also represent compensatory mechanisms that ultimately fail to repress stricture development.

This study was exploratory, coupling LMD with 2 MS methods on formalin-fixed, paraffin-embedded (FFPE) tissue to provide unbiased proteomic data from a limited cohort, and as such has limitations. While the observed layer-specific proteomic trends are intriguing, they are correlative and descriptive in nature. The study did not include longitudinal sampling, and therefore the temporal sequence of molecular events cannot be inferred. In addition, protein abundances are relative rather than absolute measures. Therefore, a high degree of DE does not necessarily correspond to high absolute protein levels in the tissue. Whether the observed DE reflects changes on a per-cell level, in tissue composition, or both cannot be concluded from this study. Patient heterogeneity with respect to disease duration and therapeutic exposure may contribute to variability in observed proteomic signatures and could not be systematically evaluated given the cohort size. However, a strength of the experimental design in our DE analysis was use of paired control tissue from the same individual, thereby controlling for some factors that may influence DE (e.g., sex, age, treatment, genetic background). We also assessed DE using independent non-IBD control ileal tissue. While this provides experimental value as control tissue, it includes the caveat that it is from colorectal cancer resections and may differ from truly healthy ileum or “baseline.” Notably, the majority of the top DE proteins discussed in the text showed consistent directionality when compared with both paired and independent control samples, supporting the robustness of the findings.

Overall, our unbiased proteomic analysis on LMD STR and NSTR tissue layers reveals that proteome changes in CD-associated strictures occur in a tissue-layer-specific manner and differ with the proximity to a developed stricture. Our microdissection approach has, for the first time to our knowledge, allowed characterization of proteome changes in SMC-related proteins in the MP layer of STR tissue, providing insight previously hindered by the inevitable loss of these cells during tissue dissociation (55). We reveal stricture-associated DE proteins and their related biological processes that can be investigated as stricture-targeted therapies, particularly those addressing ECM-cell interactions and muscle changes. Further studies employing targeted approaches or integrating spatial or compartmentalized information with single-cell data may further complement our findings. While FFPE tissue may now be amenable to single-nucleus RNA-seq, LMD of viable tissue prepared as precision-cut slices could, in principle, enable compartment-resolved single-nucleus/single-cell RNA-seq or even single-cell proteomics of microscopically dissected layers. In parallel, spatial omic technologies have emerged as powerful tools that allow cross-referencing with LMD-derived unbiased data or single-cell datasets. Together, such multimodal approaches could provide complementary cell-specific and layer-specific insights into fibrotic remodeling and stricture pathogenesis, building on the layer-specific proteomic framework established here. Indeed, further analysis of stricture-associated DE proteins such as those identified here may open avenues to delay, prevent, or treat CD-associated strictures.

## Methods

### Sex as a biological variable.

Our study included both male and female participants; however, sex was not considered as a biological variable.

### Study population and tissue blocks.

Resected ileal tissue from 12 patients with CD undergoing stricture-related surgery was the source of STR and paired NSTR tissue (Supplemental Figure 1 and Supplemental Table 1). Surgically resected ileal tissue from 8 patients with colorectal cancer was used as independent control (CTRL) tissue (Supplemental Figure 1 and Supplemental Table 1). Surgery was performed at the Department of Surgery, Sahlgrenska Östra Hospital and FFPE surgical tissue was deposited in the Sahlgrenska University Hospital biobank until use. All included tissue samples consisted of resected full-thickness ileal tissue (Supplemental Figure 1). Detailed information on tissue blocks, histology, sectioning, staining, imaging, and layer definitions is in the Supplemental Methods.

### LMD of tissue layers.

Pilot experiments showed that the small amount of protein in the microdissected samples, as well as protein assay interference from the H&E stain, necessitated standardizing samples with methodology other than traditional protein determination (Supplemental Methods). Alternate methodology also had to account for differences in tissue density, as STR tissue was denser than NSTR and CTRL (Figure 1A). We thus developed an imaging-based standardization method in which serial sections from each FFPE sample (H&E reference slides; Figure 1A) were first imaged and layers were subsequently outlined and analyzed using a custom profile in StrataQuest software (TissueGnostics) (Supplemental Methods). After estimating the net tissue coverage of preliminarily drawn layer regions, each region’s lateral extent (perpendicular to the radial axis) was iteratively adjusted until a standardized net tissue coverage of 44 mm^2^ from one or more serial sections was achieved, and this was repeated across the cohort (Figure 1A, Supplemental Methods, and Supplemental Figure 1, B and C). This approach thus ensured equal tissue coverage across all samples. LMD was conducted with a PALM Microbeam system (Carl Zeiss GmbH) with a pulsed 355 nm laser controlled by PALM RoboSoftware (Carl Zeiss MicroImaging GmbH). At the LMD workstation and using the tissue mounted on the membrane slide, the outlines of the final standardized SM and MP regions (44 mm^2^ net tissue coverage each) were manually retraced as cutting line elements in the LMD software (RoboSoftware), and the corresponding layers were excised (Figure 1B). Tissue was immediately transferred to tubes containing 100 μL sodium deoxycholate with 50 mM trietylammoniumbicarbonate and stored at –80°C until proteomic sample preparation. From STR samples, additional tissue was dissected and collected separately (dry) for the TMT boost (Supplemental Methods).

### Proteomic samples, data processing, and analysis.

Proteomic analysis was performed using 2 methods, TMT-MS and TIMS-TOF-MS (Supplemental Methods). The label-free TIMS-TOF-MS method was performed in parallel to TMT-labeled samples using one-tenth of the volume of exactly the same samples (Figure 1C) as an internal validation and to increase data analysis robustness. Briefly, dissected tissue samples were prepared and digested with trypsin, where one aliquot was set aside for TIMS-TOF-MS while the remaining volumes continued following the TMT sample preparation protocol (Figure 1C). Representative reference samples (Figure 1C, “R”) were created for each layer by pooling equal aliquots from all individual samples. The reference samples, along with the individual samples and booster samples (Figure 1C, “B”; Supplemental Methods), were labeled using TMTpro 18-plex isobaric mass tagging reagents (Thermo Fisher Scientific), combined into 4 sets (Figure 1C) and concentrated using vacuum centrifugation. The combined sample sets were fractionated and concatenated into 20 fractions using basic reversed-phase chromatography (bRP-LC). The TMT sets were dried and reconstituted in 3% acetonitrile/0.2% formic acid for nLC-MS3 analysis. Subsequent TMT-LC-MS3 and data-independent acquisition (DIA) TIMS-TOF-MS and data analysis is described in Supplemental Methods. Protein-level data tables from the 2 MS methods were further preprocessed and normalized at the protein/protein group level to generate the final datasets used for analysis (Supplemental Methods).

### Statistics.

To identify DE proteins in each layer, we fitted a linear mixed-effects model (*lmer* function; lme4 R package) with Group (STR, NSTR, CTRL) and TMT set as fixed effects and Individual as a random effect [Abundance ~ Group + TMT_set + (1 | id)]. For proteins quantified exclusively in one plex set, the TMT set term was omitted. The fitted model was then used as input to the *emmeans* R package to estimate marginal means [*emmeans()*] and compute pairwise contrasts [*contrast()*] for STRvNSTR and STRvCTRL comparisons (ΔEMM) and statistics. Default settings were used, and degrees of freedom were estimated using the Kenward-Roger method. Finally, obtained *P* values for each contrast were FDR adjusted using the Benjamini-Hochberg method. Proteins with FDR of less than 0.05 and |ΔEMM| of greater than 0.3 were considered DE. Subsequent complementary analysis of stricture versus distal NSTR (STRvDist) and stricture versus adjacent NSTR (STRvAdj) comparison were conducted as above but with NSTR replaced by Dist and Adj. The identical procedures were used for DE analysis of the TIMS-TOF-MS data. The integration of DE results from the 2 MS methodologies (TMT-MS and TIMS-TOF-MS), aimed at identifying proteins that were DE at the combined level in each layer, is detailed in the Supplemental Methods. Briefly, the layer-wise DE results tables were joined by Accession, followed by *P*-value merging using the DPM method (78) and calculation of the WE (i.e., weighted average of ΔEMM; ≈ weighted average of log_2_FC). This procedure was performed separately for each comparison. Proteins with an FDR-adjusted merged *P* value of less than 0.05 and |WE| of 0.3 or greater were considered significant at the combined level. The numbers of DE proteins before and after applying these combined-level thresholds are shown in Supplemental Figures 6 and 7. The resulting combined-level DE proteins were scored and ranked as described in the Supplemental Methods. Downstream bioinformatic analysis and visualization were performed in the R environment. Information about specific analyses, R packages used and database access and extraction, are detailed in the Supplemental Methods. Statistical analysis details can be found in the relevant panels within the Supporting Data Values file. All box-and-whisker plots indicate the median (line) and IQR (box bounds), with whiskers extending to the most extreme values within 1.5 times the IQR.

### Study approval.

The study was conducted according to the Declaration of Helsinki. Patients were included under permit 085–11 approved by the Ethical Review Board in Gothenburg. All patients gave informed written consent to participate prior to inclusion.

### Data availability.

The MS proteomics data have been deposited to the MassIVE repository. The TIMS-TOF-MS datasets can be accessed with the dataset identifier MSV000100450 and the TMT-MS datasets with MSV000100468. Values for all data points in graphs are reported in the Supporting Data Values file.

## Author contributions

MJW conceptualized and supervised the project and secured funding. JA and MJW designed experiments, interpreted data, and wrote and edited the original manuscript draft. JA performed experiments, analyzed data, and drafted figures. CSW gave conceptual and technical advice. MÖ, HDLC, and EBL evaluated tissue samples and managed clinical databases. All authors critically reviewed manuscript drafts and approved the final manuscript.

## Funding support

The following entities provided funding support.

- Swedish Cancer Society (Cancerfonden) grants CAN 2015/463 and CAN 2018/372 (to MJW).
- National Microscopy Infrastructure (NMI) grant VR-RFI 2019-00022 (to the Centre for Cellular Imaging at the University of Gothenburg).
- SciLifeLab, Project 7809 (to the Proteomics Core Facility, Sahlgrenska Academy, Gothenburg University).
- SciLifeLab and BioMS grant (to the Proteomics Core Facility, Sahlgrenska Academy, Gothenburg University).

## Supplementary Material

Supplemental data

Supporting data values

## Version 1 02/10/2026

In-Press Preview

## Version 2 03/23/2026

Electronic publication
