# Supplementary material for: Tissue-layer-resolved proteome landscape of Crohn’s disease strictures highlights potential drivers of fibrosis progression
Source: JCI Insight. 2026 Feb 10;11(6):e202461. doi: 10.1172/jci.insight.202461 (PMC13043094; doi:10.1172/jci.insight.202461)
Supplement: Supplemental data [file jciinsight-11-202461-s361.pdf]

Supplemental Information

Supplementary Materials and Methods 3
1. FFPE tissue blocks 3
2. Histology, Sectioning, staining and scanning 3
3. Tissue layer definition protocol 4
Muscularis Propria (MP) 4
Submucosa (SM) 4
Definition of regions 5
4. Initial image analysis and Standardization 5
5. Laser microdissection (LMD) 6
6. Creation of pooled reference, TMT boost and final TMT plex layout for proteome analysis....7
7. Proteomic sample preparation and mass spectrometry 7
LC-MS3 analysis QMS 8
LC-MS3 data analysis..9
DIA MS analysis 9
DIA data analysis 9
8. Further processing and normalization of protein-level data 10
TMT-MS data..10
TIMS-TOF-MS data 10
9. Integration TMT-MS and TIMS-TOF-MS results 11
Matching Accession between TMT-MS and TIMS-TOF-MS data 11
DE results integration..11
Scoring and ranking 12
10. Bioinformatic analyses 12
Accounting for TMT set..12
PCA Analysis and Correlation Circle Plot..12
Gene/Protein Set Enrichment Analysis (GSEA) and pathway networks 13
Gene Set Variation Analysis (GSVA)..14
Combined level analyses..14
STRING Protein-Protein Interaction (PPI) networks 14
Correlation network analysis..14
Trend analysis in the SM layer..15
11. Additional information..16
Figures 16

R packages for analysis and visualization: 17
Databases 17
HPA Cell markers 17
Matrisome 17
Identification of secreted and adhesion-related proteins 18
Supplementary Results 19
1. Parallel TIMS-TOF-MS corroborates differential protein expression trends obtained using TMT-MS analysis 19
2. Pathway enrichment supports SM stricture shifts from homeostasis toward anabolism with enhanced secretory and immune activities 19
3. Mitochondrial metabolism pathways are reduced while immune-related processes are increased in the MP of strictured tissue 20
4. Integration of TMT-MS and TIMS-TOF-MS datasets 20
5. Intercorrelated, highly DE secreted and adhesion proteins reflect parallel processes occurring in STR SM 21
6. Network clustering reveals interrelated DE proteins potentially reflecting distinct yet parallel processes occurring in STR MP 22
7. Interlayer analysis highlights protein categories with shared expression in the SM and MP layers 22
References for Supplemental information 24
Supplemental Figures 1–11 25
Supplemental Tables 1–13 47
References for Supplemental Tables 61

Supplementary Materials and Methods

1. FFPE tissue blocks

FFPE tissue blocks from the Sahlgrenska University Hospital Biobank, including the 12 CD patients and 8 controls included in the study (Supplemental Table 1; Supplemental Figure 1), were reviewed by a pathologist and chosen if they contain full-depth (transmural) ileum with all histological layers (Supplemental Figure 1, A–C) and, for CD patients, a block from the stricture site (STR) and NSTR tissue from the same surgery was required (Supplemental Figure 1B). Primary interest for NSTR tissue was a separate block taken near the surgical resection margin (Distal NSTR, "Dist"; Supplemental Figure 1D); if unavailable, visually non-strictured tissue within the same block as the STR tissue was used (Adjacent NSTR, "Adj"; Supplemental Figure 1D). Eight of the CD patients had NSTR tissue available as a separate block and the remaining four had NSTR tissue present in the same block as the STR tissue (Supplemental Figure 1D). Reference slides stored in the biobank associated with each block the pathologist suggested were assessed to confirm the inclusion criteria and tissue quality for the final cohort (Supplemental Figure 1; Supplemental Table 1).

2. Histology, Sectioning, staining and scanning

Tissue blocks were sectioned, mounted, and stained at HistoCenter (Mölndal, Sweden). Two reference slides (4 μm sections) were prepared per block (Figure 1A). One of the two reference slides from each FFPE block was stained with Masson's Trichrome (MT) and the other with Hematoxylin & Eosin (H&E) following the standard protocol of HistoCenter (Mölndal, Sweden) (Figure 1A). Following initial imaging analysis (see below), additional 10 μm serial sections (2-5 slides) were cut from each block and mounted onto membrane slides (MembraneSlide NF 1.0 PEN; Carl Zeiss MicroImaging GmbH, Germany) (Figure 1B). The PEN membrane slides were stained with H&E, immediately scanned at 5X (TissueFAXS) and stored in air-tight tubes at -80°C until laser microdissection microscopy (LMD).

Where FFPE blocks contained large pieces of tissue, sections were split and distributed across multiple standard-size slides to cover the entire tissue. The reference slides were scanned at 20X magnification (Figure 1A) using a TissueFAXS system (TissueGnostics; Vienna, Austria) consisting of an Axio Imager Z2 microscope (Zeiss; Jena, Germany) equipped with a PixeLink PL-D674CU-

CYL-07451 color camera (PixeLINK; Ottawa, Canada). The whole-slide images were analyzed using StrataQuest software (7.1.1.129; TissueGnostics GmbH, Vienna, Austria) as described below. The 1-5 additional serial sections containing 10 µm sections mounted onto PEN membrane slides for LCM (Figure 1B) were UV-treated (254 nm for 30 min) prior to mounting on PEN according to Zeiss's recommendations. The mounted slides were dried in a vertical position overnight at 56°C before staining and were stained with H&E using the HistoCenter standard routine except that the final xylene step was omitted during dehydration. The slides were scanned at 5X and stored until LMD.

3. Tissue layer definition protocol

Given the altered layer architecture in strictures, some rules and definitions were applied based on our aim to be consistent when drawing regions as described below. See Supplemental Figure 1A for layer schematic in control tissue.

Muscularis Propria (MP)

Tissue was considered MP if coherent and organized muscle bundles arranged together were discernable. Anything lying in between the limits (upper luminal side and lower serosal side) of this coherent area (i.e. nerve and connective tissue) were regarded as MP. When drawing the line between MP and SM, we limited ourselves to follow the top curvature and go as deep as was technically feasible.

Submucosa (SM)

Hypertrophied, distorted and fibrotic muscularis mucosae (MM) in stricture samples often appeared coherent with fibromuscular changes in the SM, without any distinct boundary between SM and MM. To treat both strictures and controls in the same manner, we thus included the MM in the SM tissue type (Figure 1B) and use this definition of SM throughout.

In practice, the separation of the SM tissue, as defined here, from the overlying lamina propria and epithelium meant drawing a line along the luminal side of the MM for control samples and along the luminal edges of the confluent MM-like fibromuscular tissue in stricture samples. In ulcerated tissue, inflammatory infiltrates and granulation tissue above or penetrating through the MM-like tissue were avoided. Any confluent lymphoid tissue in contact with/directly beneath the lumen as well as blood within tissue (outside blood vessels) were cut around to avoid. The lower limitation of the SM was defined by the luminal contour of the MP. Fibromuscular tissue coherent with SM but situated outside of the defined MP (“penetrating fibrosis” (Figure 1B, right)

were defined as SM but limited to an extrapolated line between the outer two edges of external MP. The assessments of any layer boundaries were aided by cross-referencing the staining pattern on the scanned MT reference slides (Figure 1A).

Definition of regions

In general, each region for the main analysis was generally drawn as single, uninterrupted areas per slide, with SM and MP sharing the same border, as defined above (Figure 1A). However, due to variability in SM and MP thickness across samples, the regions differed in length, typically with the shorter region (often MP) covering only part of the SM–MP border of the longer region (SM). Whenever smaller regions were pooled to reach 44 mm^2, identical regions from serial sections were used. Several stricture samples had SM or MP that was far beyond 44 mm^2. In such a case, another region (R2) covering the excess area (10-50 mm^2 of effective tissue area) was drawn to be included in the TMT-boost (only applicable to STR samples; see separate section). For drawing the regions for STR samples containing both STR and Adj NSTR (as in Figure 1, A and B; Supplemental Figure 1D), a preliminary line (perpendicular to the intestinal wall) was drawn at the transition zone. This line served as the starting point for drawing the STR and NSTR regions (which then extended in either direction).

4. Initial image analysis and Standardization

Initial imaging analysis of the H&E stained whole-slide reference images (Figure 1A) was performed using StrataQuest software (7.1.1.129; TissueGnostics, Vienna). Two standardized tissue regions on each sample's image, one submucosal region (the SM layer) including muscularis mucosae and submucosa, and one muscularis propria region (the MP layer) (Figure 1B, right) were delineated to standardize for protein content across samples. The regions were manually drawn in the software according to a predefined protocol. To minimize selection bias, regions were drawn by following the natural boundaries of each layer and expanded laterally to ensure full-depth coverage and continuity, respectively.

Standardizing protein content across samples necessitated accounting for differences in tissue density (foreground-to-background area ratio; Figure 1A, right). We thus set out to quantitate protein in the microdissected tissue samples using methods common to proteomics such as the BCA assay. However, our attempts to quantify the small amounts of protein in the LMD samples proved unreliable based on both low amount of protein and apparent interference of

H&E stain with the protein assay. We thus developed an imaging-based method to standardize across samples as follows. Preliminary regions were first drawn, and tissue density and net foreground tissue coverage was estimated using a custom project profile in StrataQuest software. The original color image was transformed into a median-filtered (Kernell radius = 2 pixels) grey-scale image, and the background threshold was automatically detected using the Otsu algorithm. Tissue was then defined as pixels with a grey-scale intensity above this threshold. Regions were iteratively (manually) adjusted until re-analysis confirmed that each contained a total of 44 mm^2 of net foreground tissue coverage (Figure 1A). These final regions were defined as R1 (one R1 per layer for each sample). An example showing the outline of the defined regions from stricture tissue (STR MP and STR SM) and from non-stricture tissue (NSTR MP and NSTR SM) using a stricture slide containing both stricture tissue and adjacent non-stricture tissue from the same FFPE block is shown in (Figure 1, A and B). In cases where 44 mm^2 of foreground area could not be achieved from a single slide, identical subregions from serial sections were pooled to match the tissue content of STR samples, which generally provided more tissue. In these cases, the region was reduced until the tissue content was evenly divisible with 44 such that pooled regions from multiple slides collectively achieved 44 mm^2 (22 mm^2 from two slides, 14.67 mm^2 from three slides and 11 mm^2 from four slides). This approach ensured maximum tissue use and sufficient tissue collection from each sample. For STR images, any excess SM and MP tissue left on the image after R1 was defined and annotated as R2.

5. Laser microdissection (LMD)

As a final imaging step before LMD, color-coded visual guides were created by manually redrawing the outlines of the final regions on top of the pre-scanned membrane slide images in StrataQuest (Figure 1B). These scans better reflect the visual appearance at the LMD workstation, where the thicker 10 µm sections (Figure 1B) (compared to the 4 µm reference slides; Figure 1A) appeared darker with less contrast. The guides facilitated work at the LMD workstation, improving accuracy in replicating region boundaries for complex stricture samples and enhancing the overall reliability of the LMD process.

For LMD, the frozen membrane slides (stored in sealed tubes) were acclimated to RT for 30 min. With the visual LMD guides at hand (Figure 1B), the standardized regions containing 44 mm^2 of effective tissue (distributed on 1-5 serial sections depending on the tissue content of the slide as described above) of the SM and MP layers were manually redrawn as line elements within the

RoboSoftware and dissected from the tissue (Figure 1B). Dissected tissue pieces were gently picked up with tweezers under a stereomicroscope and immediately transferred to tubes containing 100 ul SDOC and stored at -80°C until proteomic sample preparation. From STR samples, additional tissue was dissected and transferred to separate (dry) tubes to be used for the TMT-boost samples (see below). This included an additional 44 mm^2 (R1 region, identical to the region cut for the main analysis) per tissue type (SM and MP) dissected from a serial section. In addition, when effective tissue regions on a given slide exceeded 44 mm^2, these "excess" tissue regions (R2) were collected in separate tubes to use in creating the TMT-boost samples described below. These dry pieces of additional tissue in separate tubes were saved at -80°C until use in the TMT-boost samples.

6. Creation of pooled reference, TMT boost and final TMT plex layout for proteome analysis

To increase the sensitivity for quantifying STR-specific proteins in the analysis, we used one TMT-label per set of 18-plex for a TMT boost sample (1, 2) (Figure 1C, "B"). Each TMT boost sample contained roughly 9.5 times the amount of tissue compared to the main samples, thus increasing the peptide material available for sequence analysis. Since all TMT labeled samples within a plex set are mixed and analyzed in parallel in the mass spectrometer, the effect of introducing more material of a given peptide to any of the samples will increase the summed intensity for a given peptide at the MS1 level. As the acquisition is data-dependent, the increased MS1 intensity of a particular peptide increases the probability of it being above the detection level and selected for fragmentation and identification; the contribution of each sample is determined based on the TMT label.

The boost samples were made using the additional STR tissue that was laser-microdissected in parallel with the main samples. Boost samples consisted primarily of the main region (R1) from an additional serial section slide and pieces representing excess tissue in stricture slides that exceeded the 44 mm^2 needed for the main sample (R2 as described above). The TMT boost samples were created separately for SM and MP.

7. Proteomic sample preparation and mass spectrometry

Samples were treated with 5 mM dithiothreitol (DTT) in 0.5 % sodium deoxycholate (SDOC) and 100 mM triethylammonium bicarbonate (TEAB) at 56°C for 30 min to reduce disulfide bindings

and free cysteine residues were alkylated with 20 mM iodoacetamide (IAA) at room temperature for 30 min in the dark. Remaining IAA was quenched with 5 mM DTT. Proteins were digested with trypsin (1:100 enzyme:protein ratio, MS-grade, Promega) overnight and the next day with another portion of trypsin for four hours. 8% of each sample was used for data independent acquisition (DIA) analysis.

Representative reference samples (Figure 1C, “R”) were prepared per layer containing equal amounts from all individual samples. Peptides were labelled using TMTpro 18-plex isobaric mass tagging reagents (Thermo Scientific) according to the manufacturer’s instructions. Labelled samples were combined into four sets (Figure 1C: SM1 and SM2; MP1 and MP2) including the respective reference sample and booster sample, concentrated using vacuum centrifugation, and SDOC was removed by acidification with 10% trifluoroacetic acid (TFA) and subsequent centrifugation. The labelled pooled samples were further purified using HiPPR Detergent Removal Resin and Pierce peptide desalting spin columns (both Thermo Scientific) according to the manufacturer’s instructions. The combined TMT-labelled samples were fractionated into 70 primary fractions by basic reversed-phase chromatography (bRP-LC) using a Dionex Ultimate 3000 UPLC system (Thermo Fischer Scientific). Peptide separations were performed using a reversed-phase XBridge BEH C18 column (3.5 µm, 3.0x150 mm, Waters Corporation) and a linear gradient from 3% to 40% solvent B over 18 min followed by an increase to 100% B over 5 min and 100% B for 5 min at a flow of 400 µL/min. Solvent A was 10 mM ammonium formate buffer at pH 10.00 and solvent B was 90% acetonitrile, 10% 10 mM ammonium formate at pH 10.00. The 70 primary fractions were concatenated into 20 fractions. All were evaporated and reconstituted in 3% acetonitrile, 0.2% formic acid for nLC-MS3 analysis.

LC-MS3 analysis QMS

Fractions were analysed on an Orbitrap Eclipse™ Tribrid™ mass spectrometer equipped with a FAIMS Pro ion mobility system and interfaced with an Easy-nLC1200 liquid chromatography system (all Thermo Fisher Scientific). Peptides were trapped on an Acclaim Pepmap 100 C18 trap column (100 µm x 2 cm, particle size 5 µm, Thermo Fisher Scientific) and separated on an in-house packed analytical column (50 cm x 75 µm, particle size 3 µm, Reprosil-Pur C18, Dr. Maisch, Ammerbuch-Entringen, Germany) using a stepped gradient from 4% to 28% acetonitrile in 0.2% formic acid over 77 min at a flow of 300 nL/min, followed by increase to 80% acetonitrile for 13 min. FAIMS Pro was alternating between the compensation voltages (CV) of -50 and -70, and the same data-dependent settings were used at both CVs. The precursor ion mass spectra were acquired at a resolution of 120 000 and an m/z range of 375-1375. Using a cycle time of 1.5

seconds, the most abundant precursors with charges 2–7 were isolated with an m/z window of 0.7 and fragmented by collision induced dissociation (CID) set to 35%. Fragment spectra were recorded in the ion trap at Rapid scan rate. Dynamic exclusion was set to 60 sec. The ten most abundant MS2 fragment ions were isolated using multi-notch isolation for further MS3 fragmentation. MS3 fragmentation was performed using higher-energy collision dissociation (HCD) at 55% and the MS3 spectra were recorded in the Orbitrap at 50 000 resolution and an m/z range of 100–500.

LC-MS3 data analysis

Data analysis was performed using Proteome Discoverer (v2.4, Thermo Fisher Scientific). The data were matched against Human SwissProt database (20361 entries, May 2022) using Sequest as a search engine with a precursor tolerance of 5 ppm and a fragment ion tolerance of 0.6 Da. Tryptic peptides were accepted with 1 missed cleavage. Methionine oxidation was set as a variable modification and cysteine carbamidomethylation, TMTpro on lysine and peptide N-termini were set as fixed modifications. Percolator was used for PSM validation with a strict FDR threshold of 1%. For quantification, TMT reporter ions were identified in the MS3 HCD spectra with 3 mmu mass tolerance, the TMT reporter intensity values for each sample were normalized on the total peptide amount and the SPS threshold was set to 65%. Only unique peptides were used for relative quantification and proteins were required to pass a protein FDR of 1%.

DIA MS analysis

DIA analysis was performed on a TIMS-TOF HT mass spectrometer (Bruker) coupled to an Evosep One (Evosep) liquid chromatography (LC) system. 800 ng of each sample were loaded onto Evotips Pure (Evosep) according to the manufacturer’s instructions. The LC system was operated using the 30 samples per day method (30SPD) on a Pepsep C18 column (15cm x 150 µm ID, 1.5 µm, Bruker). The TIMS-TOF HT was run in dia-PASEF mode using variable isolation windows. The isolation windows were created with py_diAID (0.018) (3), with the recommended default settings, built on a spectral library generated using fresh frozen tissue samples. The collision energy was set from 20 to 59 eV along an ion-mobility range of 0.6 to 1.6 Vs/cm2.

DIA data analysis

The data was matched against the reviewed human database (20426 entries, November 2023) using directDIA analysis in Spectronaut (18.4). SM and MP samples were searched separately. Default settings were used for the data analysis, and maximum missed cleavages allowed was

set to 1. The data were matched against the reviewed human database downloaded from uniport.org (20426 entries, November 2023). Carbamidomethyl on cysteine was set as fixed modification, oxidation of methionine and acetylation of protein N-termini was set as variable modifications. The proteotypicity filter was set to only protein group specific and cross run normalization was performed.

8. Further processing and normalization of protein-level data

TMT-MS data

Processing of TMT-MS data for the SM and MP datasets was performed using identical procedures, but each dataset was normalized separately. First, the data pre-processing involved the removal of common contaminants and identified but not quantified proteins prior to normalization. Next, abundances were normalized to achieve equal total abundance across all samples. Then internal reference scaling (4), using the geometric mean of the reference samples ("R" in Figure 1C), was applied per protein to bring the abundances across the two TMT plex sets to a common scale. As a final normalization step, a trimmed mean of M-values (TMM) normalization was then applied (edgeR package) with missing values replaced by zeros to compute the normalization factors. Finally, the normalized abundances were log_2-transformed, and the reference samples were removed from the data. The effectiveness of the normalization procedures was examined using sample box plots, relative log expression (RLE) plots, and MA plots. Proteins without a gene symbol were omitted.

TIMS-TOF-MS data

Before further processing, common contaminant proteins and proteins with excessive missing values were removed from the TIMS-TOF-MS data to ensure consistency with the treatment of the TMT-MS data, where proteins quantified in only one set were accepted. Specifically, we retained only proteins with ≥ 50 % non-missing values in STR and CTRL groups for STRvCTRL analysis, or ≥ 50 % STR-NSTR pairs with complete observations for the STRvNSTR analysis. The TIMS-TOF-MS data were subsequently log_2-transformed and normalized using cyclic LOESS (limma R package). As with the TMT-MS data, the effectiveness of these normalization procedures was confirmed by assessing aligned sample box plots, relative log expression (RLE) plots, and MA plots.

9. Integration TMT-MS and TIMS-TOF-MS results

Matching Accession between TMT-MS and TIMS-TOF-MS data

In the TMT-MS dataset, each protein/protein group was associated with a single accession, whereas some proteins/protein groups in the TIMS-TOF-MS dataset had multiple accessions. To integrate TMT-MS and TIMS-TOF-MS results, proteins were first matched by single accession. For TIMS-TOF-MS entries with multiple accessions, a match was accepted if at least one accession corresponded to a single TMT-MS accession. Proteins quantified exclusively with TIMS-TOF-MS with multiple accession were excluded from the merged table as was any protein with missing gene symbol. In the final curated and merged SM table, 2,384 proteins were matched as "quantified in both" datasets (Supplemental Figure 2G), of which 2,339 were direct matches between single accessions. An additional 818 proteins were exclusive to the TIMS-TOF-MS dataset, all of which had single accessions. Among the 1,045 DE proteins at the combined level in the SM layer (see below), all but eight had single accessions. All the Top 30 proteins in both directions had single accessions. In the curated and merged MP table, 1,874 proteins were matched as "quantified in both" (Supplemental Figure 2H) with 1,837 being direct single accession matches. A further 847 proteins were exclusive to the TIMS-TOF-MS dataset, all with single accessions. Of the 534 DE proteins at the combined level in the MP layer, all but five had single accessions. Additionally, all Top 30 DE proteins in the negative direction and all but three in the positive direction had single accessions.

DE results integration

Combined-level statistics of TMT-MS and TIMS-TOF-MS data was then calculated separately for each comparison (STRvCTRL, STRvNSTR, STRvDist, STRvAdj) and layer (SM, MP) as follows. The unadjusted p-values obtained from the DE analyses of the TMT-MS and TIMS-TOF-MS datasets were merged using the merge_p_values function from the ActivePathways R package, utilizing the DPM method (5) with a c(1,1) constraint vector. In the input matrices to merge_p_values, missing p-values were replaced with 1 and missing sign of the estimate with 0. The merged p-values were then adjusted for FDR using the Benjamini-Hochberg method. To obtain a combined metric of the magnitude of change for each protein, the average of the estimates (ΔEMM) obtained from DE analysis of each MS dataset, weighted by the number of valid observations per method, was used. This combined estimate is henceforth referred to as Weighted Estimate (WE) and corresponds to a weighted Log_2FC. For proteins with only one available estimate (i.e., a protein quantified with one method only), the available estimate was used. FDR adjusted merged p value < 0.05 and abs(WE) ≥ 0.3 were considered significant at the

combined level. The results of the combined level threshold are displayed in Supplemental Figures 6 and 7.

Scoring and ranking

To rank and prioritize proteins, scores were calculated for combined-level DE proteins in each comparison, direction and layer separately by z-transforming (center and scale) WE and a significance metric and calculating their average. The significance metric constituted of the –$\log_{10}$ of the FDR-adjusted merged p-value multiplied by the sign of the WE. Lastly, since scores were centered around zero a constant, [3*sign(WE)] was added for easier interpretation of the directionality of the score (i.e. high positive score for top increased DE proteins and high negative score for top decreased DE proteins).

10. Bioinformatic analyses

Accounting for TMT set

Initial Principal Component Analysis (PCA) of the normalized TMT-MS datasets revealed separation by group but also separation by TMT set. For DE analysis, TMT set was accounted for in statistical modeling. However, whenever needed to analyze or visualize all samples together, batch correction was applied prior to analysis using the combat function from the sva R package (6) and is indicated in the figure legends. The correction effectively removed the TMT set separation in both layers. For paired sample data, used for correlation network analysis, the within matrix of uncorrected data matrix was used. This effectively canceled out any TMT set effects since each pair was in the same TMT set and analyzed together. For visualization of individual proteins, normalized expression values ($\log_2$ scale) were adjusted by adding the offset between the global mean of all set means and the mean of the specific TMT set. This adjustment removes set-specific shifts. For consistent treatment across datasets, the identical procedures were applied to both TMT-MS and TIMS-TOF-MS data.

PCA Analysis and Correlation Circle Plot

PCA was performed on the batch-corrected datasets as described above, with centering and scaling set to TRUE in all cases. For the correlation circle plots (7, 8), Pearson correlations between each protein and the principal components (PCs) were extracted using the plotVar() function from the mixOmics R package (8). The resulting PC-protein correlation matrix was next subset to proteins annotated as HPA cell markers or matrisome components (see Databases

above) and plotted using ggplot2. Given the partially paired design of our study (i.e., STR and NSTR samples from the same individuals), we also applied multilevel PCA (mixOmics) which is based on the within-individual variation. The multilevel PCA did not change the overall conclusion (not shown).

Gene/Protein Set Enrichment Analysis (GSEA) and pathway networks

For GSEA, the sign of the estimates multiplied by $-\log_{10}$ p-values for each layer and comparison was used to create ranked lists from the TMT-MS data results. Hallmark (9) and C2 [including Reactome (10)] gene set collections in GMT format (version 2.2023.2) were downloaded from the Molecular Signatures Database (MSigDB) on 2024-02-02. GSEA was conducted using the GSEA function of clusterProfiler R package (11), with ranked metrics and gene sets as inputs. Default settings were used with exceptions (minGSSize=5, eps=0, pvalueCutoff = 0.10, pAdjustMethod = “fdr”).

Further processing of the Enrichment results

Due to extensive overlap in enriched Reactome pathways (i.e., shared proteins across gene/protein sets), we applied a filtering step to refine the results and ensure broader coverage of the DE proteins. This was done separately for each comparison. First, we retained only pathways with an FDR < 0.01. Next, we separated results into positive and negative enrichment groups, sorting each by absolute NES from highest to lowest.

Two running vectors were then initialized: one for cumulative core enrichment proteins and another for cumulative DE core enrichment proteins. We then applied a row-wise function to the core enrichment column. For each row, we compared the core enriched proteins to the running vectors, recorded the number of unique core enriched proteins not already in the vectors, and updated the vectors accordingly. Finally, we retained only those pathways that contributed with at least one unique DE core enriched protein not already captured by the preceding pathways. For Hallmark pathways, we retained terms where at least one comparison showed an FDR < 0.05. In the final curated and filtered list, each pathway was therefore significant (FDR < 0.05) in at least one comparison. When only one comparison met this threshold, the other was also displayed if its FDR was between 0.05 and 0.10.

Pathway network

For enrichment maps, the four enrichment result tables per layer (Hallmark and Reactome; STRvNSTR and STRvCTRL) were merged using the merge_result function from ClusterProfiler. The similarity matrix was then computed using the pairwise_termsim function (enrichPlot R package; default settings). Finally, we used emapplot (enrichPlot R package) to visualize the similarity matrix, with min_edge set to 0.1 and showCategory set to the filtered pathways.

Gene Set Variation Analysis (GSVA)

For GSVA (using the R package of the same name (12)), we first created a gsvaParam object using the SM batch-corrected TMT-MS dataset (100% complete observations) and the Hallmark and Reactome gene sets (see GSEA), with default settings except for minSize = 5 and maxSize = 500. This object was then passed to the gsva function to generate pathway scores across samples was subsequently used for correlating pathways with SM PC3 scores.

Combined level analyses

STRING Protein-Protein Interaction (PPI) networks

PPI networks and clustering were conducted using the STRING online platform (13). Networks were generated using an interaction score of ≥ 0.4. Clustering was performed using the Markov Cluster Algorithm (MCL) with an inflation parameter of 3. The resulting interaction networks, cluster assignments, and cluster metadata (including auto-annotations) were exported and further processed in Microsoft Excel or R. Network visualizations were carried out using the ggraph package in R. Clusters were annotated based on STRING auto-annotations obtained with the cluster metadata, summarized as general umbrella terms reflecting the canonical functions of the proteins within each cluster. Based on these annotations and the clusters' positions within the network, they were further manually grouped into higher-order clusters or broader functional domains.

Correlation network analysis

The paired samples data (STR and NSTR column) of the TMT-MS and TIMS-TOF-MS datasets, respectively, were used as input to the pca function of the MixOmics R package (8) using Individual ID as the multilevel argument and center and scale set to TRUE, to extract the within-variation matrices (14) [Xw]. Zeros in the extracted Xw matrices (introduced when the value of one of two paired samples from one individual was missing), were then replaced with NA. Two Spearman's rank correlation matrices per layer (one for TMT-MS and one for TIMS-TOF-MS) were then generated using complete pairwise observation.

For each pair of variables, the Spearman rank coefficients from the TMT-MS and TIMS-TOF-MS datasets were averaged, with each coefficient weighted by the number of complete observations from the correlation in each MS dataset. If only one correlation coefficient was available (TMT-MS or TIMS-TOF-MS), it was used as the weighted average but indicated by a thinner edge in Supplemental Figure 9, see below. The weighted correlation coefficients were then used for downstream correlation network analysis. Correlation networks were built using weighted average Spearman rank correlation coefficient > 0.85 as edges. Clustering was performed using the MCL algorithm with an inflation parameter of 3. A meta-cluster network was then generated by first removing all within-cluster edges. Subsequently, edges between any two clusters were replaced by a single edge representing the sum of their correlation coefficients. Visualizations of protein and cluster network were carried out using the ggraph package in R.

Due to mismatches in valid versus missing observations across variable combinations, the number of complete observations for some combination of variables was low. Such information was implemented in the edge meta (line type) data in Supplemental Figure 9 as follows. Thick (whether solid or dashed) line represents a true weighted average (weighted average of each correlation coefficient obtained from TMT-MS-data and TIMS-MS-data). For these correlations, the data was defined as limited if both the TMT-MS-data and TIMS-MS-data correlation coefficients were based on 12 or fewer complete observations. Thin (whether solid or dashed) line means a correlation coefficient was only available from one dataset (i.e. TMT-MS-data or TIMS-MS-data) for that combination of variables. For these, the data was defined as limited if the correlation coefficient was based on fewer than 20 complete observations (out of 24).

Trend analysis in the SM layer

To evaluate progressive trends from CTRL to STR across the four tissue groups (CTRL, DIST, ADJ, and STR), WE comparisons (STRvCTRL, STRvDIST, STRvADJ) were transformed into group-level values. Specifically, we inverted the direction of these comparisons to obtain CTRLvSTR, DISTvSTR, and ADJvSTR, expressing each group's relative expression as a difference from STR (set to 0). We then subtracted the inverted STRvCTRL WE from all four groups, using CTRL as the reference (baseline = 0). This allowed us to focus on relative differences among CTRL, DIST, ADJ and STR rather than specific comparisons.

To enable two-dimensional visualization of the four tissue groups, expression values were scaled relative to the CTRL–STR range. This approach, similar to min-max scaling, used CTRL and STR as fixed anchors: for proteins increased in STR, CTRL was set to 0 and STR to 1; for proteins decreased in STR, CTRL was 0 and STR –1. DIST and ADJ were positioned relative to this range.

Given our focus on progressive trends from CTRL to STR, we applied filtering before scaling to identify proteins consistent with an upward trend among proteins increased in STR:

- a minimum STR–CTRL difference (scaling range) of 0.3
- a minimum ADJ–CTRL difference of 0.3

In addition, proteins were excluded if they lacked progressive trends – specifically, if DIST was lower than CTRL or if DIS was higher than ADJ. Differences less than 0.15 were considered negligible (i.e., proteins where DIST–CTRL > -0.15 and DIST–ADJ < 0.15 were retained).

For proteins with decreasing trends (lower in STR), the same criteria were applied in reverse:

- a minimum CTRL–STR difference (scaling range) of 0.3
- a minimum ADJ–CTRL difference of -0.3 (i.e. ≤ -0.3)

Similarly, we excluded proteins where DIST was higher than CTRL or DIST was lower than ADJ, unless differences were minor (i.e., DIST–CTRL < 0.15 and DIST–ADJ > -0.15 were kept).

To further refine the selection to proteins to retain only those proteins consistent with a robust and progressive trend, we also included filtering for a sufficient separation between CTRL and Adj comparison, defined as a merged p-value below 0.05 in the ADJvCTRL comparison (based on integrated TMT-MS and TIMS-TOF-MS data). The final filtered subsets of proteins are displayed in panel Figure 9A and Supplemental Figure 11A (70 proteins) and Figure 10A and Supplemental Figure 11E (113 protein)

11. Additional information

Figures

The graphical abstract and schematic illustrations in Figures 1 and 8E and Supplemental Figure 1 were created using Biorender.com as detailed in the Acknowledgements in the main text, and final figures were compiled with Affinity Publisher 2.

R packages for analysis and visualization:

For data processing and analysis, the following R packages were used: Normalization (“edgeR” and “limma”), linear mixed model and differential expression (“lme4” and “emmeans”), GSEA (clusterProfiler (11)), GSVA (“GSVA” (12)) , Batch-correction (“sva” (6)), Multilevel PCA and within-matrix extraction (“mixOmcis” (8)), MCL clustering of correlation network (“MCL”), and p value merging (“ActivePathways” (5)). For visualization, most plots were generated using ggplot2. Others include Network plots (“igraph”, “ggraph”), Venn diagrams (“ggVenndiagram”), Bee swarm plots (“ggbeeswarm”), Pathway network (“enrichplot”) and Volcanoplots (“EnhancedVolcano” and “ggplot2”). Other packages used include “Uniprot.ws”.

Databases

HPA Cell markers

Cell markers used for correlation plots were obtained from the Human Protein Atlas (15, 16) website (accessed on 2024-02-06), specifically from the "Tissue Cell Type" section [https://www.proteinatlas.org/humanproteome/tissue+cell+type]. Proteins classified under the "Core Cell Type Proteome" (enriched in many, several, or some) were included for the following cell types: Endothelial cells, Smooth muscle cells, Fibroblasts, Macrophages, Neutrophils, Mast cells, T-cells and Plasma cells. Additionally, the "High" and "Very High" expression categories from the colon part of the "Tissue Cell Type Summary" were incorporated for these same cell types, as well as for Enteric glia. We excluded extracellular matrix proteins (Core Matrisome) from the cell marker list, as they can accumulate in tissue and surpass levels in the producing cells, making them unsuitable as markers on protein-level. For other cell types not listed above, we manually added cell-specific proteins when they were present in the data, for example eosinophil peroxidase and neuronal filaments.

Matrisome

The complete human matrisome protein collection (17) was downloaded from The Matrisome Project website (accessed on 2023-03-17) (Core matrisome and matrisome-associated collections) [https://sites.google.com/uic.edu/matrisome/matrisome-annotations/homo-sapiens]. Additional protein information was retrieved using the mapUniProt() function from the R package UniProt.ws, with CD antigens extracted from the protein names column.

Identification of secreted and adhesion-related proteins

Matrisome information was accessed as described above. Ligand-receptor interaction data were obtained from CellTalkDB (18) (human_lr_pair.txt downloaded 2024-10-17 from https://xomics.com.cn/celltalkdb/download.php). Keywords were retrieved from Uniprot accessed via R using the Uniprot.ws package. Proteins with at least one of keywords “Secreted”, “Cell adhesion”, “Receptor” or “Cytokine” were further investigated. Functional keyword annotations were retrieved from UniProt using the Uniprot.ws R package. Proteins annotated with at least one of the keywords “Secreted”, “Cell adhesion”, “Receptor” or “Cytokine” were retained for further analysis. Data from the Matrisome database, CellTalkDB and UniProt keyword searches were integrated. Granulocyte granule proteins were manually added to the dataset. Intracellular receptors, intracellular ligand-like molecules (non-adhesion membrane receptors) and immunoglobulins were excluded. The final curated list is presented in Supplemental Table 5 (SM) and Supplemental Table 11 (MP). For correlation analysis between mapped proteins and top DE proteins (Supplemental Figure 9), we included only proteins that met one of the following criteria:

- High WE: top 25% increased in the SM layer or top increased 33% in the MP layer, or
- Lower WE but displaying DE in both TMT-MS and TIMS-TOF-MS datasets.

This filtering approach aimed to prioritize robust, biologically meaningful signals.

Supplementary Results

1. Parallel TIMS-TOF-MS corroborates differential protein expression trends obtained using TMT-MS analysis

Parallel analysis of the same 64 samples using label-free TIMS-TOF-MS (Figure 1C) identified a total number of proteins comparable to the TMT-MS data, despite using only one-tenth of the sample input (Supplemental Figure 2, G and H). In both tissue layers, TIMS-TOF-MS detected approximately 70% of the proteins quantified by TMT-MS in the corresponding layer and additionally quantified more than 800 proteins per layer that were not identified by TMT-MS.

Similar to TMT-MS, PCA of the TIMS-TOF-MS data revealed separation between STR and CTRL within a tissue layer along PC1 (SM, Supplemental Figure 2I) and PC2 (MP, Supplemental Figure 2J). The number of DE proteins in SM and MP using TIMS-TOF-MS was lower for STRvNSTR and STRvCTRL compared to TMT-MS (Supplemental Figure 2, E, F, K and L). Overall, despite using 1/10th of the same input material, TIMS-TOF-MS demonstrated consistency with the global trends observed in the TMT-MS data and identified novel proteins not found with TMT-MS.

2. Pathway enrichment supports SM stricture shifts from homeostasis toward anabolism with enhanced secretory and immune activities

Consistent with the observations in the protein marker data, parallel gene set enrichment analyses (GSEA) showed enriched pathways clustered into distinct biological themes (Supplemental Figure 3, C and D). Negative enrichment of pathways was related to neuronal processes (pink), hemostasis/complement (blue), heme metabolism and oxygen transport (blue) and ECM organization (orange) (Supplemental Figure 3, C and D; Supplemental Table 3). This is consistent with negative association of enteric glia cell, endothelial cell, red blood cell and many matrisome proteins with PC1 (Figure 2B). The negative enrichment of heme and hemostasis/complement pathways (Supplemental Figure 3D) was driven by marked reduction in many blood and plasma-related proteins (Supplemental Table 3), suggesting a broader decrease in blood-related proteins in STR SM. Key proteins contributing to the negative enrichment of ECM organization pathways included structural ECM components mentioned earlier (Supplemental Table 2), but also proteins associated with platelet function, basement membrane structure, cell junctions and matrix degradation (Supplemental Table 3). In addition, many metabolism-related

pathways exhibited negative enrichment (Supplemental Figure 3, C and D). Overall, pathways related to structural or functional homeostasis were negatively enriched in STR SM.

In contrast to negative enrichment of homeostatic functions, immune response pathways were positively enriched (Supplemental Figure 3, C and D, green; Supplemental Table 3). This is consistent with increased pro-inflammatory immune markers. GSEA also highlighted that the most positively enriched pathways in SM were related to cell cycle, stress response and protein synthesis (Supplemental Figure 3C) with notable examples including MYC targets and the unfolded protein response. Several proteins involved in these pathways were overrepresented among the top contributors to PC1, reflecting their strong association with this axis (Supplemental Figure 3E; Supplemental Table 3).

3. Mitochondrial metabolism pathways are reduced while immune-related processes are increased in the MP of strictured tissue

Negative enrichment of pathways primarily related to cellular respiration and metabolism characterized STR MP (Supplemental Figure 4, B and C), with the majority of top negative loadings of PC1 (reduced in STR) being linked to metabolism and mitochondria (Supplemental Figure 4A). This suggests a relative decrease in cellular respiration accompanied the reduction in SMC markers. In contrast, immune response pathways showed prominent positive enrichment (Supplemental Figure 4, B and C), consistent with the relative increase in immune cell markers (Figure 3B). Other prominent pathway themes increased in MP STR included gene expression regulation and RNA and protein metabolism (Supplemental Figure 4, B and C). However, the fold changes of RNA and protein metabolism were relatively subtle in MP and only a fraction of core proteins of these pathways were DE.

4. Integration of TMT-MS and TIMS-TOF-MS datasets

To enhance analysis robustness, we integrated DE results from both the TMT-MS and TIMS-TOF-MS datasets for each layer and both directions (increased and decreased relative abundance) and complemented this analysis with STRvDist and STRvAdj comparisons. The combined-level proteins identified in each comparison in SM (Supplemental Figure 6, A–D) resulted in a union of 479 proteins in the positive direction (Supplemental Figure 6F) and 566 proteins in the negative direction (Supplemental Figure 6G) for further analysis. In both directions, the highest number of DE proteins was observed in STRvCTRL, followed by STRvNSTR, STRvDist, and the fewest in

STRvAdj (Supplemental Figure 6, F and G). This is consistent with the trend observed in Supplemental Figure 5. For the MP layer (Supplemental Figure 7, A–D), 267 proteins were identified in both the positive (Supplemental Figure 7F) and negative (Supplemental Figure 7G) directions at the combined level. Distinct from the SM layer, the highest number of DE proteins in MP, in both directions, was observed in the STRvNSTR comparison, followed by STRvCTRL, STRvDist and STRvAdj (Supplemental Figure 7, F and G). Overall, integrating the TMT-MS and TIMS-TOF MS results increases robustness of DE analysis, and served as the foundation for subsequent downstream analyses.

5. Intercorrelated, highly DE secreted and adhesion proteins reflect parallel processes occurring in STR SM

We next performed correlation network clustering on all DE proteins increased in STR SM (Supplementary Methods 10). This revealed one large cluster (Cluster 32) and many smaller, interconnected clusters (Supplemental Figure 9A). Cluster 32 included several already mentioned DE proteins (see main text) including the secreted glycoprotein CTHRC1 and the immunity-related secreted proteins IL16 and MZB1 (Supplemental Figure 9B). Leukocyte adhesion proteins, and the majority of top (intracellular) DE proteins related to immunity and ER, such as TXNDC5, were also in Cluster 32. The high intercorrelation between the proteins in this large cluster suggest common involvement in processes occurring in the SM layer of STR tissue.

Notably, beyond Cluster 32, which included the ECM protein CTHRC1, most other ECM proteins were found in several smaller, ECM-enriched interconnected clusters (Supplemental Figure 9C). These clusters included the top ECM protein TNC, along with the collagen cross-linking enzyme LOXL1, that were highly interconnected with other ECM proteins. Moreover, the distribution of integrin ITGA8, several cytokines and top DE intracellular (non-ECM) proteins (GUCY1A1, BASP1, CNN2) within this domain suggest these proteins follow restructuring in the ECM. It was also noteworthy that the leukocyte adhesion proteins ADGRE5 and ICAM3 (Supplemental Figure 9C, green) exhibited strong correlations with ECM proteins, unlike other DE leukocyte adhesion molecules in STR SM (Supplemental Figure 9B, green). In summary, a large cluster enriched in immunity- and ER proteins, and other smaller interconnected clusters enriched in ECM proteins, characterize the top DE proteins in STR SM. These clusters were associated with a distinct set of secreted ECM proteins, ligands, adhesion molecules and intracellular proteins, potentially reflecting distinct yet parallel processes occurring in STR SM tissue.

6. Network clustering reveals interrelated DE proteins potentially reflecting distinct yet parallel processes occurring in STR MP

Similar to SM, we contextualized the DE proteins increased in STR MP using correlation network clustering. This revealed two large and closely associated clusters (Clusters 11 and 29) that, similar to the SM layer, included immunity and ER-related proteins such as MZB1 and TXNDC5, and proteins involved in leukocyte adhesion (Supplemental Figure 9, D and E). These two clusters also contained a subset of granule proteins. The remainder of the network comprised many smaller interconnected clusters. Part of this subnetwork was enriched in ECM and mast cell clusters and revealed high intercorrelation between the top increased DE proteins POSTN (red) and CPA3 (orange) (Supplemental Figure 9F). The majority of the intercorrelated white nodes were related to RNA and nuclear activities. In addition, several intracellular proteins correlated to ECM and mast cells, including FUBP1 and NCL, which are associated with activities in growing or proliferating cells. Taken together, the network analysis of DE proteins increased in STR MP revealed strong correlation between mast cell proteins, ECM proteins and key intracellular proteins, suggesting a shared expression profile distinct from that of other DE proteins.

7. Interlayer analysis highlights protein categories with shared expression in the SM and MP layers

Our goal was to explore shared signatures across the SM and MP layers in STR tissue. To characterize the intersecting DE proteins between the SM and MP layers identified through our interlayer analysis, we visualized PPI networks for the four shared DE groups (see also Figure 8). Among concordantly increased proteins in both layers, 120 were identified, primarily associated with immunity and broad intracellular processes including mRNA-, nuclear- and protein activities (Supplemental Figure 10A). Proteins related to immunity and ER chaperone function, such as MZB1 and TNDC5, displayed the largest WE in both layers (Supplemental Figure 10, A and B). In contrast, only 53 DE proteins showed concordant decreases in both layers, forming a heterogeneous mix of protein clusters where half were associated with metabolic processes (Supplemental Figure 10C). Several lipid transporters, particularly FABP1 and FABP6, exhibited pronounced negative WE in both layers as did erythrocyte- or blood vessel-related proteins, particularly in the SM (Supplemental Figure 10, C and D). This highlights intersections among otherwise distinct sets of negative DE proteins in SM and MP.

In contrast to DE proteins concordantly altered in STR SM and MP, a subset of proteins changed in opposing directions across the layers (see Figure 8, blue). Proteins with a relative increase in SM but decrease in MP included several associated with smooth muscle and TCA cycle (Supplemental Figure 10E) and the protein BASP1, which was the only protein ranked among the Top 30 in opposite directions between SM and MP (see Figure 8D, blue). Conversely, proteins that exhibited a relative increase in MP but decrease in SM included the mast cell granule proteins CPA3 and CMA1, macrophage-associated proteins, neuronal adhesion molecules and ECM proteins (Supplemental Figure 10F).

To summarize, DE proteins with concordant expression changes across layers were preferentially observed in the positive direction, particularly those involved in immunity and ER processing (MZB1, TXNDC5). Fewer proteins showed concordant decreases, with prominent decreases mainly related to lipid metabolism (FABP1, FABP6) and vascular components. Moreover, a subset of DE proteins has discordant changes in the SM and MP, suggesting parallel processes occur in different directions in SM and MP.

References for Supplemental information

1. Friedrich C, et al. Comprehensive micro-scaled proteome and phosphoproteome characterization of archived retrospective cancer repositories. *Nat Commun*. 2021;12(1).
2. Yi L, et al. Boosting to Amplify Signal with Isobaric Labeling (BASIL) Strategy for Comprehensive Quantitative Phosphoproteomic Characterization of Small Populations of Cells. *Anal Chem*. 2019;91(9):5794-801.
3. Skowronek P, et al. Rapid and In-Depth Coverage of the (Phospho-)Proteome With Deep Libraries and Optimal Window Design for dia-PASEF. *Mol Cell Proteomics*. 2022;21(9):100279.
4. Plubell DL, et al. Extended Multiplexing of Tandem Mass Tags (TMT) Labeling Reveals Age and High Fat Diet Specific Proteome Changes in Mouse Epididymal Adipose Tissue. *Mol Cell Proteomics*. 2017;16(5):873-90.
5. Slobodyanyuk M, et al. Directional integration and pathway enrichment analysis for multi-omics data. *Nat Commun*. 2024;15(1):5690.
6. Leek JT, et al. The sva package for removing batch effects and other unwanted variation in high-throughput experiments. *Bioinformatics*. 2012;28(6):882-3.
7. González I, et al. Visualising associations between paired 'omics' data sets. *BioData Min*. 2012;5(1):19.
8. Rohart F, et al. mixOmics: An R package for 'omics feature selection and multiple data integration. *PLoS Comput Biol*. 2017;13(11):e1005752.
9. Liberzon A, et al. The Molecular Signatures Database (MSigDB) hallmark gene set collection. *Cell systems*. 2015;1(6):417-25.
10. Milacic M, et al. The Reactome Pathway Knowledgebase 2024. *Nucleic Acids Res*. 2023;52(D1):D672-D8.
11. Wu T, et al. clusterProfiler 4.0: A universal enrichment tool for interpreting omics data. *The Innovation*. 2021;2(3):100141.
12. Hänzelmann S, et al. GSVA: gene set variation analysis for microarray and RNA-seq data. *BMC Bioinformatics*. 2013;14:7.
13. Szklarczyk D, et al. The STRING database in 2023: protein-protein association networks and functional enrichment analyses for any sequenced genome of interest. *Nucleic Acids Res*. 2023;51(D1):D638-d46.
14. Liquet B, et al. A novel approach for biomarker selection and the integration of repeated measures experiments from two assays. *BMC Bioinformatics*. 2012;13(1):325.
15. Dusart P, et al. A tissue centric atlas of cell type transcriptome enrichment signatures. *bioRxiv*. 2023:2023.01.10.520698.
16. Uhlén M, et al. Tissue-based map of the human proteome. *Science*. 2015;347(6220):1260419.
17. Naba A, et al. The matrisome: in silico definition and in vivo characterization by proteomics of normal and tumor extracellular matrices. *Mol Cell Proteomics*. 2012;11(4):M111.014647.
18. Shao X, et al. CellTalkDB: a manually curated database of ligand–receptor interactions in humans and mice. *Brief Bioinform*. 2021;22(4).

Supplemental Figures 1–11

Supplemental Figure 1. Overview of patients and tissues....26

Supplemental Figure 2. Global assessment of TMT-MS and TIMS-TOF-MS data reveals proteome alterations in SM and MP of strictures compared to control tissues....28

Supplemental Figure 3. Extended PCA and GSEA analysis of the SM layer....30

Supplemental Figure 4. Extended PCA and GSEA analysis of the MP layer....32

Supplemental Figure 5. PCA with Adj and Dist nonstrictured tissue reveals progressive changes with relative distance to strictures....34

Supplemental Figure 6. Integration of DE proteins from the TMT-MS and TIMS-TOF-MS data for the SM layer....35

Supplemental Figure 7. Integration of DE proteins from the TMT-MS and TIMS-TOF-MS data for the MP layer....37

Supplemental Figure 8. Protein categories among combined-level DE proteins....39

Supplemental Figure 9. Correlation-based network clustering and ECM domain associations....41

Supplemental Figure 10. Extended interlayer analysis....43

Supplemental Figure 11. Extended analysis of progressive protein expression trends....45

Supplemental Figure 1

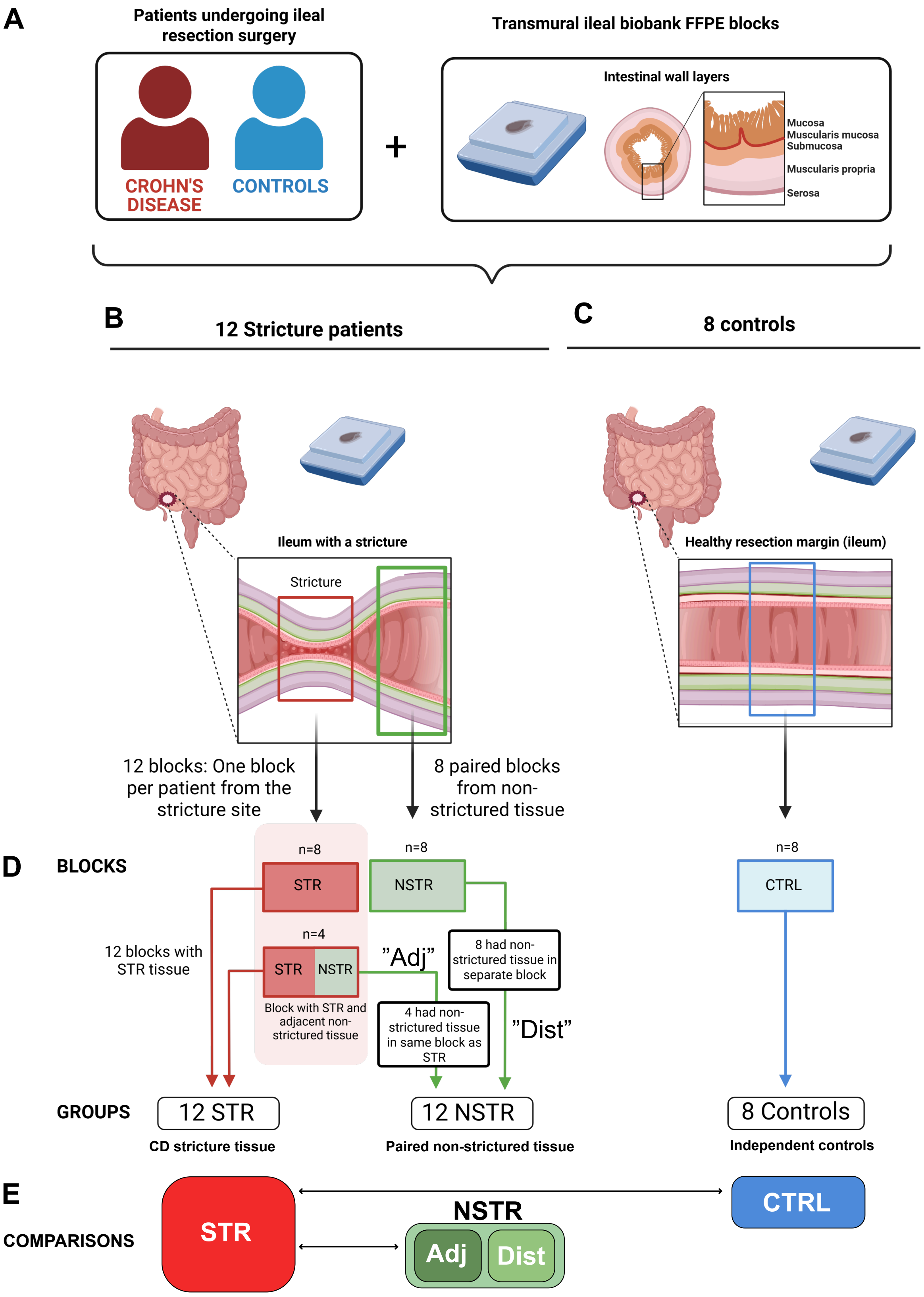

Supplemental Figure 1. Overview of patients and tissues. (A) Ileal tissue from 12 CD stricture patients and 8 independent controls was surgically resected and stored in the Sahlgrenska University Hospital biobank as FFPE blocks. **(B** and **D)** show an overview of the location of stricture (STR) tissue and paired non-stricture tissue (NSTR) tissue with the adjacent (Adj) and distal (Dist) location of NSTR relative to STR indicated in **(D)**. **(C)** indicates the ileal tissue from healthy resection margin of colorectal cancer patients undergoing ileal resection that were used as independent control (CTRL) tissue. Of the 12 CD patients with FFPE blocks of STR and NSTR tissue, four had FFPE blocks in the biobank that contained both STR and NSTR in the same contiguous segment and block as shown to the left in **(D)**; this NSTR tissue is called adjacent (Adj) in the text. Eight CD patients had NSTR tissue in an FFPE block separate from that containing their STR tissue (but with both from the same surgical procedure) as indicated to the right in **(D)**; this NSTR tissue is called distal (Dist) in the text. **(E)** Shows an overview of the comparisons used for differential expression analysis of the microdissected tissue layers. The figure was created with BioRender.com

Supplemental Figure 2

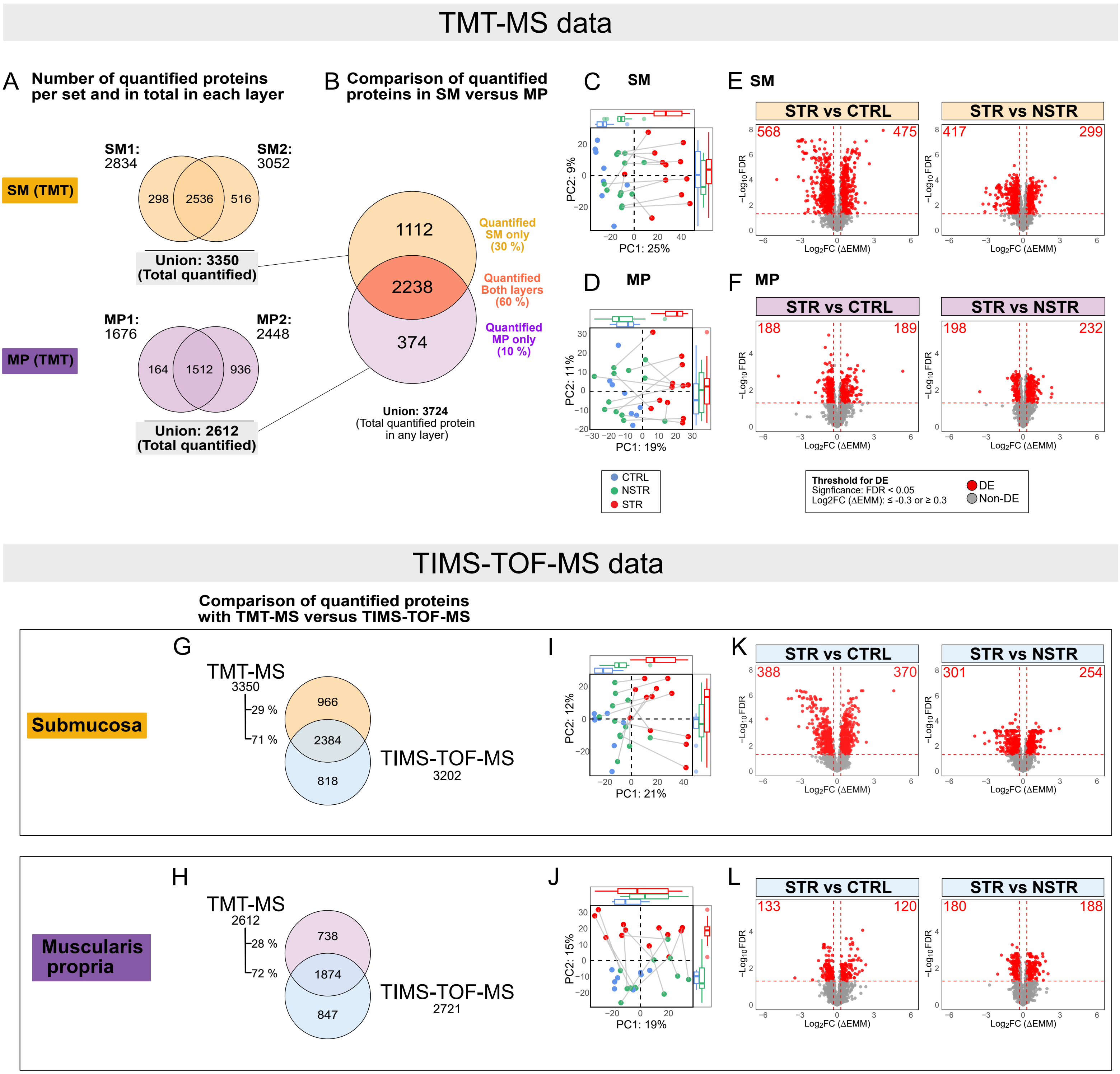

Supplemental Figure 2. Global assessment of TMT-MS and TIMS-TOF-MS data reveals proteome alterations in SM and MP of strictures compared to control tissues. (A–F) summarize TMT-MS data while **(G–L)** summarize parallel, label-free TIMS-TOF-MS data from an aliquot (1/10th) of the same microdissected tissue samples. **(A–F)** show the number of proteins quantified **(A** and **B)**, PCA analysis **(C** and **D)** and differential expression (DE) **(E** and **F)** of TMT-MS data. Data from the submucosa (SM) layer are indicated in yellow and from the muscularis propria (MP) layer in purple. **(A** and **B)** As the number of samples to be analyzed exceeded the number of unique barcodes (TMT labels) available, samples were analyzed as two separate TMT sets of 16 samples per layer (SM1 and SM2 in yellow and MP1 and MP2 in purple; see Figure 1C and Supplementary Methods 6 and 7). **(A)** Venn diagrams show the number of proteins quantified per TMT set in each layer (SM1, SM2, MP1, MP2). The intersecting regions (e.g. 2536 proteins in the SM layer) represent proteins quantified in both TMT sets, corresponding to all 32 samples. The non-intersecting regions (e.g. 298 in SM1 and 516 in SM2) indicate proteins quantified in only one of the TMT sets, corresponding to 16 samples. The union of the TMT sets (e.g. 3350 proteins in the SM layer) represents all proteins quantified in that layer. This corresponds to the number of rows (proteins) in the layer-wise TMT-MS datasets (i.e., relative expression data across 16 to 32 samples) used for downstream analysis. **(B)** Venn diagram comparing the overlap of all proteins [the two unions in **(A)**] quantified in SM versus MP, as indicated. **(C** and **D)** PCA plot (PC1 vs PC2) with axis PC score boxplots for batch-corrected with complete observations for **(C)** SM and **(D)** MP. The percentage in the axis titles indicates the proportion of variance explained by each PC. Samples are color-coded by tissue type as indicated under **(D)**. STR and NSTR samples from the same individual are connected by lines. Axis boxplots show the median (center line), interquartile range (box), with whiskers to the most extreme values within 1.5 x IQR, and outliers plotted as individual points. **(E** and **F)** Volcano plots of the stricture (STR) vs paired non-stricture (NSTR) and STR vs independent control (CTRL) comparisons for **(E)** SM and **(F)** MP. The number of DE proteins per direction are indicated in red. DE was tested using linear mixed-effects models with model-based contrasts of estimated marginal means (EMM), with degrees of freedom estimated using the Kenward-Roger method; p values were adjusted for multiple testing using the Benjamini–Hochberg method to control the false discovery rate (FDR). **(G–L)** show the initial global assessment of the TIMS-TOF-MS data from the SM and MP aliquots removed before TMT labeling; see also Figure 1C. **(G** and **H)** show the number of quantified proteins, **(I** and **J)** show PCA analysis while **(K** and **L)** are DE analysis. **(G** and **H)** display the number of proteins quantified in the SM and MP TIMS-TOF-MS datasets in relation to the corresponding TMT-MS dataset (the unions in **A**) in each layer. The percentages to the left are relative to all proteins quantified with TMT-MS (i.e. in both layers about 70 % of TMT-MS proteins were also quantified with TIMS-TOF-MS, despite using only 1/10th of the same sample for TIMS-TOF-MS). Note that the number of quantified proteins with TIMS-TOF-MS refers to those included in the merged results table (Supplementary methods 9). **(I–L)** correspond to **(C–F)** but display results from TIMS-TOF-MS data.

Supplemental Figure 3

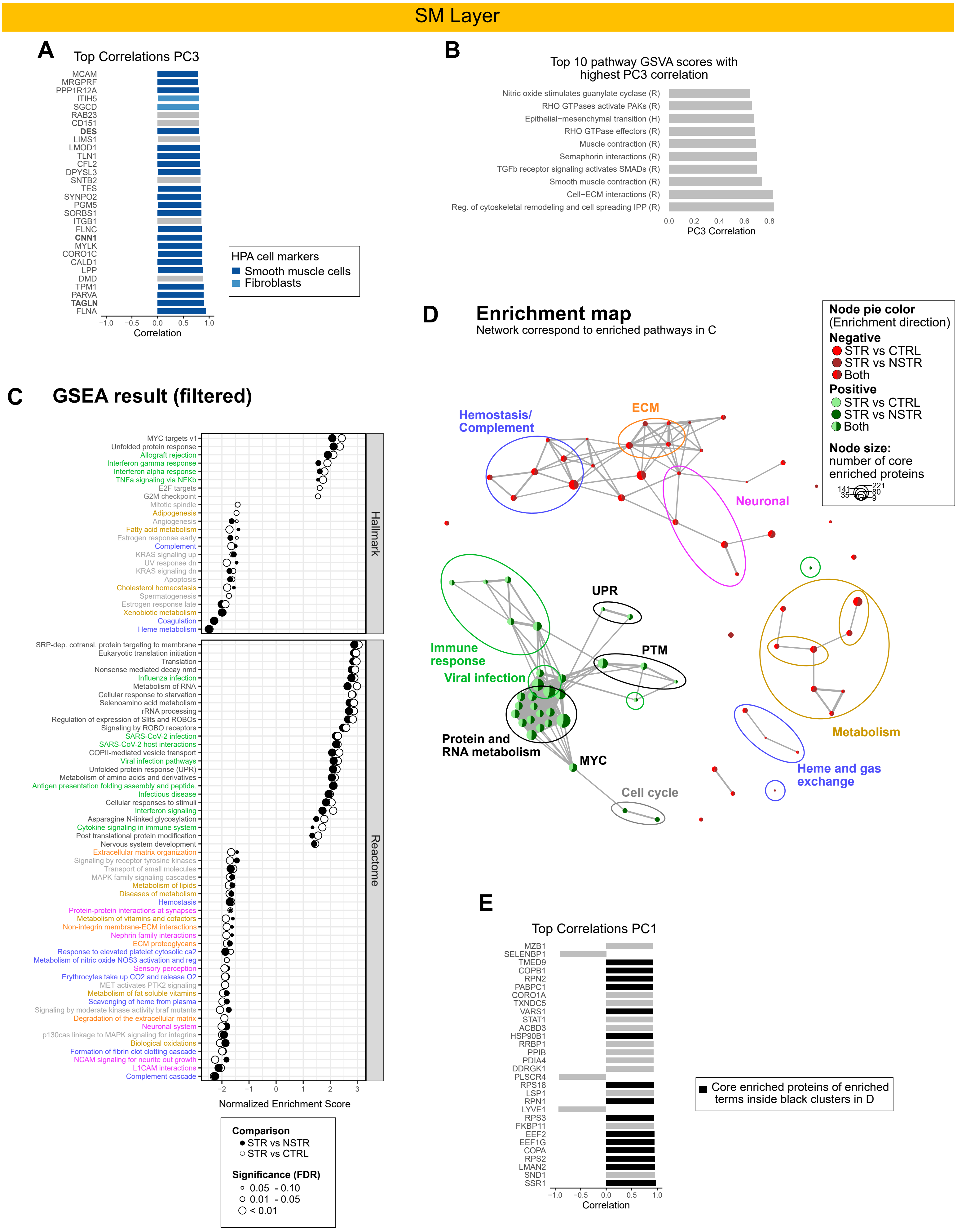

Supplemental Figure 3. Extended PCA and GSEA analysis of the SM layer. This figure shows complementary information to PCA in Figure 2 and summarizes the Gene (Protein) Set enrichment analysis (GSEA) in the SM layer. **(A)** Bar plot displaying the top 30 proteins with the highest absolute correlation to PC3, arranged in decreasing order from bottom to top. Bars are colored according to HPA Cell marker category (as in Figure 2, B and C) and the proteins in bold are markers labeled in Figure 2B. **(B)** Bar plot showing Hallmark (H) and Reactome (R) pathways Gene Set Variation Analysis (GSVA) scores most strongly correlated with PC3, arranged in decreasing order from bottom to top. **(C** and **D)** Results of GSEA in the SM layer displaying the list of significantly enriched pathways after redundancy filtering (see Supplementary Methods 10). **(C)** Dot plot of normalized enrichment scores for enriched pathways. The terms in this plot correspond to nodes in, and are colored according to, clusters in **(D)**. Dot filling indicates the comparison, and dot size reflects significance, as indicated. **(D)** Enrichment map visualizing enriched pathways (nodes) and their overlap (edges) based on core enriched proteins, along with identified biological themes. Different clusters are indicated in different colors and are consistent with the term color in **(C)**. **(E)** Bar plots displaying the top 30 proteins with the highest absolute correlation for PC1, arranged in decreasing order from bottom to top. Proteins representing core enriched proteins of the black clusters in D are shown in black. Correlation coefficients in panels A, B, and E refer to Pearson correlation coefficients.

Supplemental Figure 4

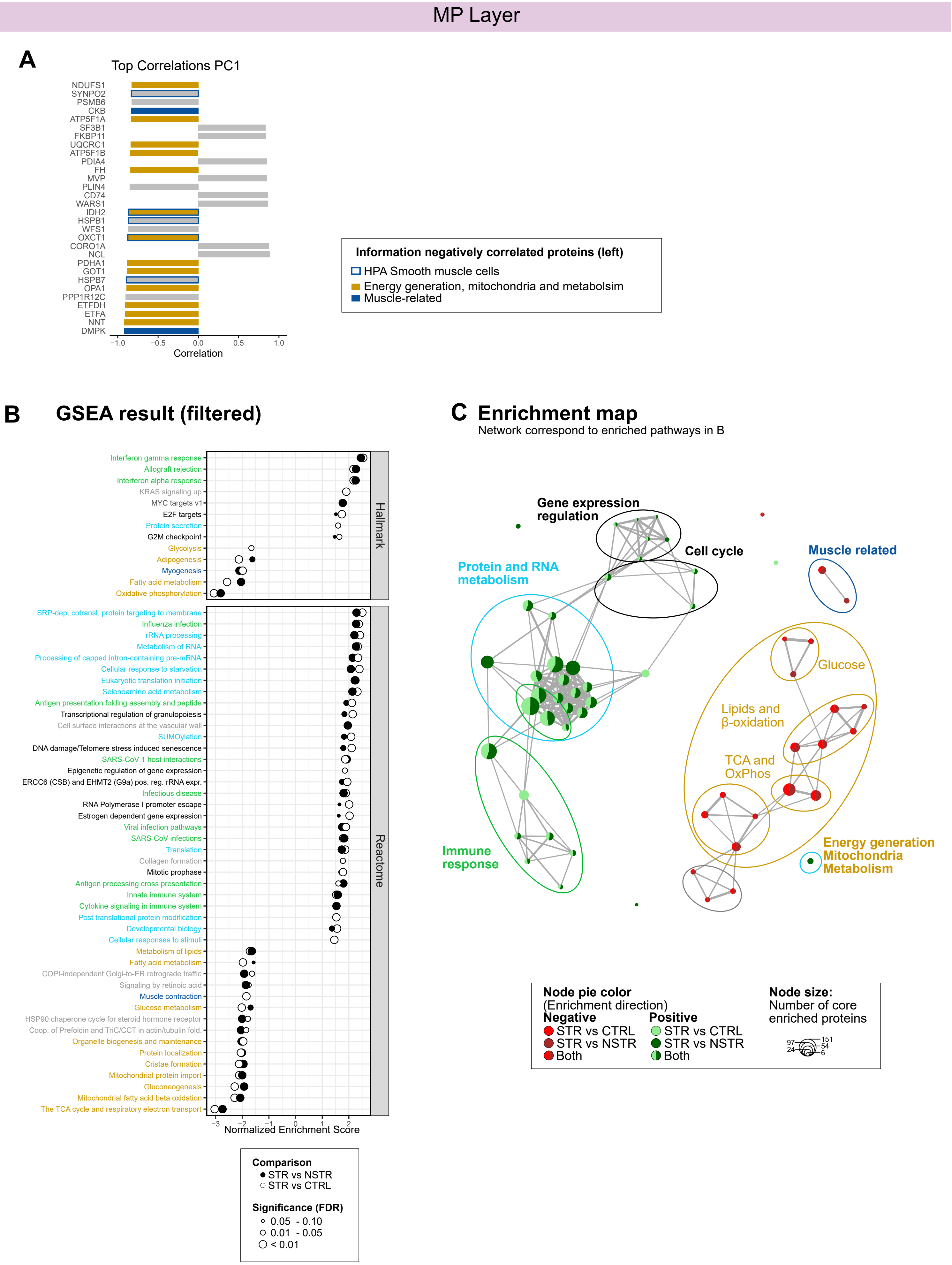

Supplemental Figure 4. Extended PCA and GSEA analysis of the MP layer. This figure shows complementary information to PCA in Figure 3 and summarizes the Gene (Protein) Set enrichment analysis (GSEA) in the MP layer. **(A)** Bar plot displays the top 30 proteins with the highest absolute Pearson correlations for PC1 arranged in decreasing order from bottom to top. Bars on the left side (negatively correlated to PC1) are colored according to pathway cluster in (**B** and **C**) and HPA Cell marker (as in Figure 3, B and C). Unlike the SM layer (Figure 3B; Supplementary Figure 3A), many markers in the MP had generally low correlation with PC3 and are thus not listed as for the SM in Supplemental Figure 3. **(B** and **C)** corresponds to Supplemental Figure 4, C and D but for the MP layer. See the legend to Supplemental Figure 3 for details.

Supplemental Figure 5

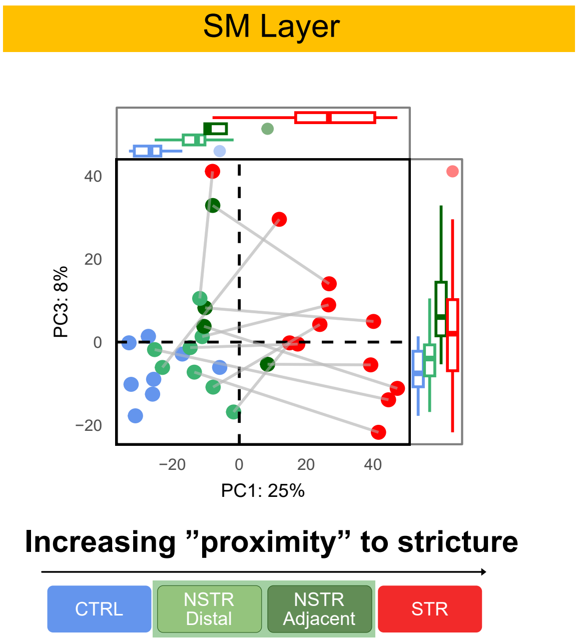

Supplemental Figure 5. PCA with Adj and Dist nonstrictured tissue reveals progressive changes with relative distance to strictures. The figure shows a refined version of the PCA plot in **Figure 2A** distinguishing NSTR adjacent (Adj) and NSTR distal (Dist) samples (see **Supplemental Figure 1**). The schematic below the plot illustrates the proximity concept discussed in the text and provides the color key corresponding to the samples (circles) in the plot. Axis boxplots show the median (center line), interquartile range (box), whiskers extending to the smallest and largest values within 1.5 x the interquartile range, and outliers plotted as individual points.

Supplemental Figure 6

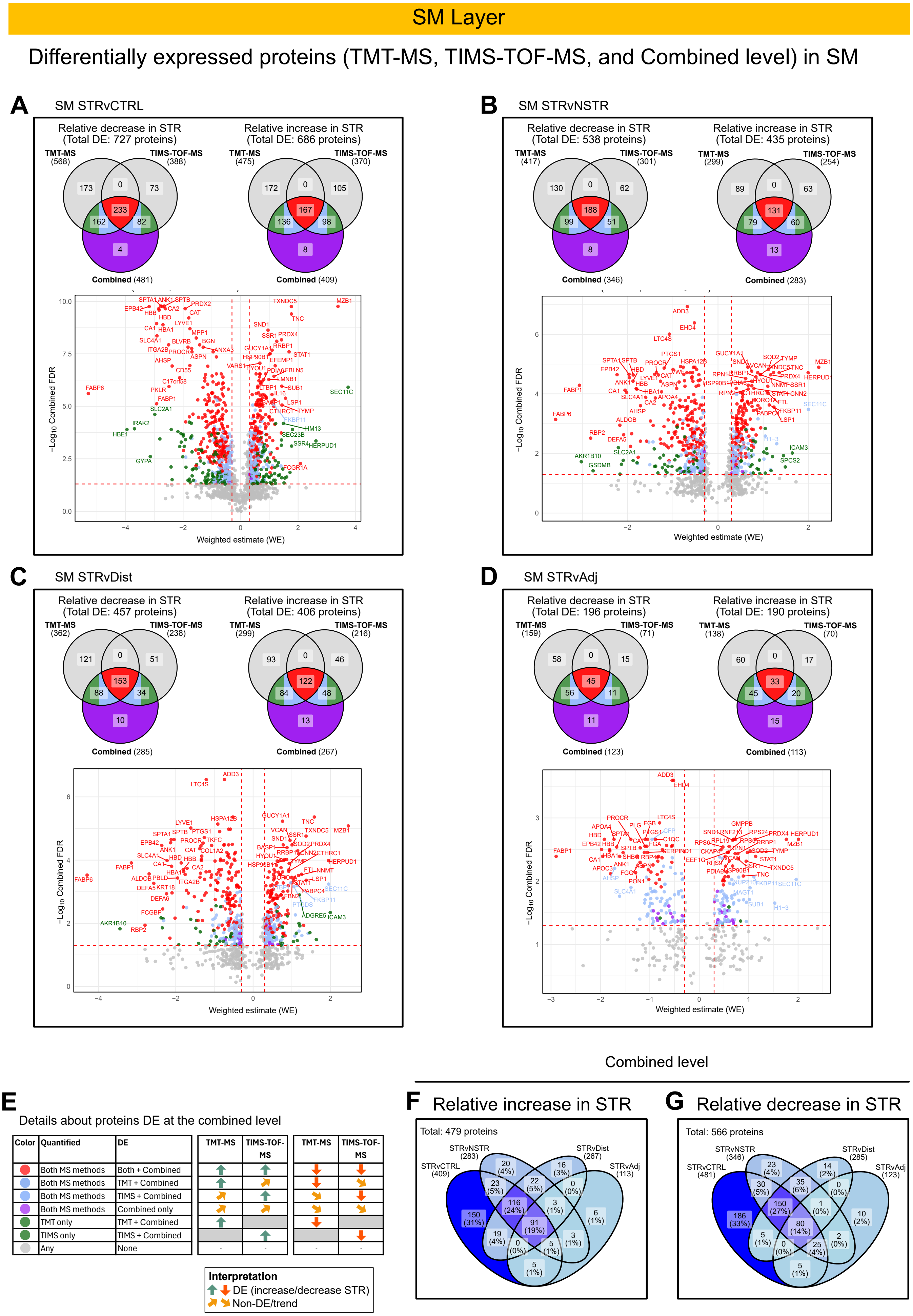

Supplemental Figure 6. Integration of DE proteins from the TMT-MS and TIMS-TOF-MS data for the SM layer. (A–D) show the number of proteins quantified with a relative increase or decrease in the SM layer of STR for the TMT-MS, TIMS-TOF-MS and combined level (see Supplementary Methods 9), as well as the dataset intersections. Each panel in **(A–D)** represents a specific comparison and includes two Venn diagrams (one for each direction of change, relatively decreased or increased) with a volcano plot of the combined-level metrics [Weighted estimate (WE) on the x-axis and $-\log_{10}$ FDR adjusted merged p-value on the y-axis]. In each Venn diagram, the upper left circle ("TMT-MS") indicates the number of differentially expressed (DE) proteins identified in the TMT-MS data, while the upper right circle ("TIMS-TOF-MS") shows the corresponding number from the TIMS-TOF-MS data. The bottom circle (Combined) represents proteins identified as DE at the combined level (see Supplementary Methods 9). Color coding in **(E)** reflects the level of evidence and the sources of combined level proteins and aligns with the colors in the Venn diagrams and the dot colors in the corresponding volcano plots. Proteins classified as DE at the combined level are color-coded as follows: **Red** indicates proteins DE in both TMT-MS and TIMS-TOF-MS and are present in the intersection of all three circles as in **(A–D)**. **Blue** represents proteins quantified by both TMT-MS and TIMS-TOF-MS but DE in only one, with sufficient signal to be considered DE at the combined level. **Green** denotes proteins quantified by only one method (either TMT-MS or TIMS-TOF-MS), but with strong enough DE to meet the combined-level threshold. **Purple** indicates the few proteins that were DE at the combined level only. **Grey** are proteins that do not meet the combined-level threshold (of WE > 0.3 or < –0.3 and FDR-adjusted merged p-value < 0.05).

Additional details

Venn diagrams: For the **non-red** overlaps between one method and the combined level (e.g., TMT-MS and combined level, but not TIMS-TOF-MS; or TIMS-TOF-MS and combined level, but not TMT-MS), the intersection has been split into two segments to emphasize that two different types of DE proteins (**blue** and **green**, see above) contribute to the number given in the intersection. **(F and G)** show the intersections among all proteins identified as DE at the combined level per direction where each ellipse corresponds to the lower circles (Combined) in the Venn diagram in the comparisons in **(A–D)**. The union formed by the four sets in each direction in **(F and G)** include a total of 479 DE proteins with a relative increase **(F)** in abundance and a total of 566 DE proteins with a relative decrease **(G)** in abundance was used for downstream analysis.
Volcano plots: The Top 30 Ranked DE proteins in each comparison are labeled. Note the distribution of the different colors (**red** has the highest significance, then **blue** and **green**).

Supplemental Figure 7

MP Layer

Differentially expressed proteins (TMT-MS, TIMS-TOF-MS, and Combined level) in MP

A MP STRvCTRL

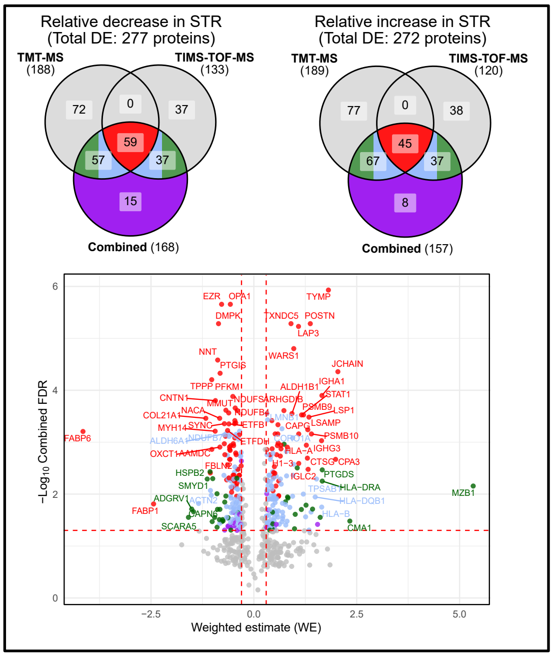

B MP STRvNSTR

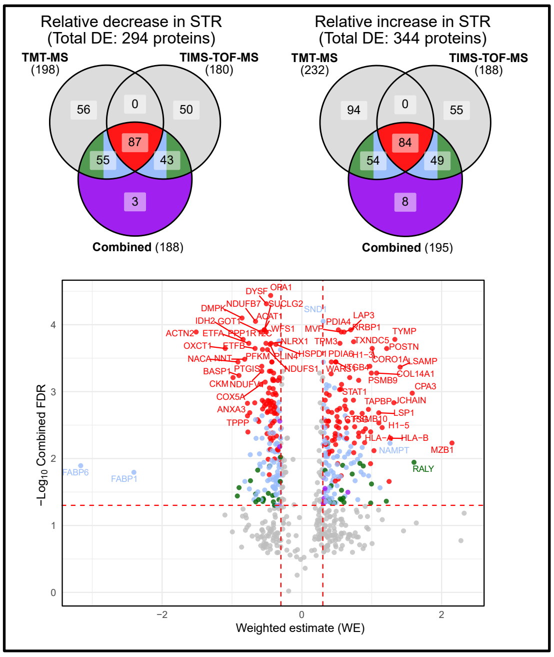

C MP STRvDist

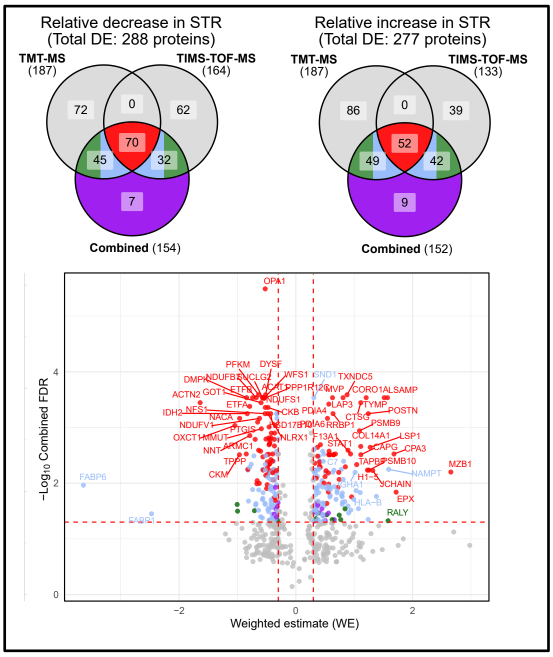

D MP STRvAdj

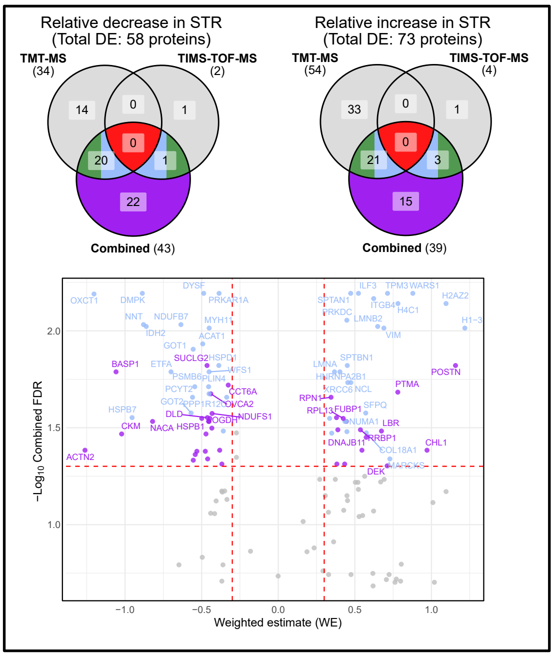

E Details about proteins DE at the combined level

Color	Quantified	DE	TMT-MS	TIMS-TOF-MS	TMT-MS	TIMS-TOF-MS
●	Both MS methods	Both + Combined	↑	↑	↓	↓
●	Both MS methods	TMT + Combined	↑	↗	↓	↘
●	Both MS methods	TIMS + Combined	↗	↑	↘	↓
●	Both MS methods	Combined only	↗	↗	↘	↘
●	TMT only	TMT + Combined	↑		↓	
●	TIMS only	TIMS + Combined		↑		↓
●	Any	None	-	-	-	-

Interpretation
↑↓ DE (increase/decrease STR)
↗↘ Non-DE/trend

Combined level

F Relative increase in STR

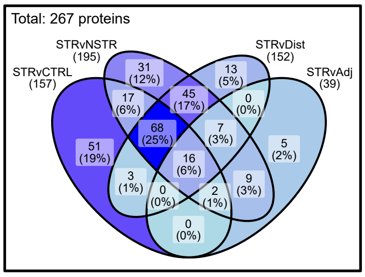

G Relative decrease in STR

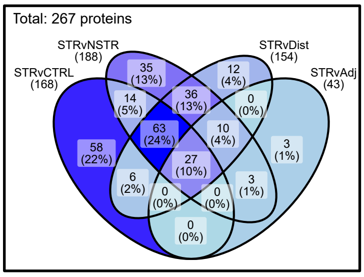

Supplemental Figure 7. Integration of DE proteins from the TMT-MS and TIMS-TOF-MS data for the MP layer. This figure parallels Supplemental Figure 6 except the data are for the MP layer. See the legend to Supplemental Figure 6 for details.

Supplemental Figure 8

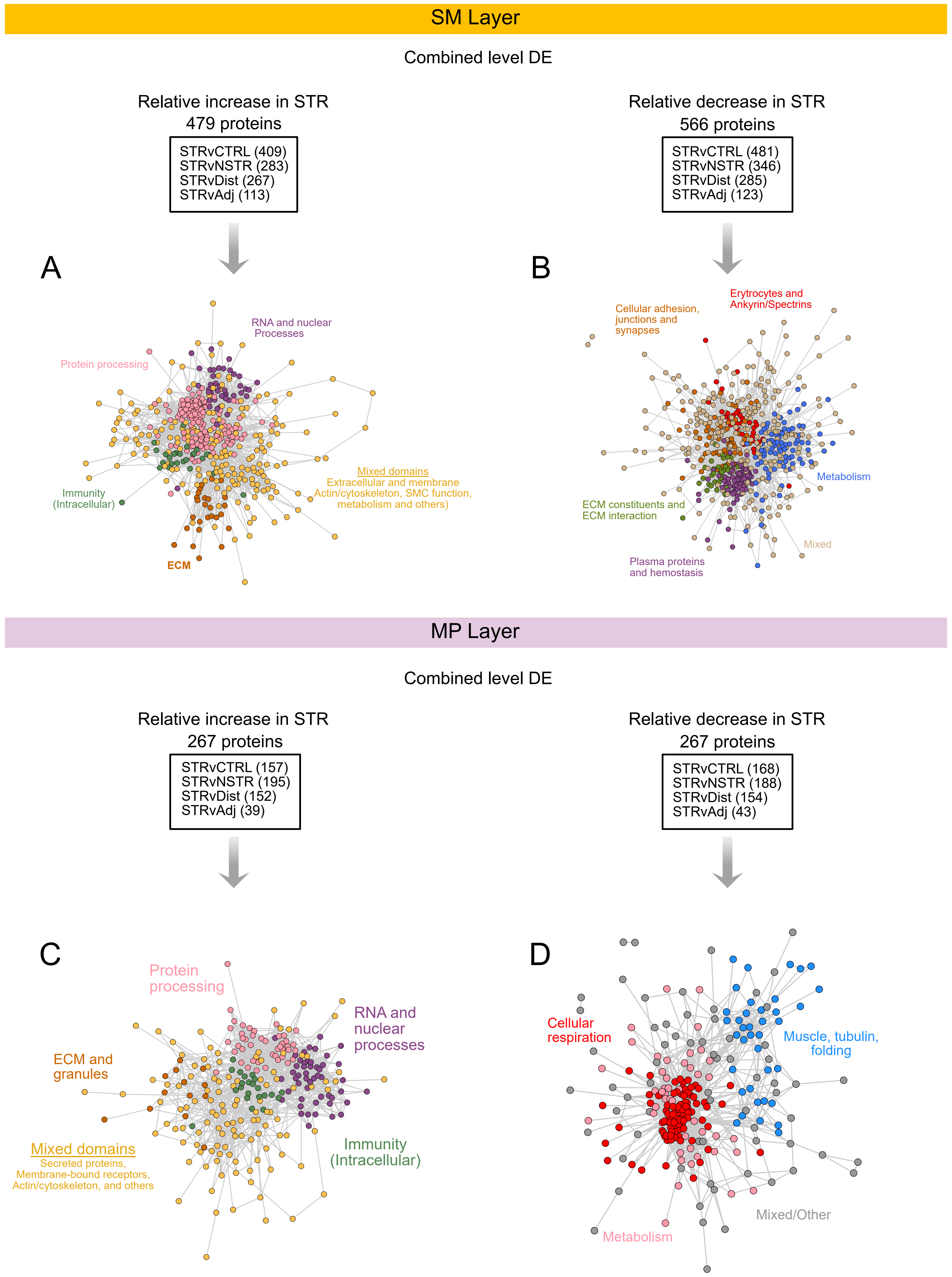

Supplemental Figure 8. Protein categories among combined-level DE proteins. This figure shows the four STRING protein–protein interaction networks formed by all DE proteins (nodes) in each layer and direction of DE and provide an overview of broad protein categories these proteins mapped to. (**A** and **B**) corresponds to unions in (Supplemental Figure 6, F and G) and (**C** and **D**) to the unions in (Supplemental Figure 7, F and G). Edges indicate interaction scores ≥ 0.4. Network clustering was applied to identify functionally related groups, which were subsequently organized into broader subdomains, color-coded and given a general umbrella term capturing the overarching functional themes (see Supplementary Methods 10). Note that the functional protein categories highly represented in the interaction network are consistent with the results of PCA and GSEA analyses of the TMT-MS data of each layer (SM, Figure 2B and Supplementary Figure 3, B and C; MP, Figure 3B and Supplementary Figure 4, B and C). In **A** and **C**, the green category only includes intracellular protein linked to immune processes while secreted, membrane-bound proteins, and other immune-related proteins such as those involved in mobility/cytoskeleton are in the mixed (orange) domain.

Supplemental Figure 9

Relative increase in STR SM layer

A **SM Meta network of correlation clusters**

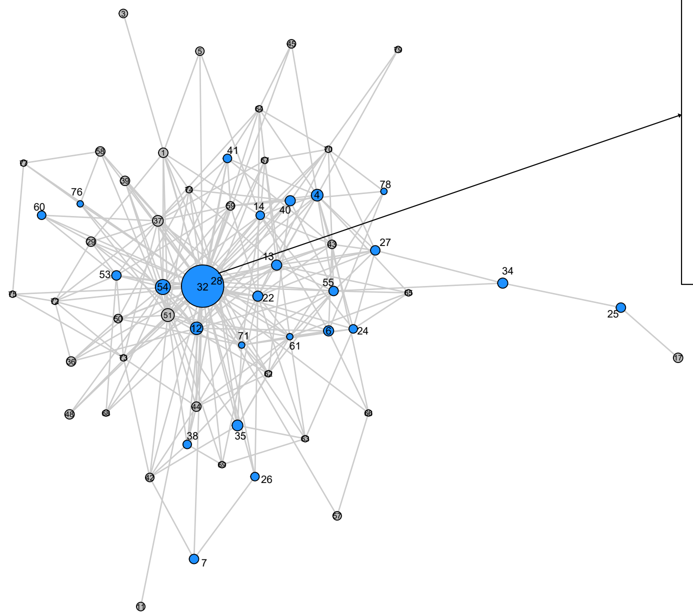

In meta networks (A and D):

Clusters containing secreted proteins or cell adhesion receptors

B Top DE proteins, ligands and receptors (Cluster 32 with 281 proteins)

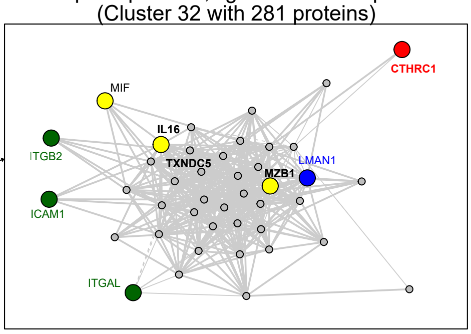

C Top DE proteins, ligands and receptors (Other clusters make up the ECM-enriched domain)

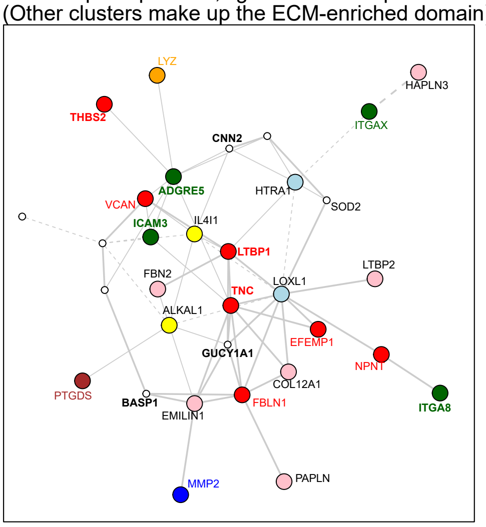

Relative increase in STR MP layer

D **MP Meta network of correlation clusters**

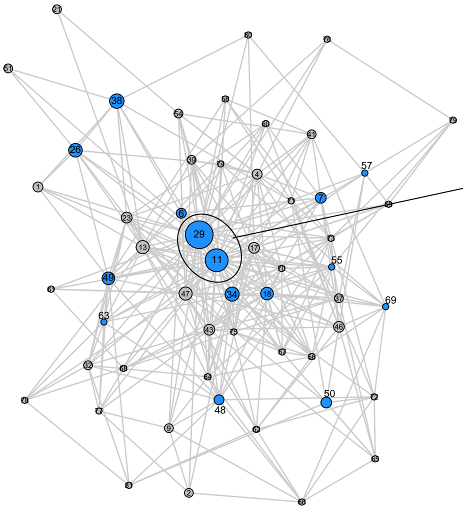

E Top DE proteins, ligands and receptors (Cluster 11 and 29 with 111 proteins)

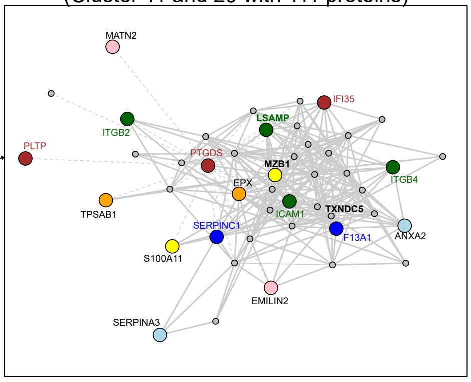

Top DE proteins, ligand, and receptors (Other clusters make up the ECM-Mast cell enriched domain)

F

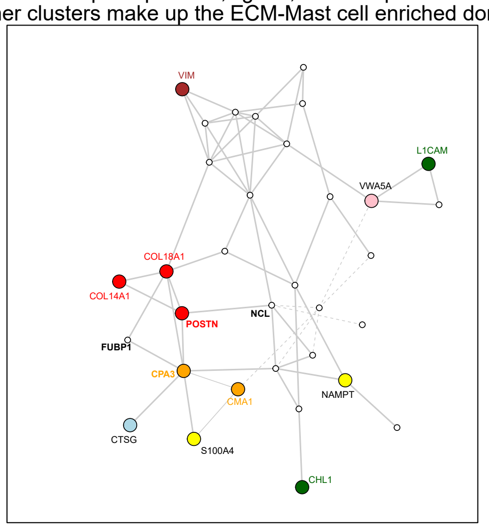

G

Matrisome and matrisome-associated
- ECM (with receptor binding or matricellular)
- Other core ECM proteins
- ECM regulators/ECM-affiliated proteins (with receptor binding)
- ECM regulators/ECM-affiliated proteins

Secreted non-ECM proteins
- Secreted cytokine/cytokine-like molecules
- Granule proteins and similar
- Other secreted

Membrane-bound molecules
- Cell adhesion molecules

Other
- Other Top30 proteins B,E
- Other Top30 proteins C,F

Supplemental Figure 9. Correlation-based network clustering and ECM domain associations. Correlation network analyses show the relationships among positively DE proteins in SM **(A–C)** and MP **(D–F)**. A correlation network was constructed using strong associations (average Spearman's rank correlation > 0.85; see Supplementary Methods 10) as edges. The Markov Cluster Algorithm (MCL) was then applied to identify network clusters. To simplify the structure and capture inter-cluster relationships, meta-network was generated [shown in **(A** and **D)**] by collapsing intra-cluster edges and summarizing all edges between any two clusters into a single representative edge. **(A)** displays the resulting full correlation cluster meta-network. Each node represents an MCL-defined cluster, with edges reflecting aggregated correlation scores between clusters. Node size is related to cluster size, with larger dots indicating larger cluster size. The central and largest cluster (Cluster #32) includes 281 proteins, encompassing CTHRC1 and many top-ranked immune and ER-associated proteins [of which a subset is visible in **(B)**]. The remaining clusters are smaller and include several clusters enriched for secreted proteins and cell adhesion receptors (small blue clusters other than cluster #32). These are collectively referred to as the ECM-enriched domain, as most ECM proteins were found within these clusters [of which a subset is visible in **(C)**]. **(B** and **C)** show correlation networks of secreted ECM proteins, cytokine-like molecules, and cell adhesion molecules along with the intracellular top 30 ranked DE proteins split by their position in **(A)**, with **(B)** showing the intercorrelation between proteins mapped to Cluster 32 while **(C)** shows the intercorrelation between proteins mapped to the ECM-enriched domain. In **(B** and **C)**, each node represents a protein, and edges indicate strong average Spearman correlation coefficients (>0.85). Node colors reflect functional categories as indicated in **(G)** (see text and Supplemental Table 5 for details). Edge metadata (line thickness, thick or thin; line type, solid or dashed) provide information about the source and quality of each edge. Line thickness indicates whether the correlation coefficient was available in both datasets (TMT-MS and TIMS-TOF-MS; thick) or only one dataset (thin). Line type reflects data quality with solid lines representing edges based on sufficient data while dashed lines indicate limited data for that variable combination (see Supplementary Methods 10). In **(C)**, several top-ranked intracellular proteins (white nodes) characterized by strong correlation to ECM proteins are labeled. **(D–F)** show the results for the MP layer and are analogous to **(A–C)**. Note: In the MP layer, assignment of proteins to either of the two largest clusters (#11 and #29; 111 proteins in total) shown in **(E)** was used to separate these secreted/adhesion molecules and other intracellular top ranked DE proteins from those assigned to any of the remaining smaller clusters shown in **(F)**. In **(F)**, note the intercorrelation between top DE mast cell proteins (orange) and ECM proteins (red) (see the main text for details).

Supplemental Figure 10

Concordant increase

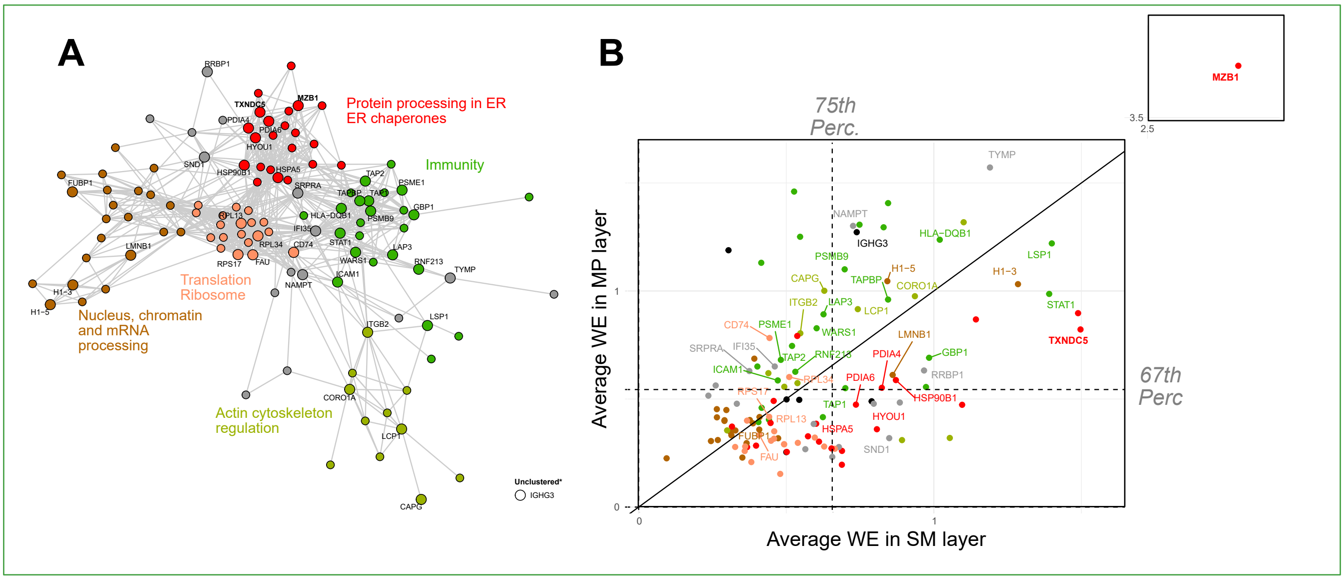

Concordant decrease

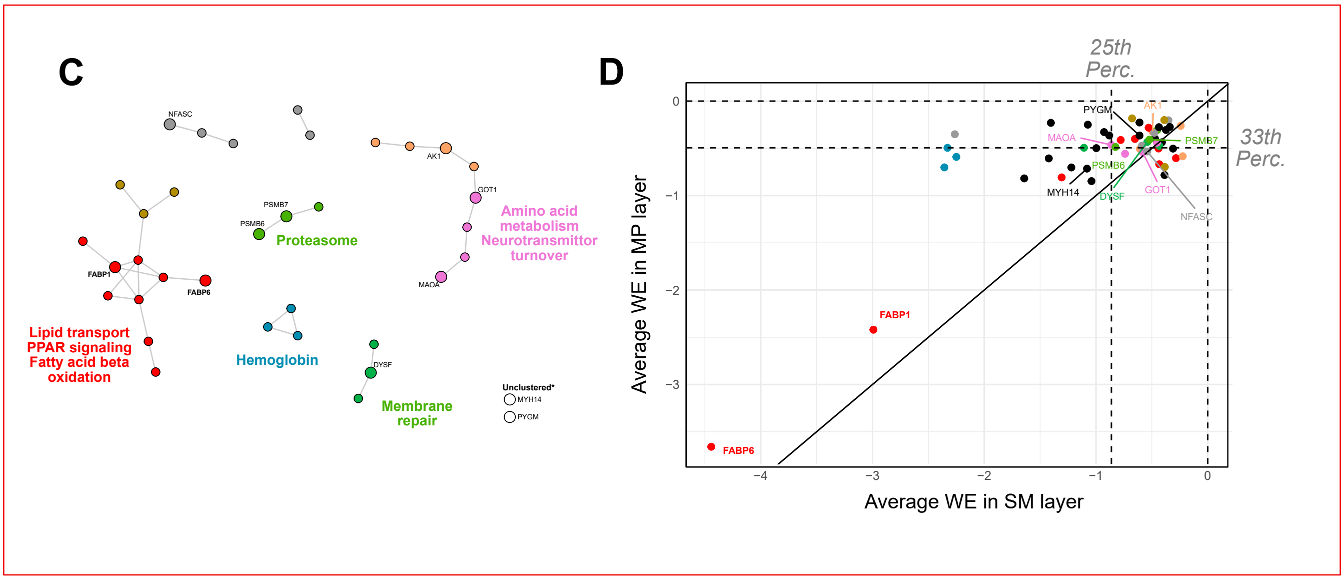

E
SM increased; MP decreased

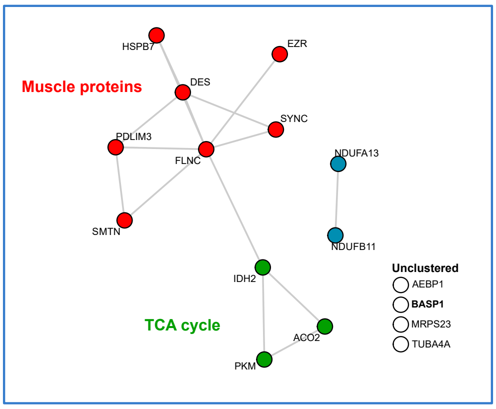

F
SM decreased; MP increased

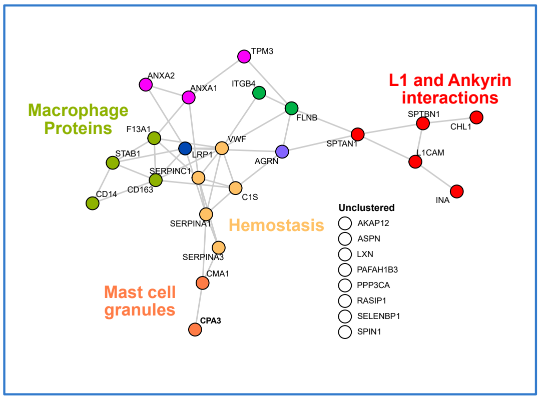

Supplemental Figure 10. Extended interlayer analysis. This figure complements Figure 8 and provides further details about DE proteins displaying concordant increase **(A** and **B**), decrease **(C** and **D)** or opposing expression **(E** and **F)** across layers. **(A** and **C)** show STRING Protein-Protein Interaction (PPI) networks with nodes representing proteins and edges with interaction scores ≥ 0.4. Identified network clusters, or groups thereof, are in different colors and general umbrella terms are annotated (Supplementary Methods 10). **(B** and **D)** Scatterplots visualizing the magnitude of change (Log_2 fold change relative to controls) in SM versus MP as described for Figure 8B. The dashed lines indicate percentile thresholds for the average WE as in the scatterplots in Figures 4–7 but calculated among all DE proteins in each layer and direction (positive or negative). The diagonal line (y = x) indicates equal average WE (equal magnitude of change relative to controls) in both layers. In **(A–D)**, proteins DE in both SvCTRL and SvNSTR comparisons in both layers are labeled. For the 'Unclustered' nodes in the bottom right of Panels **A** and **C**, the asterisk indicates that only those DE in both SvCTRL and SvNSTR comparisons are shown. Proteins specifically mentioned in the text are in bold. **(E** and **F)** PPI network as in **(A** and **C)** but for the proteins showing opposing DE across layers. All proteins are labeled and all unclustered nodes are shown.

Supplemental Figure 11

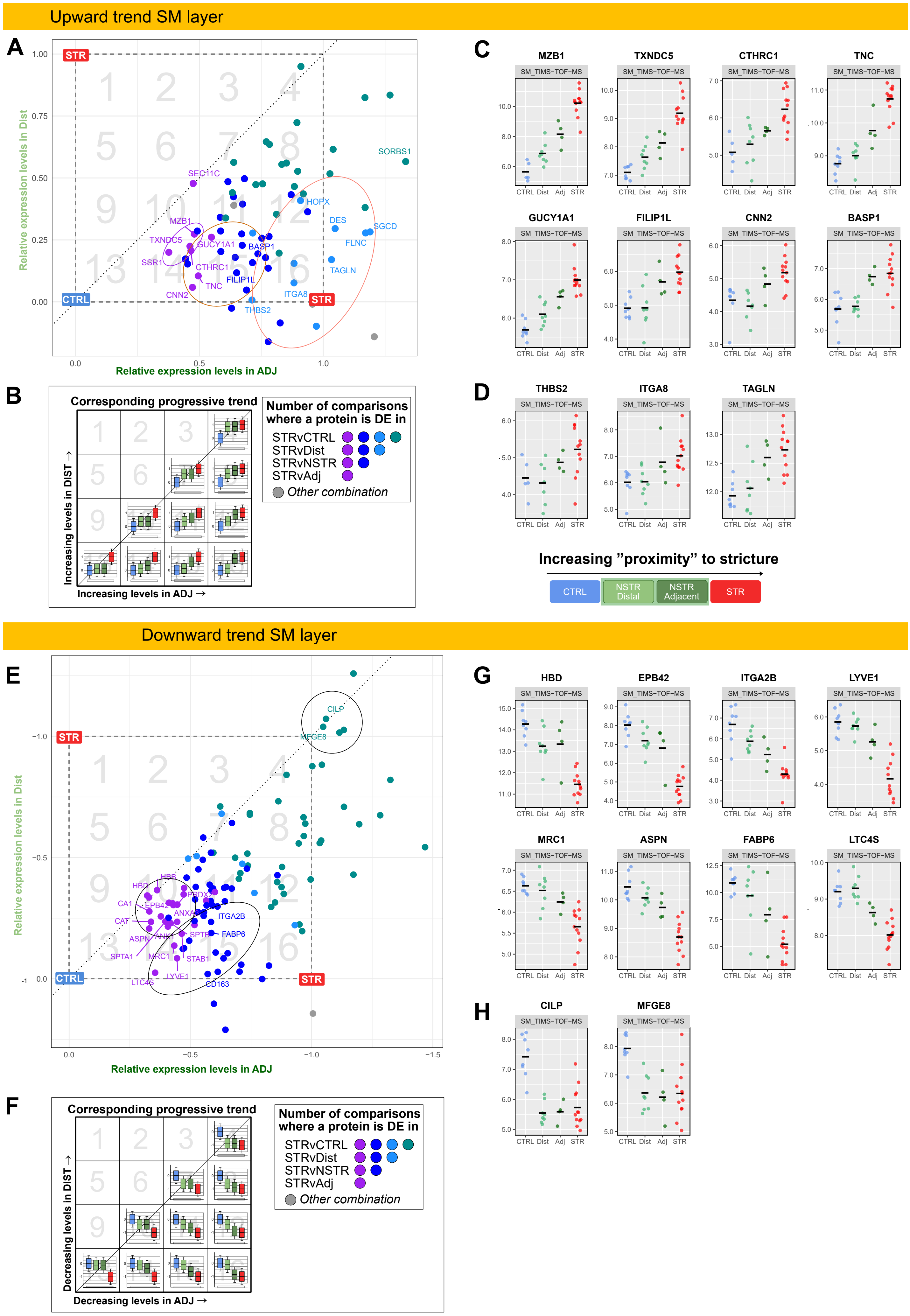

Supplemental Figure 11. Extended analysis of progressive protein expression trends. This figure complements Figures 9 and 10 and displays the number of comparisons where a protein in (Figures 9A and 10A) is DE and shows the TIMS-TOF MS data corresponding to the TMT MS data in (Figure 9, C and D; Figure 10, C and D). **(A)** The same plot of the 70 filtered proteins increased in the SM layer and having an upward expression trend as in Figure 9A (see Supplementary Methods 10). Here, however, dots representing proteins are colored according to the number of comparisons to STR that resulted in DE (four, three, two or one dots, as indicated with the same color vertically in the inset of panels B and F). Thus, different regions within the grid are associated with a distinct number of comparisons. (**B**) As for the legend to Figure 9, each numbered square displays a schematic boxplot pattern of the data for a representative dot (protein) in that square. Since CTRL and STR are fixed at 0 and 1, respectively, and Adj and Dist are positioned based on these anchor points, each dot represents a distinct progressive expression pattern for each protein from CTRL → Dist → Adj → STR as indicated by the color-coding in the inset. Thus, panel **A** can be overlaid on, or cross-referenced with, **B** to approximate a protein's expression pattern based on its position in the CTRL-STR scale. Note that in Panel A and Figure 9A, one protein (IGKC) is outside the displayed x-axis range; see Supporting Data Values for details. **(C** and **D)** correspond to the plots in (Figure 9, C and D), but with the TIMS-TOF MS data as validation to the TMT-MS data. Note that HOPX was not quantified with TIMS-TOF MS. **(E–H)** parallels **(A–D)** except the data in (**E** and **F**) are for the 113 filtered proteins decreased in the SM layer and with a progressive downward trend while (**G** and **H**) display the TIMS-TOF MS data corresponding to the TMT MS plots in (Figure 10, C and D). See the legend to Figures 9 and 10 and Supplementary Methods 10 for details.

Supplemental Tables 1-13

Supplemental Table 1. Clinical information 48

Supplemental Table 2. Additional information to selected markers named in Figure 2, B–D with positive, negative or low correlation to PC1 in the SM layer 49

Supplemental Table 3. Additional information to pathway clusters in Supplemental Figure 3D. 50

Supplemental Table 4. Additional information to markers named in Figure 3, B–D with positive, negative or low correlation to PC1 in the MP layer 51

Supplemental Table 5. Differentially expressed secreted proteins and cell adhesion molecules with a relative increase in the SM layer 52

Supplemental Table 6. Further information about selected top 30 DE proteins increased in STR SM (see Figure 4, B–D) 53

Supplemental Table 7. Proteins correlating strongly to ECM proteins 54

Supplemental Table 8. Further information about selected top 30 DE proteins decreased in the SM layer (see Figure 5, B–D) 55

Supplemental Table 9. Additional information on ECM- and muscle-associated proteins displaying different expression trends with distance to STR (See Figures 9 and 10; Supplemental Figure 11) 56

Supplemental Table 10. Further information about selected top 30 DE proteins increased in the MP layer (see Figure 6, B–D) 57

Supplemental Table 11. Differentially expressed secreted proteins and cell adhesion molecules with a relative increase in the MP layer 58

Supplemental Table 12. Further information about selected top 30 DE proteins decreased in the MP layer (see Figure 7, B–D) 59

Supplemental Table 13. Further information about proteins top ranked in both the SM and MP layers 60

References to Supplemental Tables 61

Supplemental Table 1. Clinical information

ID	Sex	Age	Use nicotine	Montreal classification: Age at diagnosis	Montreal classification: Location	Montreal classification: Behavior	Disease duration (years)	Disease activity[A]	HBI Score	HBI score date[B]	Treatment at surgery
**CD01**	F	48	Y	A3	L1	B2	2	2[C]	3	8	no active treatment
**CD02**	M	28	Y	A1	L1	B2	20	2[C]	4	7	thiopurines (azathioprine)
**CD03**	F	66	Ex	A3	L1	B2	9	3[D]	12	13	anti-TNF (infliximab)
**CD05**	M	51	N	A3	L1	B2	23	3[D]	9	12	anti-TNF (infliximab), thiopurines (mercaptopurine)
**CD06**	M	47	N	A1	L1	B2	34	2[C]	8	9	anti-integrin α4β7 (vedolizumab)
**CD17**	M	43	N	A2	L3	B3	27	3[D]	0	21	anti-IL12/23 (ustekinumab)
**CD07**	F	23	N	A1	L1	B2	12	2[C]	4	9	anti-IL12/23 (ustekinumab)
**CD08**	F	57	Ex	A2	L1	B2	42	2[C]	15	0	corticosteroids
**CD10**	F	48	Ex	A2	L1	B2	38	2[C]	10	84	thiopurines (mercaptopurine), allopurinol
**CD11**	F	50	NA	A2	L1	B2	20	NA	NA	NA	anti-TNF (adalimumab)
**CD12**	M	28	N	A2	L1	B3	0	2[C]	7	14	corticosteroids (withdrawn 1 week before surgery), thiopurines (azathioprine)
**CD16**	F	34	Ex	A2	L1	B1[E] (B2)	11	2[C]	5	22	no active treatment
**CTRL03**	F	80	NA	NA	NA	NA	NA	NA	NA	NA	NA
**CTRL04**	F	80	NA	NA	NA	NA	NA	NA	NA	NA	NA
**CTRL05**	F	51	NA	NA	NA	NA	NA	NA	NA	NA	NA
**CTRL06**	M	69	NA	NA	NA	NA	NA	NA	NA	NA	NA
**CTRL08**	M	78	NA	NA	NA	NA	NA	NA	NA	NA	NA
**CTRL11**	M	67	NA	NA	NA	NA	NA	NA	NA	NA	NA
**CTRL13**	F	77	NA	NA	NA	NA	NA	NA	NA	NA	NA
**CTRL15**	M	67	NA	NA	NA	NA	NA	NA	NA	NA	NA

[A]Since debut. [B]Days before surgery. [C]Short periods of disease remission with frequent periods of disease activity. [D]Long periods of disease remission with few periods of disease activity. [E]At the time of surgery. Abbreviations: HBI, Harvey-Bradshaw Index (HBI); Y, Yes; N, No; Ex, Ex-user; NA, Not available/Not applicable.

Supplemental Table 2. Additional information to selected markers named in Figure 2, B–D with positive, negative or low correlation to PC1 in the SM layer

**Positive correlation to PC1**	
**Functional category**	**Cell marker**
Immune cell markers related to pro-inflammatory processes (Green ellipse, right):	
Pan-leukocyte marker	CD45/PTPRC
B-cell/Plasma cell chaperone	MZB1
Antigen-presentation cell MHC II invariant chain	CD74
Adhesion molecules for leukocyte	ITGAL, ITGB2, ICAM1
Macrophage proteins related to cytoskeletal regulation	ARHGAP30, CORO1A, DOCK2
Antibody (IgG3) heavy chain constant region	IGHG3
**Core matrisome (orange ellipse, right):**	**Proteins**
Proteoglycans	VCAN, FMOD, HAPLN3
ECM glycoproteins	TNC, MXRA5, LTBP1, CTHRC1
HPA fibroblast proteins	LSAMP, MYH11, NT5E, TIMP
**Negative correlation to PC1**	
**Functional category**	**Cell marker**
Immune cell markers related to tissue residency and scavenging functions (Green ellipse, left)	MRC1, CD163, SIGLEC1
Adhesion molecules involved in glia-neuron interactions (pink ellipse, left)	L1CAM, CADM1, CADM2, CDH19
Endothelial homeostasis (khaki ellipse, left)	NOS3, ESAM
Red blood cell-specific proteins	HBA1, HBB, HBD, EPB42
**Core matrisome (orange ellipse, left)**	**Proteins**
Fibrillar collagens	COL1A2, COL3A1
Proteoglycans	ASPN, BGN, DCN
ECM glycoproteins	VWF, MMRN2, fibrinogens
HPA fibroblast protein	CD248
**Low correlation to PC1** (Other proteins named in **Figure 2, B and C** and located nearer the center in **Figure 2B**)	
**Function**	**Cell marker**
Cytotoxic T-cell surface protein	CD8A
Macrophage LPS co-receptor	CD14
Mast cell granule proteins	CMA1, TPSAB1, CPA3
Macrophage scavenger receptor	MSR1
Neuronal filaments	NEFM, NEFH, NEFL
Neuron-specific enolase	ENO2
Antibody-related proteins	IGHG2, IGHG4, IGHA1, IGHM, IGKC
Endothelial marker	PECAM1/CD31
Neutrophil (calprotectin)	S100A8 and S100A9
Eosinophils	EPX

Supplemental Table 3. Additional information to pathway clusters in Supplemental Figure 3D. Examples of core enriched proteins mapped to enriched pathways in selected clusters.

**Positive enrichment**		
**Pathway network cluster**	**Functions of core enriched proteins**	**Proteins**
Immune-related pathways	Immune cell membrane proteins<br>Immunoproteasome subunits<br>Antigen processing<br>Pro-inflammatory cytokines<br>Immune receptor signal transduction<br>Transcription factors<br>Interferon-induced proteins	(see Supplemental Table 2)<br>PSMB8, PSMB9<br>TAP1, TAP2<br>MIF, IL-16<br>GRB2, PLCG2<br>STAT1*, STAT2<br>GBP1, GBP2, CORO1A*
Unfolded protein response (UPR); response to stress and stimuli	Functions related to protein folding, inhibition or degradation of misfolded proteins and quality control in ER	HSP90B1*, HYOU1, PREB, PDIA6, DNAJB11, HSPA5, HERPUD1
MYC Targets	Stress adaptation, redox signaling<br>Pyrimidine biosynthesis<br>RNA-binding proteins<br><br>Various ribosomal protein<br>tRNA synthetase (proline-rich protein synthesis e.g. collagen)	PRDX4<br>CAD<br>PABPC1*, PABP1, HNRNPA2B1<br>RPS6<br>EPRS1
Metabolism of protein and RNA	Ribosomal subunits<br>Elongation factors<br>tRNA synthetase<br>ER receptor<br>OST-complex/N-linked glycosylation<br>Trafficking between ER and Golgi	RPS2*, RPS3*, RPS18*<br>EEF1G*, EEF2*,<br>VARS1*<br>SSR1*<br>RPN1*, RPN2*<br>COPA*, COPB1*, LMAN2*, TMED9*
**Negative enrichment**		
**Pathway network cluster**	**Functions of core enriched proteins**	**Proteins**
Neuronal processes	Ankyrins and spectrins<br>Members of the L1CAM family<br>Neurotransmitter degradation<br>Synaptic proteins	ANK2, ANK3, SPTBN2<br>L1CAM, NFASC, CHL1<br>ACHE, MAOA<br>STXBP1, HOMER3
Heme metabolism and hemostasis/complement	Red blood cell proteins<br><br>Platelets<br><br>Plasma components	membrane proteins, hemoglobin complex<br>granule proteins, integrins<br>fibrinogen, coagulation factors, complement proteins, lipoproteins
ECM organization	ECM structural/Core matrisome<br>Platelet granules<br>Platelet integrins<br>Basal membrane<br>Cell junctions<br>Matrix degradation	See Supplemental Table 2<br>A2M, VWF, fibrinogens<br>ITGA2B, ITGB3<br>COL4A6, LAMA2, AGRN<br>JAMA3<br>ADAM10, ADAM15, MMP10, PRSS2

**Proteins among the Top 30 correlations are indicated with an asterisk* (italic, printed with leading asterisk)

Supplemental Table 4. Additional information to markers named in Figure 3, B–D with positive, negative or low correlation to PC1 in the MP layer

**Positive correlation to PC1:**	
**Functional category**	**Cell marker**
Immune cell markers:	
Antigen-presentation cell MHC II invariant chain	CD74
Antibody related proteins	IGHA1, IGHG2, IGHG4 and JCHAIN
Mast cell secretory granule proteins	CTSG, CPA3, TPSAB1
Macrophage proteins (actin dynamics)	CORO1A, NCKAP1L
Macrophage scavenger receptor	CD163
Endothelial cell:	
Angiogenesis-related	RASIP1, SWAP70, WARS1
HPA fibroblast proteins	LSAMP, S100A4, NT5E/CD73
**Core matrisome**	Proteins
Non-fibrillar collagens	COL14A1, COL18A1
ECM glycoproteins	POSTN, PAPLN, VWA5A, EMILIN2
**Negative correlation to PC1:**	
**Functional category**	**Cell marker**
SMC markers:	
Canonical SMC proteins	DES, SYNM, SMTN, MYH11
Other	DYSF
**Low correlation to PC1** (Other protein named in Figure 3, B and C and located nearer the center in (Figure 3B)	
**Function**	**Cell marker**
Adhesion molecules involved in glia-neuron interactions	L1CAM, CADM1, CDH19, CHL1
Eosinophils	EPX
Other SMC markers	CNN1, ACTG2, TAGLN
Other	MXRA8

Supplemental Table 5. Differentially expressed secreted proteins and cell adhesion molecules with a relative increase in the SM layer

	**1. Matrisome and matrix-associated**	**Proteins**
	1.1 Core ECM Proteins with receptor interaction (L)	**CTHRC1**, **EFEMP1**, **LTBP1**, **TNC**, **VCAN**, FBLN1, NPNT, THBS2, *(EFEMP2, NID2)*
	1.3 ECM core proteins other (No RL)	**FBLN5**, **FBN2**, COL12A1, COL15A1, EMILIN1, LTBP2, MXRA5, PAPLN, PXDN, FMOD, HAPLN3, PRG3, *(AEBP1, MFAP1)*
	1.4 ECM Regulators with receptor interaction (L)	MMP2, *(F9)*
	1.5 ECM Regulators others (No_RL)	HTRA1, LOXL1
	1.7 ECM-Affiliated Proteins with receptor interaction (L)	LMAN1
	1.8 ECM-Affiliated Proteins other (No_RL)	*(ANXA6, PLXDC2)*
	**2. Secreted Signaling or Non-ECM Proteins**	
	2.1 Cytokine-like (L)	NAMPT, ALKAL1, MIF
	2.2 Matrix-associated secreted factors (L)	**IL16**, GREM1
	2.3 Other Cytokine-like secreted proteins (No_RL)	**MZB1**, IL4I1
	**3. Secreted Granule Proteins or other**	
	3. Secreted Granule Proteins (Immune Effector / ECM-Impacting)	EPX
	3.1 Secreted antimicrobila proteins (L)	LYZ
	**4. Other secreted or ligands**	
	4.2 Other proteins with keyword secreted (No_RL)	**PTGDS**, *(IFI35)*
	**5. Cell adhesion proteins (non-ECM)**	
	5.1 Adhesion ligands (CD antigens) (L)	**ICAM3**, ICAM1
	5.2 Adhesion receptors (CD antigens/integrins) (R)	**ADGRE5**, ITGB2, ITGA8, ITGAL, ITGAX

The mapping was based on Matrisome database, CellTalkDb and Uniprot keywords (Supplementary Methods 11). The color bars on the left correspond to the colors used to denote proteins in Supplemental Figure 9. The information in parenthesis after each category name indicates if proteins in this category are Ligands (L), Receptors (R), or neither (No RL) according to CellTalkDb. Proteins colored and in bold represent the top 30 DE proteins. The grey box behind the protein names in yellow is simply to increase visibility. Proteins listed in italics and parentheses were not included in the correlation analysis (see Supplementary Methods 10).

Supplemental Table 6. Further information about selected top 30 DE proteins increased in STR SM (see Figure 4, B–D)

**ER-associated cluster**	
**Name and function**	**Protein**
Chaperones and folding:	
B-cell chaperone aiding in folding of antibodies (1). May act as a cytokine/adipokine (2)	**MZB1** (T_{30})
Protein disulfide isomerase enriched in fibroblasts and implicated in fibrosis across several organs (3)	**TXNDC5** (T_{30})
Chaperone involved in folding integrins, Toll-like receptors, and cytokines.	HSP90B1 (C)
Stress adaption/Redox:	
ER protein induced by the unfolded protein response and oxidative stress.<br>Role in oxidative stress protection and redox signaling.	HERPUD1<br>PRDX4
Mitochondrial protein involved in reactive oxygen species (ROS) detoxification.	SOD2
Secretory pathways:	
Peptide translocation to the ER, glycosylation, and protein export.	SEC11C
**Immune cluster**	
**Name and function**	**Protein**
Transcription factor (Immune and growth factor signaling)<br>Phagocyte IgG receptor<br>F-actin binding protein	STAT1 (T_{30})<br>FCGR1A<br>LSP-1 (T_{30})
Membrane-bound adhesion molecules and cytokines:	
Leukocyte adhesion molecule.<br>Adhesion G protein-coupled receptor implicated in leukocyte migration and signaling processes. Also known as CD97.<br>Matrisome-associated cytokine with chemoattractant properties that also stimulate responsiveness to other cytokines (4-6). ↑ expression in IBD colon mucosa (7) and CD colon (8, 9). Reported expression in T cells and mast cells in active CD (8)	**ICAM3**<br>**ADGRE5**<br><br>**IL16**
**ECM cluster**	
**Name and function**	**Protein**
Matricellular ECM glycoprotein protein linked to tissue regeneration, inflammation, and fibrosis progression (10-14). [Tenascin-C]. Abundantly produced IBD SM myofibroblasts in vitro (15).	**TNC**
ECM glycoprotein implicated in TGF-β and WNT signaling pathways and is proposed to play both regulatory and promotive roles in a context dependent manner during fibrosis progression (16). Mark pathogenic fibroblasts (10, 17, 18)	**CTHRC1**
ECM glycoprotein involved in sequestration and maintaining extracellular TGF-β in a latent state for controlled activation and release (10).↑Full-thickness CD stricture ECM (19)	**LTBP1**
Proteoglycan with reported immune- (20) and fibrogenesis (21, 22) modulatory. Correlate with increased TNC in idiopathic pulmonary fibrosis (23).	VCAN (C)
**ECM associated/correlated (see Supplemental Figure 9C)**	
Marker of specific fibroblast subsets in various organs (24)	**GUCY1A1**
Filament-associated protein implicated in regulation and modulation of smooth muscle contraction. Associated with metabolic reprogramming and fibrosis in experimental kidney fibrosis (25).	**CNN2** (C)
Originally associated with functions in neuronal tissue and development but appear to have functions outside nervous system as well. May function as a lipid-dependent transcriptional corepressor (26). Expressed by monocyte subset in vascular disease (27). Prognostic factor in certain cancers.	**BASP1** (O_{30})

Proteins discussed in the text are indicated in ***bold****. Information within parenthesis provides additional information from the interlayer analysis (Figure 8 and Supplemental Figure 10): (T_{30}) are DE proteins that are also among the top 30 in the MP layer; (C) display concordant DE but is not among the top 30 DE proteins in the MP layer; (O_{30}) DE proteins among the top 30 in the opposite direction in the MP layer.*

Supplemental Table 7. Proteins correlating strongly to ECM proteins

ECM protein	Secreted and adhesion proteins	Other proteins (intracellular or other membrane receptors)
**CTHRC1** [32]	**TNC** [55]	DGKA [12], TOP1 [32], BCL3 [32], PTDSS1 [40], STK4 [32], GALNT2 [32], NCKAP1L [32], DENND2D [54], APOBEC3G [32], TCOF1 [32], RRP12 [32], HERPUD1 [32], SLC38A10 [32], TAPBP [32], ARHGAP30 [32], TRIR [54], FNDC3A [32], SMCHD1 [32], HM13 [32], RPL13A [32]
EFEMP1 [55]	**TNC** [55], LOXL1 [27]	**FILIP1L** [65]
EFEMP2 [12]		RPL11 [44], DGKA [12]
FBLN1 [13]	**TNC** [55], LOXL1 [27], COL12A1 [55], EMILIN1 [12], PAPLN [12]	DGKA [12], RASSF2 [43], **BASP1** [13], **GUCY1A1** [13], GFER [12], FTH1 [13], **FILIP1L** [65]
**LTBP1** [4]	IL4I1 [24], HTRA1 [22], LOXL1 [27], VCAN [24], **TNC** [55], FBN2 [4]	RASSF2 [43], PDLIM4 [79], **GUCY1A1** [13]
NPNT [34]	LOXL1 [27], **ITGA8** [25]	TES [34], BAG2 [34], CORO1C [34]
**THBS2** [78]	**ADGRE5** [61]	BIN2 [6], **HOPX** [70], ZC3H18 [59]
**TNC** [55]	LOXL1 [27], COL12A1 [55], FBLN1 [13], EFEMP1 [55], **ICAM3** [71], EMILIN1 [12], **CTHRC1** [32], **LTBP1** [4], **ADGRE5** [61]	DGKA [12], PTDSS1 [40], STAT2 [32], PRKCB [28], RASSF2 [43], LRCH4 [28], TIMM10 [32], **GUCY1A1** [13], **FILIP1L** [65], BCL3 [32], CISD2 [62], BAG2 [34]
VCAN [24]	IL4I1 [24], **ICAM3** [71], **ADGRE5** [61], **LTBP1** [4]	FCGR1A [14], **FILIP1L** [65]

The ECM proteins listed in the left column are those identified as having receptor interactions (colored in red in Supplemental Table 5 and Supplemental Figure 9). The middle and right columns list proteins that exhibit a strong positive correlation (weighted average of Spearman's rho > 0.85; see Supplementary Methods 10) with each ECM protein, grouped by category. Within each category, proteins are ordered by correlation strength in descending order. Correlation clusters, corresponding to Supplemental Figure 9A, are shown in brackets. Proteins discussed in the text are indicated in ***bold****. Blue represents proteins outside the top 30 DE proteins mentioned in the text and listed in Supplemental Table 9.*

Supplemental Table 8. Further information about selected top 30 DE proteins decreased in the SM layer (see Figure 5, B–D)

Vessels	
**Function/name**	**Protein**
Erythrocytes<br>Hemoglobin complex and gas exchange<br>Hemoglobin Subunit Beta and Delta<br>Carbonic Anhydrase 1<br><br>Membrane and membrane stability<br>Erythrocyte Membrane Protein Band 4.2<br>Erythrocytic spectrin alpha 1 and beta<br>Ankyrin 1<br><br>***Other blood-related proteins***<br>Platelet integrin<br><br>***Endothelial lining proteins***<br>Endothelial hemostasis-related surface proteins<br>Lymphatic endothelium receptor	 <br>HBB (C), HBD (C)<br>CA1<br><br><br>EPB42<br>SPTA1, SPTB (C)<br>ANK1<br><br><br>**ITGA2B**<br><br><br>PROCR, VWF (O)<br>**LYVE1**
**Lipid metabolism**	
**Function/name**	**Protein**
Lipid transport and PPAR signaling (28)<br>Fatty Acid binding protein 6/Gastrotropin. ↓Fibrostenotic intestine (29)<br>Fatty Acid binding protein 1<br><br>***Arachidonic acid metabolism***<br>Leukotriene C4 Synthase<br>Prostaglandin-endoperoxide synthase 1 (COX1)	 **FABP6** (T_{30})<br>**FABP1** (T_{30})<br><br><br>LTC4S<br>PTGS1
**Other proteins**	
**Function/name**	**Protein**
Antioxidant defense and detoxification	PRDX2, CAT

Proteins discussed in the text are indicated in ***bold****. Information within parenthesis provides additional information from the interlayer analysis (Figure 8 and Supplemental Figure 10): (T_{30}) are DE proteins that are also among the top 30 in MP layer; (C) display concordant DE but is not among the top 30 DE proteins in the MP layer; (O) Display DE in the opposite direction in the MP layer.*

Supplemental Table 9. Additional information on ECM- and muscle-associated proteins displaying different expression trends with distance to STR (See Figures 9 and 10; Supplemental Figure 11)

**ECM- and Muscle-associated proteins (pink ellipse)**	
**Name and function**	**Protein**
Integrin α8. A non-leukocyte integrin that pairs with β1 (ITGB1) to form Integrin α8 β1 and binds TNC and other ECM (30)	**ITGA8**
Associated with transcriptional regulation of cardiomyocyte maturation programs (31)	**HOPX**
Matricellular protein. Localizes to MM, blood vessel, MP and subserosal fibrotic regions in fibrostenotic intestine (32)	**THBS2**
Several actin and SMC associated proteins	DES, TAGLN, FLNC, SGCD
**Other proteins (brown ellipse)**	
**Name and function**	**Protein**
Anti-angiogenic factor (Uniprot)	**FILIP1L**

*Proteins discussed in the text are indicated in **bold***

Supplemental Table 10. Further information about selected top 30 DE proteins increased in the MP layer (see Figure 6, B–D)

B-cells and antibodies

Function	Protein
Chaperone for antibodies (1). May act as a cytokine/ adipokine (2).	**MZB1** (T_{30})
Antibody related proteins:<br>Junctional chain<br>Various antibody proteins	 JCHAIN<br>IGHA1, IGHG3 (C), IGLC2

Granulocyte granule proteins

Function	Protein
Mast cells granules:<br>Carboxy peptidase<br>Chymase, Mast cells (STRvCTRL only*)<br>Tryptase, Mast cells<br>***Eosinophil granules:***<br>Eosinophil peroxidase (STRvDist only*)<br>***Other granules (neutrophils/mast cells):***<br>Cathepsin G<br>**The fold changes for these granules were high in several comparisons but significant only in one*<br>*↑Mast cells (33) and Activated eosinophils (34) in MP or deeper layers of fibrostenotic intestine previously reported*	 **CPA3** (O)<br>CMA1 (O)<br>TPSAB1<br><br>EPX (C)<br><br>CTSG

Other immune related proteins

Function	Protein
Immune cluster 1:<br>Antigen presentation<br>Immunoproteasome subunits<br>Transcription factor	 MHCI/II (C), TAPBP (C)<br>PSMB9 (C), PSMB10 (C)<br>STAT1 (T_{30})
Immune cluster 2:<br>Actin-binding protein in motile cells<br>F-actin binding protein	 CORO1A (T_{30})<br>LSP1 (T_{30})

ECM proteins

Function	Protein
Matricellular ECM glycoprotein linked to tissue regeneration and fibrosic processes (10). POSTN-cell interactions associated with activation of multiple signaling pathways (35). Implicated in cardiac hypertrophy (36) and vascular SMC migration (37).<br>***Collagens:***<br>Precursor to endostatin which inhibits angiogenesis<br>FACIT (fibril-associated collagens with interrupted triple helices) (STRvNSTR, STRvsDist)	**POSTN**<br><br><br>COL18A1<br>COL14A1

Other (ER and cell adhesion)

Function	Protein
See above under B-cells<br>Protein disulfide isomerase enriched in fibroblasts and implicated in fibrosis across several organs (3)	**MZB1** (T_{30})<br>**TXNDC5** (T_{30})
Cell adhesion molecule involved in axon guidance. Also expressed by fibroblasts	**LSAMP**

*Proteins discussed in the text are indicated in **bold.** Information within parenthesis provides additional information from the interlayer analysis (Figure 8 and Supplemental Figure 10): (T_{30}) are DE proteins that are also among the top 30 in the SM layer; (C) display concordant DE but is not among the top 30 DE proteins in the SM layer; (O) display DE in opposite direction in the SM layer.*

Supplemental Table 11. Differentially expressed secreted proteins and cell adhesion molecules with a relative increase in the MP layer

	**1. Matrisome and matrix-associated**	**Proteins**
	1.1 Core ECM Proteins with receptor interaction (L)	**COL14A1**, **COL18A1**, EFEMP1, VCAN, *(AGRN, FBLN1, LAMA4, VWF)*
	1.2 ECM core proteins other matricellular (MC)	**POSTN**
	1.3 ECM core proteins other (No RL)	EMILIN2, MATN2, VWA5A, ASPN
	1.4 ECM Regulators with receptor interaction (L)	F13A1, SERPINC1, *(SERPINA1)*
	1.5 ECM Regulators others (No RL)	**CTSG**, SERPINA3, *(SERPINB1, SERPINF1)*
	1.6 ECM-Affiliated Proteins receptor (R)	(GPC4)
	1.7 ECM-Affiliated Proteins other (No RL)	ANXA2
	**2. Secreted Signaling or Non-ECM Proteins**	
	2.1 Cytokine-like (L)	**NAMPT**
	2.2 Matrix-associated secreted factors (Mixed)	S100A4 (L), S100A11 (No RL), *(ANXA1 (L))*
	2.3 Other Cytokine-like secreted proteins (No RL)	**MZB1**
	**3. Secreted Granule Proteins**	
	3. Secreted Granule Proteins (No RL)	**CMA1**, **TPSAB1**, **CPA3**, **EPX**
	**4. Other secreted or ligands**	
	4.1 Other secreted proteins (ligands) (L)	**VIM**, PLTP, *(LGALS3BP)*
	4.2 Other proteins with keyword secreted (No RL)	**PTGDS**, **C7**, IFI35, *(QSOX1)*
	**5. Cell adhesion proteins (non-ECM)**	
	5.1 Adhesion ligands (CD antigens) (L)	ICAM1, L1CAM,
	5.2 Adhesion receptors (CD antigens/integrins) (R)	**ITGB4**, ITGB2, CD47, *(ALCAM)*
	5.3 Other Cell adhesion molecules (No_RL)	**CHL1**, **LSAMP**

The mapping was based on Matrisome database, CellTalkDb and Uniprot keywords (Supplementary methods 11). The color bars on the left correspond to the colors used to denote proteins in Supplemental Figure 9. The information in parenthesis after each category name indicate if proteins in this category are Ligands (L), Receptor (R), or none of these (No RL) according to CellTalkDb. For the category annotated as mixed, the information is given after each protein. Proteins colored and in bold represent the top 30 DE proteins. The grey box behind the protein names in yellow is simply to increase visibility. Proteins listed in italics and parentheses were not included in the correlation analysis (see Supplementary methods 10).

Supplemental Table 12. Further information about selected top 30 DE proteins decreased in the MP layer (see Figure 7, B–D)

**Metabolism**	
**Function/name**	**Protein**
**Lipid transport and PPAR signaling** (28):<br>Fatty Acid binding protein 6/Gastrotropin, ↓Fibrostenotic intestine (29)<br>Fatty Acid binding protein 1	 **FABP6** (T_{30})<br>**FABP1** (T_{30})
Essential for maintaining mitochondrial cristae morphology and respiratory chain function<br>Catalyzes the first step in ketone metabolism<br>Involved in the maintenance of the redox state of the mitochondria among other functions	OPA1<br><br>OXCT1<br>IDH2 (O), NNT
**Muscle proteins**	
**Function/name**	**Protein**
Proteins with key regulatory roles in muscle cells:	
Non-receptor serine/threonine protein kinase serving key roles in skeletal, cardiac and smooth muscle cells (Uniprot). Reported to be essential for contractility of smooth muscle cells in the gastrointestinal tract (38).	**DMPK**
Essential for sarcomere integrity and metabolic regulation in cardiomyocytes. Loss-of-function linked to hypertrophy, reduced contractility, decreased mitochondrial content, and metabolic reprogramming in model systems of heart failure (39, 40).	**ACTN2**
Essential for cardiomyocyte differentiation (Uniprot). Involved in energy regulation in response to increased demands (41).	SMYD1
Cytoplasmic heat shock proteins in muscle cells	HSPB2, CRYAB
**Other**	
**Function/name**	**Protein**
Contactin-1. Neural adhesion molecule linked to neuromuscular junctions.	CNTN1

*Proteins discussed in the text are indicated in **bold**. Information within parenthesis provides additional information from the interlayer analysis (Figure 8 and Supplemental Figure 10): (T_{30}) are DE proteins that are also among the top 30 in the SM layer; (O) display DE in the opposite direction in the SM layer.*

Supplemental Table 13. Further information about proteins top ranked in both the SM and MP layers

Top ranked in both layers (concordant increase)	
**Function**	**Protein**
**ER**	
B-cell chaperone (1). May also be secreted (cytokine/adipokine) (2)	**MZB1**
Protein disulfide isomerase enriched in fibroblasts and implicated in fibrosis across several organs (3)	**TXNDC5**
Other ER proteins. Protein folding, ER stress/UPR, glycosylation	PDIA6, RPN1
**Angiogenesis**	
Non-canonical functions pro-angiogenic effects and the inhibition of glial cell proliferation	TYMP
**Immune functions**	
Actin-binding proteins	LSP1, CORO1A
Transcription factor	STAT1
Muscle contraction/relaxation	
Enzyme generating prostaglandin PGD2, which regulates muscle contraction and relaxation. Implicated in chemotaxis (42)	PTGDS
Others	
Histone	HI-3
Ribosome-binding ER protein, secretory cells.	RRBP1
Multifunctional protein. Oncoprotein with reported involvement in HIF1a and WNT signaling and EMT (43)	SND1
Component in nuclear lamina; possible link to oxidative stress, MAPK, and senescence (44)	LMNB1
**Top ranked both layers (concordant decrease)**	
**Function**	**Protein**
Lipid transporters	
Fatty acid binding proteins. Also linked to PPAR signaling (28)<br>↓ FABP6 in fibrostenotic intestine previously reported (29)	**FABP6, FABP1**
**Top ranked both layers (increased SM, decreased MP)**	
**Function**	**Protein**
Originally associated with functions in neuronal tissue and development but appear to have functions outside nervous system as well. May function as a lipid-dependent transcriptional corepressor (26). Expressed by monocyte subset in vascular disease (27). Prognostic factor in certain cancers.<br>(In the MP layer, the decrease was observed in SvNSTR and SvDist comparisons)	**BASP1**

*Proteins discussed in the text are indicated in **bold.***

References to Supplemental Tables

1. Bhanuse SG, et al. Unveiling the multifaceted role of MZB1 in health and disease toward potential therapeutics. A comprehensive review. *Int J Biol Macromol*. 2025;321(Pt 4):146399.
2. Zhang H, et al. Novel hormone-regulated genes in visceral adipose tissue: cloning and identification of proinflammatory cytokine-like mouse and human MEDA-7: implications for obesity, insulin resistance and the metabolic syndrome. *Diabetologia*. 2011;54(9):2368-80.
3. Hung C-T, et al. The novel role of ER protein TXNDC5 in the pathogenesis of organ fibrosis: mechanistic insights and therapeutic implications. *J Biomed Sci*. 2022;29(1).
4. Wen Z, et al. Interleukin-16 enhances anti-tumor immune responses by establishing a Th1 cell-macrophage crosstalk through reprogramming glutamine metabolism in mice. *Nat Commun*. 2025;16(1).
5. Mathy NL, et al. Interleukin-16 stimulates the expression and production of pro-inflammatory cytokines by human monocytes. *Immunology*. 2000;100(1):63-9.
6. Niewold TB, et al. Role of interleukin-16 in human diseases: a novel potential therapeutic target. *Front Immunol*. 2025;16:1524026.
7. Seegert D, et al. Increased expression of IL-16 in inflammatory bowel disease. *Gut*. 2001;48(3):326-32.
8. Middel P, et al. Interleukin 16 expression and phenotype of interleukin 16 producing cells in Crohn's disease. *Gut*. 2001;49(6):795-803.
9. Keates AC, et al. Interleukin 16 is up-regulated in Crohn's disease and participates in TNBS colitis in mice. *Gastroenterology*. 2000;119(4):972-82.
10. Rieder F, et al. Fibrosis: cross-organ biology and pathways to development of innovative drugs. *Nat Rev Drug Discov*. 2025(7):543-69.
11. Imanaka-Yoshida K, et al. Tenascin-C in development and disease of blood vessels. *Anat Rec (Hoboken)*. 2014;297(9):1747-57.
12. Bhattacharyya S, et al. Tenascin-C in fibrosis in multiple organs: Translational implications. *Semin Cell Dev Biol*. 2022;128:130-6.
13. Bhattacharyya S, et al. Tenascin-C drives persistence of organ fibrosis. *Nat Commun*. 2016;7:11703.
14. Halper J. Basic Components of Connective Tissues and Extracellular Matrix: Fibronectin, Fibrinogen, Laminin, Elastin, Fibrillins, Fibulins, Matrilins, Tenascins and Thrombospondins. Springer International Publishing; 2021. p. 105-26.
15. Lin SN, et al. Human intestinal myofibroblasts deposited collagen VI enhances adhesiveness for T cells - A novel mechanism for maintenance of intestinal inflammation. *Matrix Biol*. 2022;113:1-21.
16. Cao M, et al. The role and molecular mechanism of CTHRC1 in fibrosis. *Life Sci*. 2024;350:122745.
17. Bauer-Rowe KE, et al. Creeping fat-derived mechanosensitive fibroblasts drive intestinal fibrosis in Crohn's disease strictures. *Cell*. 2025;188(23):6536-53.e26.
18. Mukhatayev Z, et al. CTHRC1: An Emerging Hallmark of Pathogenic Fibroblasts in Lung Fibrosis. *Cells*. 2024;13(11):946.
19. Lin S, et al. Milk fat globule-epidermal growth factor 8 (MFGE8) prevents intestinal fibrosis. *Gut*. 2024;73(7):1110-23.
20. Wight TN, et al. Versican-A Critical Extracellular Matrix Regulator of Immunity and Inflammation. *Front Immunol*. 2020;11:512.
21. Bukong TN, et al. Versican: a novel modulator of hepatic fibrosis. *Lab Invest*. 2016;96(3):361-74.
22. Wang J, et al. STAT5/VCAN/PI3K signaling pathway promotes fibroblast activation and lung fibrosis. *Cell Signal*. 2025;134:111970.
23. Estany S, et al. Lung fibrotic tenascin-C upregulation is associated with other extracellular matrix proteins and induced by TGFβ1. *BMC Pulm Med*. 2014;14:120.
24. Rudman-Melnick V, et al. Gucy1α1 specifically marks kidney, heart, lung and liver fibroblasts. *Sci Rep*. 2024;14(1).
25. Gui Y, et al. Calponin 2 harnesses metabolic reprogramming to determine kidney fibrosis. *Mol Metab*. 2023;71:101712.
26. Moorhouse AJ, et al. The BASP1 transcriptional corepressor modifies chromatin through lipid-dependent and lipid-independent mechanisms. *iScience*. 2022;25(8):104796.
27. He W, et al. From inflammation to remodelling: A novel BASP1(+) monocyte subset as a catalyst for acute aortic dissection. *J Adv Res*. 2025.

28. Storch J, Corsico B. The Multifunctional Family of Mammalian Fatty Acid-Binding Proteins. *Annu Rev Nutr*. 2023;43:25-54.
29. Kim SW, et al. Downregulation of Heat Shock Protein 72 Contributes to Fibrostenosis in Crohn's Disease. *Gut Liver*. 2023;17(6):905-15.
30. Pang X, et al. Targeting integrin pathways: mechanisms and advances in therapy. *Signal Transduct Target Ther*. 2023;8(1):1.
31. Friedman CE, et al. HOPX-associated molecular programs control cardiomyocyte cell states underpinning cardiac structure and function. *Dev Cell*. 2024;59(1):91-107.e6.
32. Jerala M, et al. Thrombospondin 2, matrix Gla protein and digital analysis identified distinct fibroblast populations in fibrostenosing Crohn's disease. *Sci Rep*. 2024;14(1):13810.
33. Gelbmann CM, et al. Strictures in Crohn's disease are characterised by an accumulation of mast cells colocalised with laminin but not with fibronectin or vitronectin. *Gut*. 1999;45(2):210-7.
34. Jacobs I, et al. Fibrostricturing Crohn's Disease Is Marked by an Increase in Active Eosinophils in the Deeper Layers. *Clin Transl Gastroenterol*. 2024;15(7):e00706.
35. Wang Z, et al. Periostin: an emerging activator of multiple signaling pathways. *J Cell Commun Signal*. 2022;16(4):515-30.
36. Qiao B, et al. The role of periostin in cardiac fibrosis. *Heart Fail Rev*. 2023;29(1):191-206.
37. Li G, et al. Periostin mediates vascular smooth muscle cell migration through the integrins alphavbeta3 and alphavbeta5 and focal adhesion kinase (FAK) pathway. *Atherosclerosis*. 2010;208(2):358-65.
38. Lee MY, et al. Serum response factor regulates smooth muscle contractility via myotonic dystrophy protein kinases and L-type calcium channels. *PLoS One*. 2017;12(2):e0171262.
39. Galdos FX, et al. Cardiac ACTN2 enhancer regulates cardiometabolism and maturation. *Nature Cardiovascular Research*. 2024;3(6):616-8.
40. Htet M, et al. A transcriptional enhancer regulates cardiac maturation. *Nat Cardiovasc Res*. 2024;3(6):666-84.
41. Szulik MW, et al. SMYD1a protects the heart from ischemic injury by regulating OPA1-mediated cristae remodeling and supercomplex formation. *Basic Res Cardiol*. 2023;118(1):20.
42. Fang D, et al. Targeting PTGDS inhibits pro-inflammatory fibroblasts associated with skin fibrosis in systemic sclerosis. *Rheumatology (Oxford)*. 2025;64(10):5551-61.
43. Hu L, et al. SND1, a novel co-activator of HIF1α, promotes tumor initiation in PyMT-induced breast tumor. *Febs j*. 2023;290(24):5759-72.
44. Kim Y. The impact of altered lamin B1 levels on nuclear lamina structure and function in aging and human diseases. *Curr Opin Cell Biol*. 2023;85:102257.
